# Ratiometric upconversion nanothermometry with dual emission at the same wavelength decoded via a time-resolved technique

**DOI:** 10.1038/s41467-019-13796-w

**Published:** 2020-01-07

**Authors:** Xiaochen Qiu, Qianwen Zhou, Xingjun Zhu, Zugen Wu, Wei Feng, Fuyou Li

**Affiliations:** Department of Chemistry & State Key Laboratory of Molecular Engineering of Polymers, Fudan University, 2005 Songhu Road, Shanghai, 200433 P. R. China

**Keywords:** Fluorescence imaging, Imaging techniques, Biosensors

## Abstract

The in vivo temperature monitoring of a microenvironment is significant in biology and nanomedicine research. Luminescent nanothermometry provides a noninvasive method of detecting the temperature in vivo with high sensitivity and high response speed. However, absorption and scattering in complex tissues limit the signal penetration depth and cause errors due to variation at different locations in vivo. In order to minimize these errors and monitor temperature in vivo, in the present work, we provided a strategy to fabricate a same-wavelength dual emission ratiometric upconversion luminescence nanothermometer based on a hybrid structure composed of upconversion emissive PbS quantum dots and Tm-doped upconversion nanoparticles. The ratiometric signal composed of two upconversion emissions working at the same wavelength, but different luminescent lifetimes, were decoded via a time-resolved technique. This nanothermometer improved the temperature monitoring ability and a thermal resolution and sensitivity of ~0.5 K and ~5.6% K^−1^ were obtained in vivo, respectively.

## Abstract (web summary)

Traditional ratiometric temperature monitoring is challenging due to the variation in tissue absorption and scattering of different wavelengths. Here, the authors show improved accuracy by using emission at the same wavelength, but different luminescent lifetimes decoded by a time-resolved technique.

## Introduction

Luminescent nanothermometry has attracted considerable interest as a novel type of sub-micrometric thermal reading due to its strong temperature-dependent luminescence in nanosize^1–5^. Benefitting greatly from its high resolution, high sensitivity, and noninvasiveness, it is widely applied in biology^6–10^, thermodynamics^11–16^, and nanomedicine^17–21^. Luminescent thermal reading can provide temperature measurements in inflamed regions in vivo via contactless and noninvasive luminescence bioimaging^22,23^. Recently, luminescent nanothermometry has been used to control hyperthermia in photothermal therapy, which utilizes photothermal agents to generate heat to kill cancer cells under near-infrared (NIR) laser irradiation, to minimize the damage to surrounding normal tissues^24–26^. Upconversion luminescence (UCL) bioimaging can reduce auto-fluorescence from biological systems owing to its anti-Stokes process^27–29^. To date, several ratiometric upconversion luminescent nanothermometers, which are based on lanthanide doped upconversion nanoparticles^16,30^ (UCNPs) or triplet-triplet annihilation^31^, have been applied to monitor temperature in small animals. Unfortunately, the thermal sensitive signals of these upconversion luminescent nanothermometers are still located in the visible region, which limit their detection depth due to high absorption and scattering which originate from complex tissues. Meanwhile, the accurate microenvironment temperature monitor in vivo remains challenging owing to the complex interaction between light and tissue. The basic effects are shown in the following formula:

1 $$I_{{\mathrm{in}}\;{\mathrm{vivo}}} = f\left( p \right) \cdot f\left( c \right) \cdot f\left( a \right) \cdot f(s) \cdot f(x)$$

In this formula, *f*(*p*), *f*(*c*), *f*(*a*), *f*(*s*), and *f*(*x*) correspond to excitation power density, sensor concentration in vivo, light absorption, light scattering and other influencing factors, respectively. Dual emission ratio luminescent nanothermometers show resolvable emission from two different emitters, which can be used as a self-referencing ratio. The temperature-sensitive signal change can be measured using ratio instead of absolute photoluminescence intensities, to reduce the impact of extrinsic factors such as sensor concentration (*f*(*c*)), fluctuations of excitation power density (*f*(*p*)) or other local inhomogeneities that alter absolute intensities. Owing to minimizing the influence of *f*(*p*) and *f*(*c*), dual emission ratio luminescent nanothermometers have been widely developed and have attracted considerable interest^32–37^. However, *f*(*a*) and *f*(*s*) which depend on wavelength cannot be avoided due to the marked difference in wavelength between the ratiometric emissions (Supplementary Table 1). Moreover, on account of the in vivo heterogeneity at different locations, *f* (*a*) and *f* (*s*) are still challenging to simulate in vitro. Taking all these factors into consideration, it is still difficult for different wavelength dual emission ratiometric nanothermometers to eliminate errors originating from tissue absorption and scattering in vivo. Recently, there has been renewed interest in UCL emission of quantum dots (QDs), which can be excited by a broad wavelength light under a low power density^38–41^. Due to the participation of phonons in this UCL process, the UCL emission shows excellent temperature-dependent properties, which make it a suitable candidate for fabricating a nanothermometer.

**Fig. 1:**
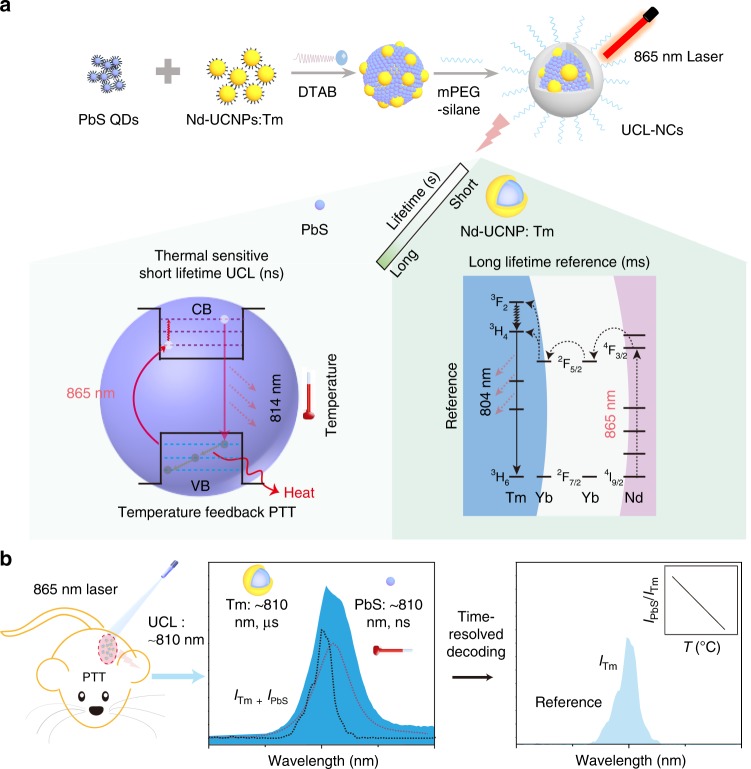
Schematic of UCL-NCs for the same wavelength ratiometric thermometry. **a** Schematic of the formation and illustration of the proposed energy-transfer mechanisms of the UCL-NCs. The UCL-NCs exhibit both long lifetime and short lifetime UCL emission. The temperature-sensitive short lifetime UCL emission is obtained from PbS via a single photon assisted process. The reference long lifetime UCL emission is achieved via fabricating a core–shell-shell Nd-sensitized Tm-UCNP. The heat is produced through a non-radiative process in PbS. **b** Schematic illustration of the same wavelength ratiometric temperature monitoring in vivo. The temperature-sensitive short lifetime UCL emission and the reference long lifetime UCL emission are decoded using a time-resolved technique. The temperature is calculated from the ratio of UCL_PbS_ to UCL_Tm-UCNP_.

In the present study, same wavelength dual emission ratio upconversion luminescent nanothermometry was fabricated and determined in vivo via time-resolved decoding, to minimize the errors originating from different wavelengths in dual emission ratio nanothermometry. Hybrid upconversion nanoclusters (UCL-NCs), which contain a temperature responsive NIR UCL unit (PbS QDs, with UCL emission at 814 nm) and a reference unit (NaYbF_4_:0.5%Tm@NaYF_4_:10%Yb@NaYF_4_:50%Nd, abbreviated to Tm-UCNPs) with very close wavelength emission at 804 nm, were fabricated via an easy evaporation-induced self-assembly method (Fig. 1a). Under the excitation of an 865 nm laser, both PbS QDs and Tm-UCNPs achieved a UCL emission around 810 nm, which could not be separated from each other under optical resolution according to Rayleigh’s criterion^42^. The temperature responsive and reference signals at almost the same wavelength with different lifetimes were decoded via time-resolved technology (Fig. 1b). It is worth mentioning that NIR UCL emission and excitation in the biological window makes it applicable for bio-temperature monitoring in vivo. Moreover, this nanothermometer exhibited thermal sensitivity up to 5.6% K^−1^ and a thermal resolution of ~0.5 K at approximately 45 ^o^C. This dual emission ratiometric nanothermometry demonstrated good temperature measurement ability in tissue, and temperature mapping in tumor region in mice was performed on mice in vivo.

## Results

### Characterization of UCL-NCs

**Fig. 2:**
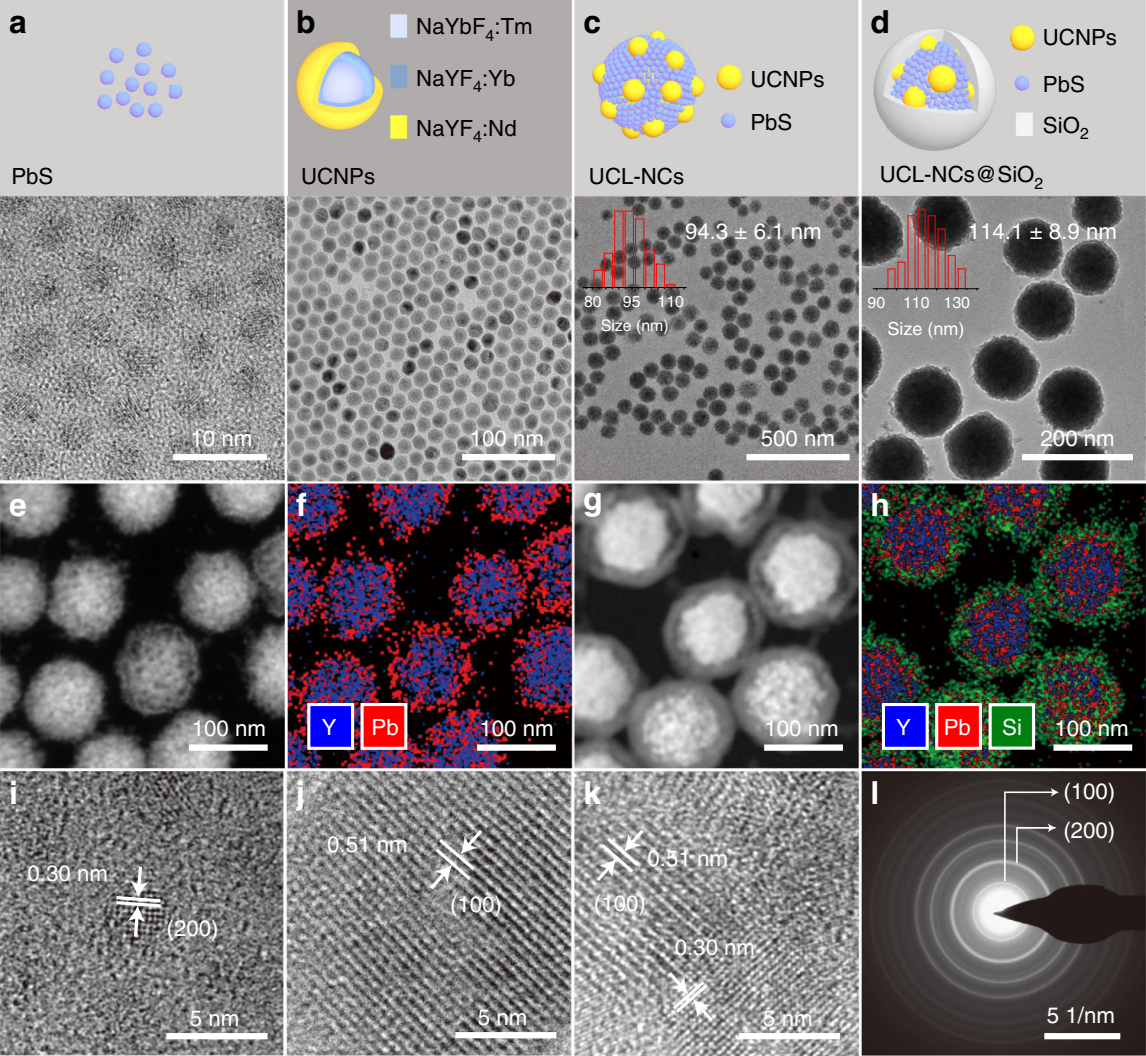
Characterization of UCL-NCs. TEM images of **a** PbS QDs, **b** core-shell-shell Nd sensitized Tm-UCNPs, **c** UCL-NCs and **d** mPEG-silane functionalized silica-coated UCL-NCs. Images of dark-field scanning TEM and corresponding EDS elemental mapping of (**e**, **f**) UCL-NCs and (**g**, **h**) UCL-NCs@SiO_2_. HR-TEM images of (**i**) PbS QDs, (**j**) core-shell-shell Nd sensitized Tm-UCNPs and (**k)** UCL-NCs. **l** Electron diffraction image of UCL-NCs.

In order to construct a ratiometric temperature sensor, broad wavelength (850–915 nm) excitation PbS QDs with a UCL emission around 810 nm were firstly synthesized using a hot injection method according to literature^43^. A transmission electron microscopy (TEM) image (Fig. 2a) revealed the uniform and monodisperse size (3.5 ± 0.2 nm) of the PbS QDs. To obtain a reference UCL emission around 810 nm, Tm-doped UCNPs (β-NaYbF_4_:0.5%Tm, abbreviated to Tm-UCNPs) ~12 nm in size (Supplementary Fig. 1a) were prepared using a previously published method with modification^44^. In order to adjust the excitation wavelength to 850–915 nm, a ~0.7 nm transition layer (NaYF_4_:10%Yb) (Supplementary Fig. 1b) and a ~1.6 nm Nd-doped shell (NaYF_4_:50%Nd) (Fig. 2b) were epitaxially grown via a thermal decomposition method, sequentially. The UCL-NCs were then synthesized by an evaporation-induced self-assembly method according to previous reports^45,46^. The TEM image (Fig. 2c) shows that the PbS QDs and Tm-UCNPs were successfully assembled into spherical clusters and the resulting UCL-NCs had a uniform size of 94.3 ± 6.1 nm. In order to improve biocompatibility and bio-stability, we encapsulated UCL-NCs with an approximately 10 nm-thick polyethylene glycol (PEG) modified silica shell (Fig. 2d) via a sol-gel process. The Fourier transform infrared (FT-IR) spectrum indicated that PEG was successfully modified on UCL-NCs (Supplementary Fig. 2). Energy-dispersive X-ray spectroscopy (EDS) mappings (Fig. 2e–h) showed a random distribution of Y and Pb over the whole cluster, which suggested the successful assembly of PbS QDs and Tm-UCNPs. For further confirmation, high-resolution transmission electron microscopy (HR-TEM) and electron diffraction were performed. The HR-TEM images (Fig. 2i–k) showed that the lattice fringes with d-spacing were 0.51 and 0.35 nm, which were ascribed to the (100) plane of β-phase NaYbF_4_ and the (200) plane of PbS, respectively. These results were also consistent with those of electron diffraction (Fig. 2l). The stability of UCL-NCs@SiO_2_ in phosphate buffer saline was verified by dynamic light scattering (DLS) measurements, and little change was observed after storage for one week (Supplementary Fig. 3).

### Conventional ratiometric thermometry in vitro

**Fig. 3:**
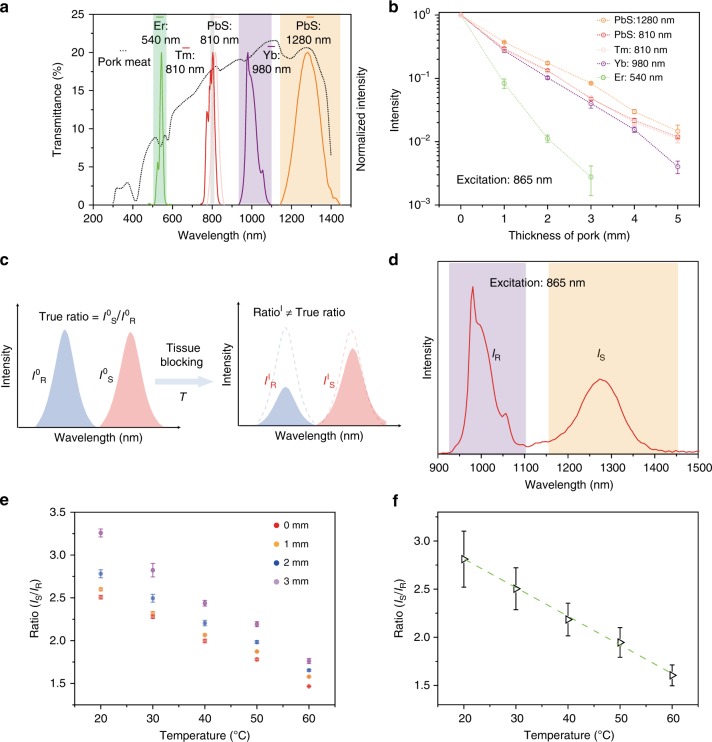
Errors caused by wavelength difference. **a** Transmittance spectrum (left axis) of 1 mm pork and emission spectra (right axis) of Er-UCNPs, Tm-nanoparticles and PbS QDs. **b** Normalized intensities obtained from Er-UCNPs, Tm-nanoparticles and PbS QDs with respect to thickness of the pork tissue slice under excitation of 865 nm laser. Each data set and error bars defined as s.d. were based on three measurements by spectrometry. **c** Schematic illustration of conventional luminescent ratiometric thermometry with different wavelength emissions. **d** Stokes emission spectrum of UCL-NCs under excitation of 865 nm laser. *I*_R_ and *I*_S_ are the reference emission (Yb^3+^ emission, 980 nm) and temperature sensitive emission (PbS emission, 1280 nm), respectively. **e** Ratios of *I*_S_ to *I*_R_ at different temperatures with blocking of various pork tissue slice. Data and error bars, defined as s.d., were based on three measurements by spectrometry. **f** A plot of *I*_S_/*I*_R_ versus *T* to calibrate temperature. Average values of *I*_S_/*I*_R_ under different temperatures were obtained from the ratios with blocking due to pork tissue slice of different thickness (0, 1, 2, and 3 mm). Error bars were defined as s.d. Source data are provided as a Source Data file.

As shown in Fig. 3a, the transmittance of tissue at different wavelengths exhibited an obvious variation. We next investigated tissue blocking of various emissions with different wavelengths under excitation at 865 nm (Fig. 3b). The 540 nm emission from Er^3+^ decreased much more rapidly due to severe blocking of tissue in the visible range. Although NIR light undergoes weaker absorption and scattering, different attenuation was observed between 980 nm (Yb^3+^ emission) and 1280 nm (PbS QDs emission). Furthermore, the attenuation difference in these emissions was irregular and unpredictable due to tissue heterogeneity. In contrast, the UCL emissions around 810 nm from PbS QDs and Tm-UCNPs showed a very close attenuation due to their similar wavelength emissions. However, for conventional ratiometric thermometry, a large wavelength difference between the thermal sensitive signal and reference signal was always required in order to separate the two signals easily, which will cause errors when calculating the temeprature (Fig. 3c). To identify the effect of tissue on the temperature evaluation standard curve for conventional ratiometric thermometry, we compared the Stokes emission ratio (Fig. 3d) of UCL-NCs covered with pork tissue slices of various thickness at different temperatures. As shown in Fig. 3e, with the added thickness of the pork, the deviation of the true temperature evaluation standard curve increased. Importantly, the ratio at different temperatures changed variously and irregularly with thickness of the pork. It should be mentioned that the biological composition also varied at different locations. Moreover, the true depth of tissue was also difficult to determine due to the wide distribution of nanostructures in vivo. These factors made it almost impossible to simulate the real in vivo situation using in vitro nanothermometry. A working ratio curve and standard deviation were then calculated based on the ratios obtained from pork tissue slices of different thickness (Fig. 3f), and the ratio was obtained using the following equation:

2 $${\mathrm{Ratio}}\;{\mathrm{ = }} - \!{\mathrm{0}}{\mathrm{.029}}\;T{\mathrm{ + 3}}{\mathrm{.376}}$$

The thermal sensitivity (*S*_R_) and temperature uncertainty (*σT*) are defined as follows:

3 $$S_R = \frac{{{\mathrm{dRatio}}}}{{{\mathrm{d}}T}}\frac{1}{{{\mathrm{Ratio}}}}$$

4 $$\sigma T = \frac{{\sigma R}}{{S_R}\cdot{R}}$$

where dRatio/d*T* can be botained from the slope of the linear fit represented in Fig. 3f, and *σR* is the standard deviation of the ratio. The thermal sensitivity and temperature uncertainty of this conventional thermometer at 50 ^o^C was ~1.5% K^−1^ and 5.6 K, respectively. Taken together, these results suggest that it remains difficult for different wavelength ratiometric thermometry to monitor in vivo temperature accurately.

### Same wavelength dual emission ratiometric thermometry in vitro

**Fig. 4:**
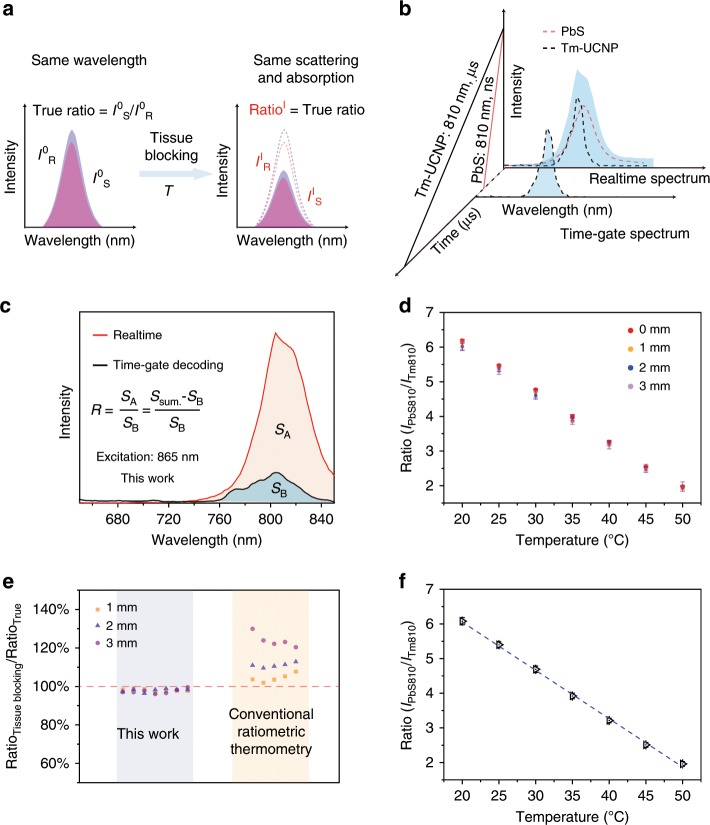
Evaluation of temperature monitoring ability of UCL-NCs. **a** Schematic illustration of conventional ratiometric thermometry with the same wavelength emission. **b** Schematic diagram of UCL signals separation via time-resolved decoding. **c** UCL emission signals decoding of UCL-NCs using time-resolved spectrometer. **d** The same wavelength ratio of *I*_PbS_ to *I*_Tm_ obtained using time-resolved spectrometry at different temperatures and blocking with pork tissue slice of various thickness. Data and error bars, defined as s.d., were based on three measurements with an excitation of 865 nm laser on spectrometry. **e** Ratio values deviation due to blocking with pork tissue slice of different thickness compared with ratio signals measured without pork tissue slice blocking using the same wavelength or conventional ratiometric thermometry. **f** A plot of *I*_PbS810_/*I*_Tm810_ versus T to calibrate the temperature. Average values of *I*_PbS810_/I_Tm810_ under different temperatures were obtained to fit the calibration curve based on the spectra with blocking due to pork tissue slice of different thickness. Error bars were defined as s.d. Source data are provided as a Source Data file.

To identify whether the UCL emissions of PbS QDs and Tm-UCNPs was suitable for constructing a ratiometric thermometer, the temperature and excitation light power density dependent UCL spectra of PbS QDs and Tm-UCNPs were investigated in detail (See details in Supplementary Note 1). These results revealed that both *I*_PbS_ and *I*_Tm_ were dependent on temperature (Supplementary Fig. 4). Moreover, the power density of excitation light did not affect the ratio of *I*_PbS_ to *I*_Tm_ under a low excitation power density of sub-1 W cm^−2^ (Supplementary Figs. 5 and 6). The temperature monitoring ability of the same wavelength ratiometric thermometry (Fig. 4a) was subsequently investigated using the UCL emission of UCL-NCs. Although the same band UCL emission of PbS QDs and Tm-UCNPs in UCL-NCs cannot be separated using filters on conventional spectroscopic devices, these two signals were decoded via the time-resolved technique due to the different luminescence lifetimes (Fig. 4b). Consequently, the temperature calibration curve of UCL-NCs was investigated by performing several heating and cooling cycles on a time-resolved spectrometer. The real-time spectrum was first detected to obtain *I*_Sum_. Then a 20 μs delayed time-resolved spectrum was acquired to decode the *I*_Tm_ (Fig. 4c). Finally, the ratio was calculated according to Ratio = (*I*_Sum_ − *I*_Tm_)/*I*_Tm_. Pork tissue slice covering of different thickness was used to simulate the temperature elevation of UCL-NCs in biological tissue. The results showed no obvious deviation (Fig. 4d). Following pork tissue slice covering of different thickness, both *I*_Sum_ and *I*_Tm_ showed a sharp decline due to the absorption and scattering originating from the complex tissues. To evaluate the ratio deviations due to tissue blocking, Ratio_1–3 mm_/Ratio_0 mm_ were calculated. As shown in Fig. 4e, for the same wavelength ratiometric thermometry, Ratio_1–3 mm_/Ratio_0 mm_ was close to 1 under different temperatures. By contrast, when using conventional ratiometric thermometry, the maximum values of Ratio_1 mm_/Ratio_0 mm_, Ratio_2 mm_/Ratio_0 mm_ and Ratio_3 mm_/Ratio_0 mm_ were 108%, 110%, and 130%, respectively. A linear fit of the experimental data was performed based on the ratios obtained from pork tissue slice covering of different thickness (Fig. 4f), and the following equation was obtained:

5 $${\mathrm{Ratio}}\;\left( {\frac{{I_{{\mathrm{Sum}}} - I_{{\mathrm{Tm}}}}}{{I_{{\mathrm{Tm}}}}}} \right) = - {\mathrm{0}}{\mathrm{.139}}\;T + {\mathrm{8}}{\mathrm{.851}}$$

Based on these results, a thermal sensitivity of 5.6% K^−1^ and a thermal resolution of ~0.5 K are obtained at approximately 45 ^o^C. These results suggested that the same wavelength dual emission ratiometric thermometry had a more accurate temperature monitoring ability than conventional ratiometric thermometry.

### Temperature monitoring in vivo

**Fig. 5:**
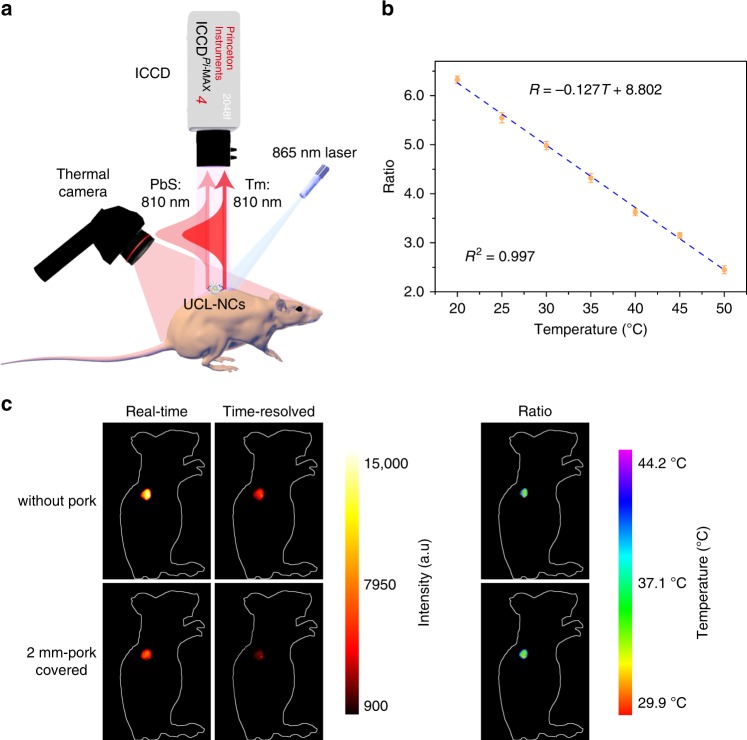
Temperature monitoring in vivo using UCL-NCs. **a** Schematic diagram of surficial and intratumoral monitoring in vivo. **b** The standard curve for temperature evaluation in vivo measured with the same wavelength ratiometric probe based on UCL-NCs with the excitation of 865 nm laser using a home-built time-gated bioimaging system. **c** Real-time, time-gated and ratio UCL imaging without or with a 2 mm-pork tissue slice covering in vivo under irradiation by a 865 nm laser (0.5 W cm^−2^) for 30 s. The ratio imaging figure was obtained using image arithmetic (Ratio = (Real time − Time gated)/Time gated). Source data are provided as a Source Data file.

The same wavelength dual emission ratiometric thermometer based on UCL-NCs was tested for intratumoral temperature monitoring in vivo. The response to temperature change was fully reversible and no significant differences were observed, suggesting that the structure of UCL-NCs were hardly affected by repeated heating (Supplementary Fig. 7). DLS measurements were also performed at several heating cycles (Supplementary Fig. 8). To further verify the thermal stability, TEM images and EDS elemental mapping of UCL-NCs@SiO_2_ after warming-cooling cycles were performed, and the results indicated no obvious changes (Supplementary Fig. 9). These results reveal that the UCL-NC@SiO_2_ structure had good thermal stability. The standard temperature curve of UCL-NCs@SiO_2_ for practical temperature feedback in vivo was calibrated in a home-built time-gated bioimaging system (Fig. 5a). Pork covering was used to simulate the condition in vivo. The dependence of ratio on temperature is depicted in Fig. 5b, which shows a linear numeric relationship. The data points were well fitted as shown in the following equation:

6 $${\mathrm{Ratio}}\;{\mathrm{ = }} - \!{\mathrm{0}}{\mathrm{.127}}\;T{\mathrm{ + 8}}{\mathrm{.802}}$$

On the other hand, when PbS QDs were excited with an 865 nm laser, both radiative and nonradiative de-excitation processes were expected to occur. Thus, PbS partially converted the absorbed optical energy into heat, which caused an increment in temperature (Supplementary Fig. 10 and Supplementary Note 2). To verify the temperature monitoring ability of the same wavelength ratiometric thermometry in vivo, intratumoral temperature was monitored without or with a 2 mm-pork covering after irradiation with an 865 nm laser (500 mW cm^−2^) for 30 s. Although the intensity of both real-time and time-resolved imaging exhibited marked attenuation after covering with a 2 mm-pork tissue slice, the ratio showed little difference (Fig. 5c). The calculated temperatures were 35.5 ^o^C and 35.2 ^o^C, respectively. This result indicated that the same wavelength dual emission ratiometric upconversion thermometry showed excellent temperature monitoring ability and the errors originating from tissue blocking can be reduced. Temperature feedback photothermal therapy was then performed in nude mice and the intratumoral temperatures were monitored in vivo using UCL-NCs (Supplementary Fig. 11 and Supplementary Note 3). In addition, surficial temperature was monitored using a thermal camera during treatment (Supplementary Figs. 12 and 13). During the therapy, intratumoral temperature showed a sharp increment and increased as high as 47 ^o^C, in contrast to this, surficial temperature increased by only 3 ^o^C approximately (Supplementary Fig. 14). Tumor sizes and survival of the treated and control groups were investigated (Supplementary Figs. 15 and 16), and the tumors were finally eliminated and the mice survived for over 40 days in the treatment group. Taken together, these results conclusively indicated that the UCL-NC probe was capable of thermometry in vivo to monitor intratumoral temperature.

## Discussion

In summary, we demonstrated a same wavelength dual emission ratio upconversion luminescent nanothermometry system, which exhibited more precise temperature detection in vivo and was used to monitor intratumoral temperature. Under the excitation of 865 nm laser, PbS QDs exhibited a short lifetime temperature response UCL emission around 810 nm via a single photon process and the Nd-sensitized Tm-UCNPs exhibited a long lifetime reference UCL signal around 810 nm. These two UCL emissions at the same wavelength were separated via time-resolved decoding due to their different lifetimes. The same wavelengths for excitation and emission ratiometric nanothermometry based on UCL-NCs showed improved temperature measurement accuracy in vivo, and a temperature uncertainty in vivo of 0.5 K is obtained. It is worth noting that the thermal sensitivity of this hybrid system was up to 5.6% K^−1^. Furthermore, the NIR excitation and emission have advantages in biological applications. After modification with PEG, this hybrid nanostructure material was used in intratumoral temperature monitoring in vivo during photothermal therapy. Thus, the present study provided a more accurate upconversion luminescent nanothermometer and a new method for the construction of dual emission luminescent nanothermometers.

## Methods

### Synthesis of PbS QDs

The synthesis of PbS QDs was according to a previously published procedure. The lead precursor solution was prepared as follows: briefly, 446 mg lead oxide, 1.5 mL OA and 20 mL ODE were added to a 50 mL flask. The solution was heated to 120 ^o^C and degassed under vacuum until the solution became transparent. The solution was then heated to the injection temperature of 125 °C under N_2_. In addition, 200 μL of bis(trimethylsilyl)sulfide was dissolved in 5 mL ODE, the mixture was degassed under vacuum and thoroughly stirred for 1 h at room temperature. The sulfur precursor solution was then rapidly injected into the lead oleate solution under vigorously stirring. After 1 min, the reaction was cooled to room temperature. The synthesized QDs were washed three times by sequential precipitation with acetone and re-dispersed in toluene. The QDs were dispersed in toluene to form stock solutions with concentrations of 100–150 mg mL^−1^ and stored at 4 ^o^C.

### Synthesis of NaYbF_4_:Tm core nanoparticles

Tm-doped NaYbF_4_ nanoparticles were prepared according to a previously described procedure. In brief, YbCl_3_ (0.995 mmol) and TmCl_3_ (0.005 mmol) were added to a mixture of 6 mL OA and 15 mL ODE in a 100 mL three-neck round-bottom flask. The resulting mixture was heated to 140 ^o^C and degassed under vacuum for 30 min to remove the water solvent. The mixture was cooled to 90 ^o^C. Subsequently, 0.1 g NaOH was added and degassed in vacuo for 45 min, followed by the addition of 0.148 g NH_4_F. After purging with N_2_, the mixture was heated to 300 ^o^C and left for 1 h. The synthesized core nanoparticles were washed three times by sequential precipitation with ethanol and re-dispersed in cyclohexane.

### Synthesis of NaYbF_4_:Tm@NaYF_4_:10%Yb core-shell nanoparticles

The preparation of core-shell nanoparticles was according to a previously described protocol. 0.45 mmol Y(CF_3_COO)_3_, 0.05 mmol Yb(CF_3_COO)_3_, 0.5 mmol CF_3_COONa and the as prepared NaYbF_4_:Tm nanoparticles were added to a mixture of OA (5.64 g) and ODE (5.04 g) in a three-necked flask. The mixture was degassed under vacuum at 110 ^o^C for 30 min and then heated to 300 ^o^C under a N_2_ atmosphere for another 30 min. The resulting core–shell nanoparticles were precipitated by the addition of 20 mL ethanol, collected by centrifugation and re-dispersed in cyclohexane.

### Synthesis of NaYbF_4_:Tm@NaYF_4_:10%Yb@NaYF_4_:50%Nd core−shell–shell nanoparticles (Tm-UCNPs)

Core–shel–shell nanoparticles were synthesized using the same protocol as that for core–shell nanoparticles, except that 0.5 mmol Y(CF_3_COO)_3_ and 0.5 mmol Nd(CF_3_COO)_3_ and the as-prepared NaYbF_4_:Tm@NaYF_4_:10%Yb core-shell nanoparticles were added.

### Synthesis of UCL nanoclusters (UCL-NCs)

The UCL-NCs were synthesized according to a literature method. In brief, 1 mL chloroform containing 8 mg QDs and 2 mg UCNPs was swiftly added to 1 mL DTAB aqueous solution (20 mg mL^−1^). The mixture was vigorously mixed by vortex for 10 s. The solution became transparent after removing the chloroform by blowing N_2_. 5 mL PVP EG solution (2 mM) was injected into the reactor. The mixture was stirred at room temperature for 30 min. The resulting UCL-NCs were collected by centrifugation and stored in ethanol for further synthesis.

### Synthesis of UCL-NCs@SiO_2_

The UCL-NCs@SiO_2_ were synthesized via a modified Stöber method. Typically, the as-prepared UCL-NCs (3 mg) were dissolved in 20 mL ethanol under ultrasound. Five minutes later, 2 mL deionized water and 0.5 mL NH_3_·H_2_O were subsequently added. The solution was stirred at room temperature for 1 h. Fifty microliters of TEOS was added dropwise. After stirring for another 40 min, the resulting UCL-NCs@SiO_2_ were collected by centrifugation and re-dispersed in 2 mL H_2_O.

### Synthesis of UCL-NCs@SiO_2_-PEG

The as-synthesized UCL-NCs@SiO_2_ were dissolved in 15 mL H_2_O, and 50 μL NH_3_·H_2_O and 10 μL TEOS were sequentially added into the mixture. One milliliter of PEG (MW 2000) aqueous solution was dropped in the reaction, when the solution was heated to 70 ^o^C under stirring. After stirring for 3 h at 70 ^o^C, the solution was cooled to 25 ^o^C and stirred for another 12 h. The resulting UCL-NCs@SiO_2_-PEG were isolated by centrifugation and stored in water for further application.

### Temperature monitoring in vitro

To obtain the same wavelength ratiometric temperature calibration curve in aqueous solution, a quartz cuvette containing UCL-NCs@SiO_2_ aqueous dispersion (2 mL, 0.5 mg mL^−1^) was placed in the home-built time-resolved spectrometer or home-built time-resolved bioimaging system (emICCD camera, PI-MAX4-1024B/EM, Princeton Instruments) with an external temperature controller. Aqueous solution was heated to different temperatures ranging from 20 to 50 ^o^C and the corresponding real-time or time-resolved UCL at approximately 810 nm was collected with excitation by a continuous wave (CW) 865 nm laser (100 mW cm^−2^). The ratio of UCL emission peaks centered at 810 nm of the PbS QDs and Tm-UCNPs as a function of temperature were used as the calibration curve for temperature monitoring. Pork covering of different thickness was used to simulate the tissue blocking in vivo. The conventional ratiometric temperature calibration curves were obtained on an FLS-920 fluorescence spectrometer (Edinburgh Instruments) under the irradiation of an 865 nm laser (100 mW cm^−2^).

### Tumor xenografts

Animal procedures were in agreement with the guidelines of the Institutional Animal Care and Use Committee, School of Pharmacy, Fudan University. S180 cells were harvested by incubation with 0.05% trypsin-EDTA when they reached near confluence, and were collected by centrifugation and re-suspended in sterile PBS. Cells (~10^7^ cells per site) in 50 μL of modified Eagle’s medium were then subcutaneously implanted into 4-week-old male athymic nude mice. Photothermal therapy was performed when tumors reached an average diameter of 0.6 cm.

### Eigen temperature monitoring in vivo

Eigen temperature monitoring in vivo was conducted in UCL-NCs@SiO_2_ injected S180 tumor-bearing nude mice. 0–5 W adjustable CW 865 nm lasers were chosen as the excitation source for UCL and photothermal excitation (0.5 W cm^−2^). Real-time and time-gated UCL imaging were performed on a home-built time-resolved bioimaging system (emICCD camera, PI-MAX4-1024B/EM, Princeton Instruments) with an 810 ± 10 nm band-pass filter at different time points. The intratumoral temperature at different time points was calculated by the UCL images with the temperature calibration curve obtained in vitro simulated by pork meat covering.

### Photothermal therapy in vivo

An optical fiber-coupled 865 nm diode-laser (Changchun New Industries Optoelectronics Technology Co., Ltd.) was used to irradiate tumors during the experiments. In the treatment group, 50 μL of UCL-NCs@SiO_2_ (2 mg mL^−1^) aqueous solution was injected intratumorally 2 h before irradiation. For photothermal treatment, the 865 nm laser beam with a diameter of ~10 mm was focused on the tumor area at the power density of 0.5 W cm^−2^ for 3 min. A FLIR E40 thermal imaging camera was used to collect infrared thermal images. Tumor sizes in the treatment group and reference groups (untreated mice, mice irradiated with an 865 nm laser only, and UCL-NCs@SiO_2_ treated mice without 865 nm irradiation) were measured every day after treatment. Each group contained five mice for relative rational evaluation. Tumor sizes were measured using a Vernier caliper. The tumor volume was calculated as follows: volume = (tumor length) × (tumor width)^2^/2. Relative tumor volumes were normalized and were calculated as *V*/*V*_0_ (*V*_0_ is the initial tumor volume).

### Reporting summary

Further information on research design is available in the Nature Research Reporting Summary linked to this article.

## Supplementary information

Supporting information file

Peer Review File

Dataset 1

Reporting Summary

## Data Availability

The data that support the findings of this study are available from the corresponding authors upon reasonable request. The data underlying Figs. 3b, e, f, 4d, f, 5b, and Supplementary Figs. 4c, d, 5d, 6, 8b, 11b, c, 14, 15 are provided with the paper.
