# Supplementary material for: Ratiometric upconversion nanothermometry with dual emission at the same wavelength decoded via a time-resolved technique
Source: Nat Commun. 2020 Jan 7;11:4. doi: 10.1038/s41467-019-13796-w (PMC6946702; doi:10.1038/s41467-019-13796-w)
Supplement: Supplementary file 1 — Supporting information file [file 41467_2019_13796_MOESM1_ESM.docx]

**Supporting Information**

**Ratiometric Upconversion Nanothermometry with Dual Emission at the Same-Wavelength Decoded via a Time-Resolved Technique**

Qiu *et al.*

**Supplementary Methods**

**Materials***:* Yb_2_O_3_ (99.999%), Tm_2_O_3_ (99.999%), Nd_2_O_3_ (99.999%) and Y_2_O_3_ (99.999%) were all purchased from Shanghai Yuelong New Materials Co. Ltd. Oleic acid (OA; 90%), 1-octadecene (ODE; >90%), dodecyltrimethylammonium bromide (DTAB), poly(vinylpyrrolidone) (PVP; MW 55,000), ethylene glycol (EG), bis(trimethylsilyl)sulfide ((TMS)_2_S), sulfur and lead(II) oxide were purchased from Sigma-Aldrich. Absolute ethanol was obtained from Adamas-beta. aqueous ammonia (28 wt%), cyclohexane and chloroform were bought from Sinopharm Chemical Reagent Co., China. Methoxypolyethylene-glycol silane (mPEG-silane; MW 2,000 g/mol) was achieved from Shanghai Ponsure Biotechnology Co., Ltd.

**Characterization**: TEM images, HRTEM images, HAADF-STEM images, and EDX mapping of the nanocrystals were characterized on a Tecnai G2 F20 S-Twin (FEI, America) operating at 200 kV. Fourier-transform infrared spectroscopy was performed using an IR Prestige-21 spectrometer (Shimadzu) from samples in KBr pellets. DLS was carried out on a Malvern Zetasizer Nano ZS system. UCL spectra of UCL-NCs were measured with an Edinburgh LFS-920 fluorescence spectrometer. Infrared thermal images were collected FLIR E40 thermal imaging camera.

**Supplementary Table 1.** Experimental parameters of several typical in vivo nanothermometers. *λ*_Ex._*, λ*_Em._, √, × and S_r_ correspond to excitation wavelength, emission wavelength, yes, no and relative thermal sensitivity, respectively.

| **Material** | ***λ*_Ex._ (nm)** | ***λ*_Em._ (nm)** | **NIR Ex. and Em.** | **Ratio** | **Same Em.** | **S_r_**  **(10^-2^ K^-1^)** | **Ref.** |
| --- | --- | --- | --- | --- | --- | --- | --- |
| PbS/CdS/ZnS QDs | 808 | 1270 | **√** | **×** | **-** | 1.0 | [1] |
| PbS/CdS/ZnS-Nd NPs | 808 | 1060 and 1250 | **√** | **√** | **×** | 2.5 | [2] |
| TTA-Nd-NPs | 635 and 808 | 540 and 1060 | **×** | **√** | **×** | 7.1 | [3] |
| CsUCNP  @C | 980 | 525 and 545 | **×** | **√** | **×** | 1.1 | [4] |
| Er-Yb@Yb-Tm LaF_3_ | 690 | 1000, 1230 and 1550 | **×** | **√** | **×** | 5.0 | [5] |
| UCL-NCs | 865 | 810 and 810 | **√** | **√** | **√** | 5.6 | [This work] |


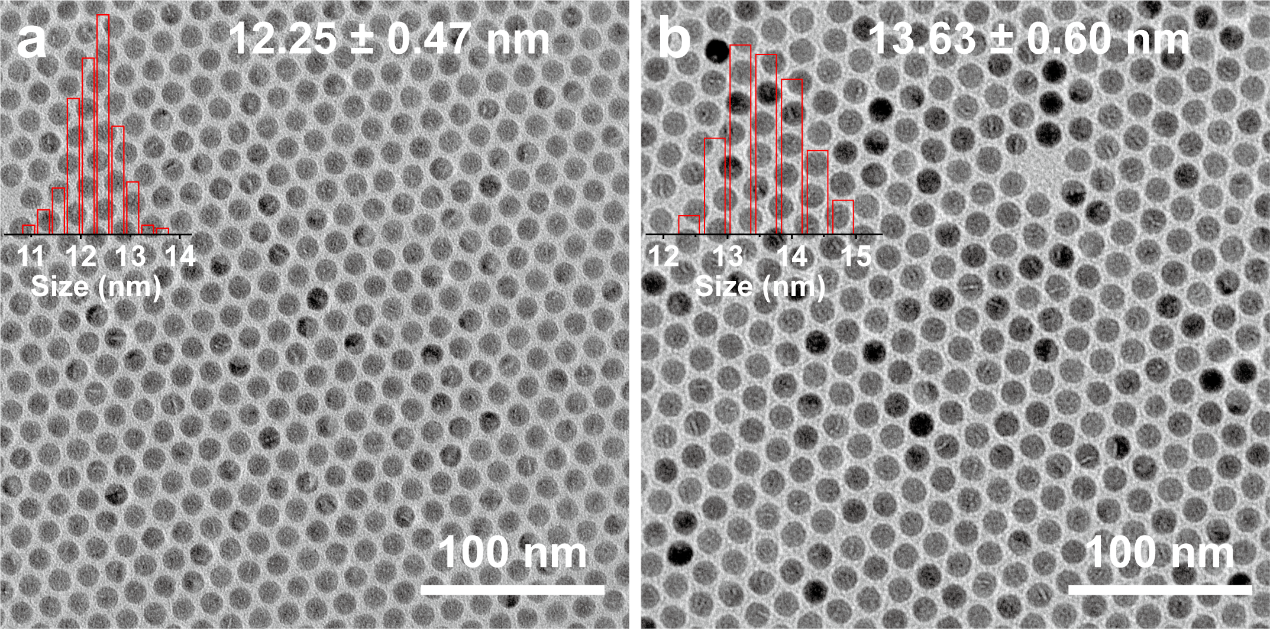


**Supplementary Figure 1.** TEM images of (a) core NaYbF_4_:0.5%Tm, (b) core-shell NaYbF_4_:0.5%Tm@NYF_4_:10%Yb.


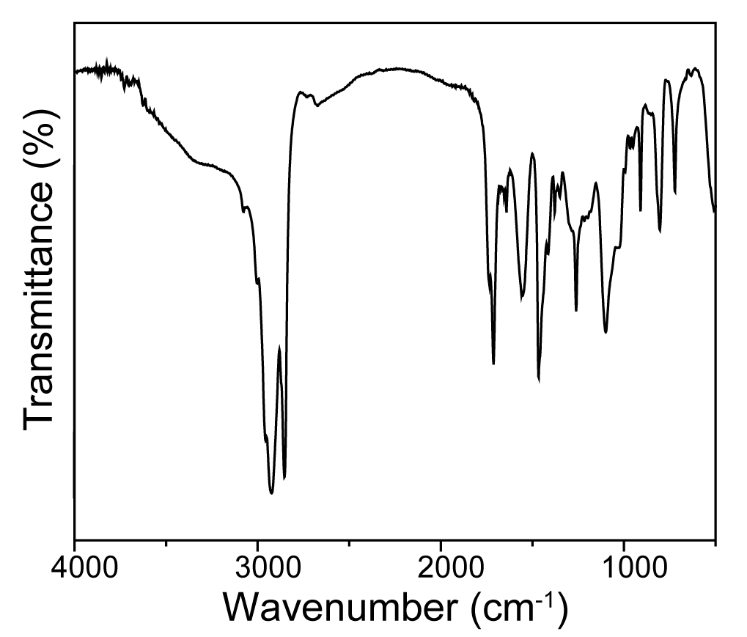


**Supplementary Figure 2.** FI-IR spectrum of PEG functionalized silica-coated UCL-NCs.


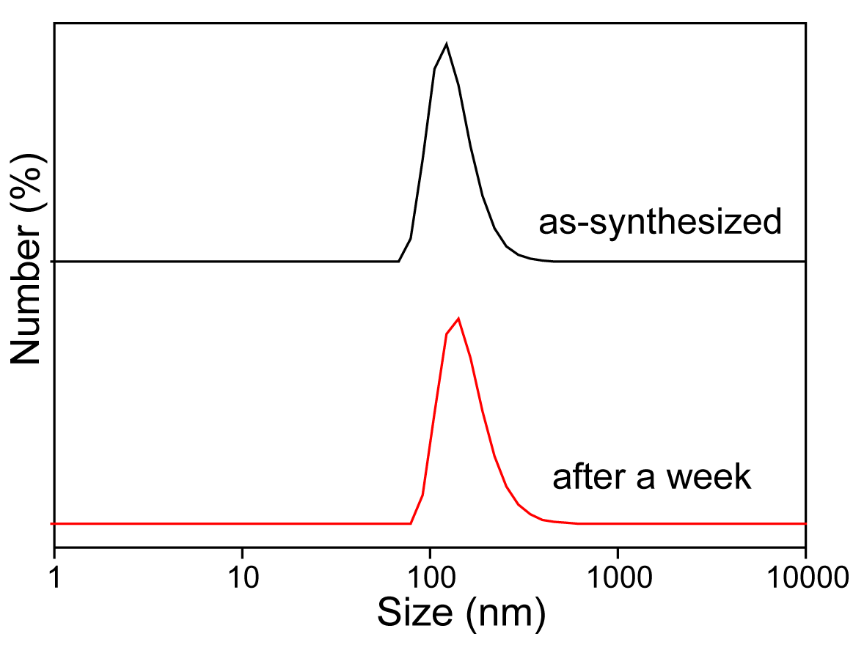


**Supplementary Figure 3.** Disperse stability of UCL-NCs in phosphate buffer saline. DLS size distribution of UCL-NCs before and after storing for a week UCL-NCs.


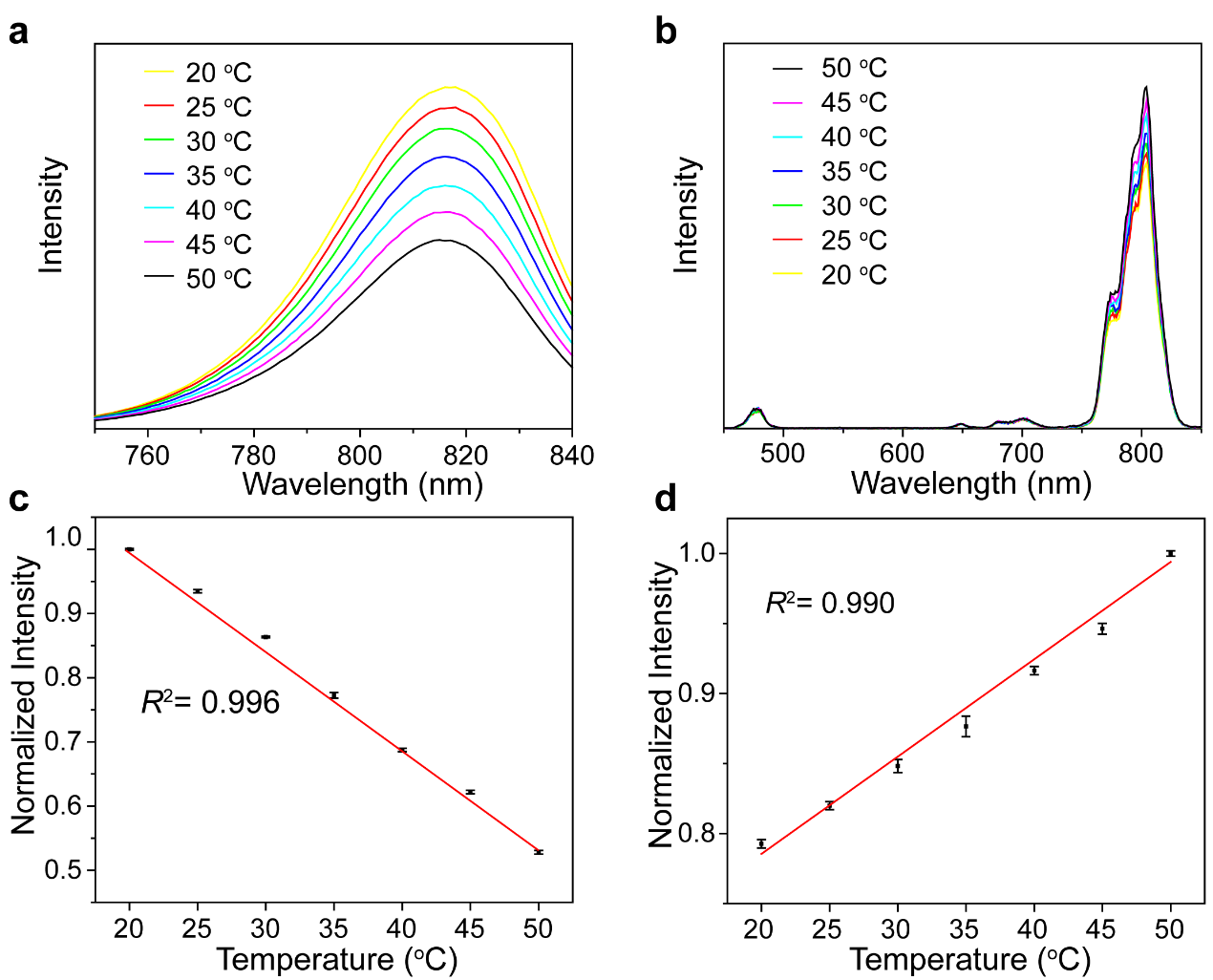


**Supplementary Figure 4.** Temperature dependent UCL spectra of (a) PbS QDs and (b) core-shell-shell Nd sensitized Tm-UCNPs under the excitation of an 865 nm laser (100 mW cm^-2^). A plot of *I*_UCL_ versus *T* of (c) PbS QDs and (d) Tm-UCNPs. Error bars are defined as s.d. Source data are provided as a Source Data file.


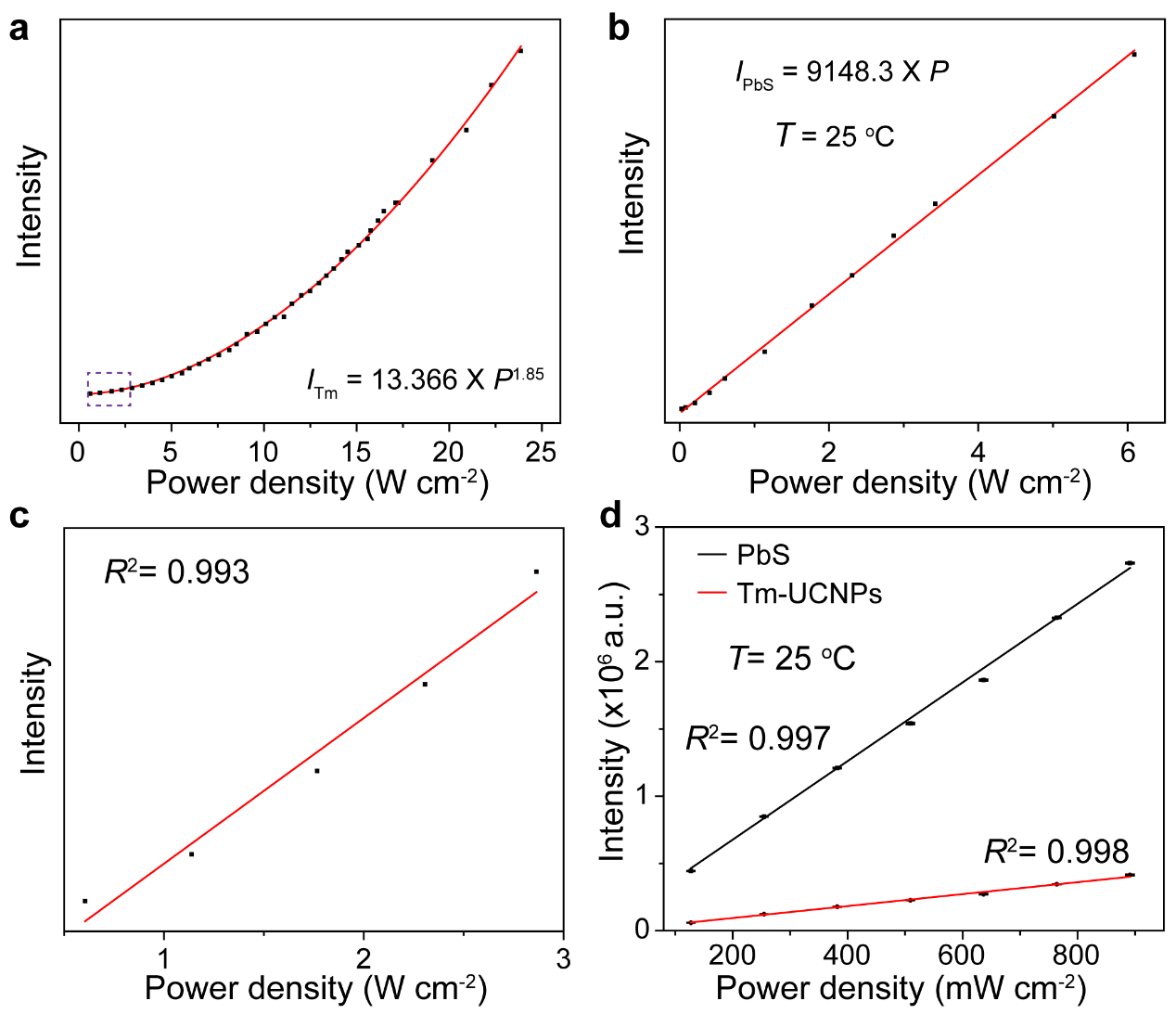


**Supplementary Figure 5.** A plot of *I*_UCL_ versus *P* to investigate the effect of power density to UCL emission of (a) Tm-UCNPs and (b) PbS. (c) A linear dependent in mathematics of *I*_UCL_ versus *P* under a low power density (1-3 W cm^-2^) excitation of Tm-UCNPs. (d) Plots of UCL emission of PbS QDs and Tm-UCNPs as a function of excitation power density of the 865 nm laser. Error bars are defined as s.d. Source data are provided as a Source Data file.

**
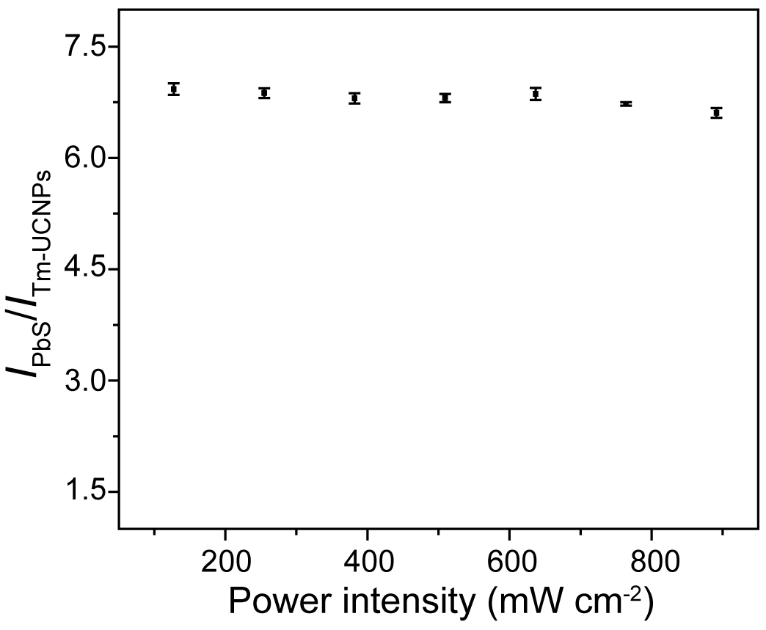
**

**Supplementary Figure 6.** Ratios calculated from UCL emission of *I*_PbS_ to *I*_Tm-UCNPs_ under different power density excitation at 25 ^o^C. Error bars are defined as s.d. Source data are provided as a Source Data file.

**Supplementary Note 1:** **Power density dependent UCL spectra investigation.**

UCL spectra of PbS QDs and Tm-UCNPs aqueous solution were characterized at different temperature in the range from 20 to 50 ^o^C (**Supplementary Figure 4a** and **b**). A clearly decrease is observed in UCL emission intensity centered at approximately 810 nm of PbS QDs under an elevated temperature (**Supplementary Figure 4a**). The normalized UCL emission intensity of PbS QDs (*I*_PbS_) and Tm-UCNPs (*I*_Tm_) is respectively represented as a function of temperature in **Supplementary Figure 4c** and **Supplementary Figure 4d**. The results reveal that both *I*_PbS_ and *I*_Tm_ have a strong dependence on temperature. It is necessary for a ratiometric luminescent thermometer to investigate power dependent spectra both of sensor and reference signals, owing to the reduced power density of excitation light after penetrating the tissue. In principle, for UCL emission, *I*_em_ is proportional to the *n*-th power of the NIR exciting power density. The data demonstrate that the relationship between UCL intensity and excitation power density of PbS QDs is linear (**Supplementary Figure 5b**). For Tm-UCNPs, the result indicates that UCL intensity is quadratic depending on the excitation power (**Supplementary Figure 5b**). However, under a low power excitation, a linear relationship can be fitted in mathematics (**Supplementary Figure 5b inset**). The relationship between excitation power density and *I*_Tm_ was investigated in detail sub-1 W cm^-2^ (100-900 mW cm^-2^) at 25 ^o^C (**Supplementary Figure 5b**). The result reveals that *I*_Tm_ can also be fitted to show linear dependence on excitation power density. It should be noticed that the ratio of *I*_PbS_ to *I*_Tm_ is nearly invariant under different excitation power density at 25 ^o^C (**Supplementary Figure 6**).


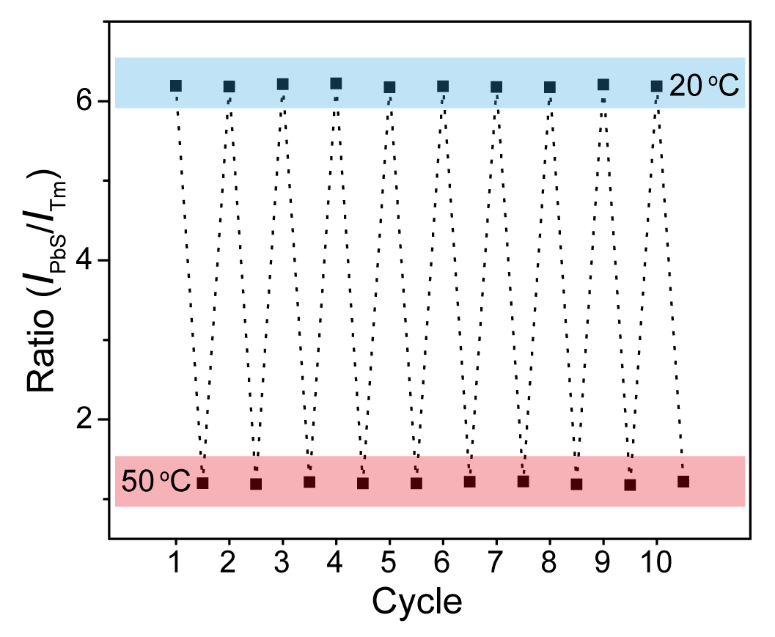


**Supplementary Figure 7.** The reversibility of UCL-NCs tested in continuous warming-cooling cycles. Ratiometric signals were measured at 20 °C and 50 °C using a time gate spectrometer, respectively.


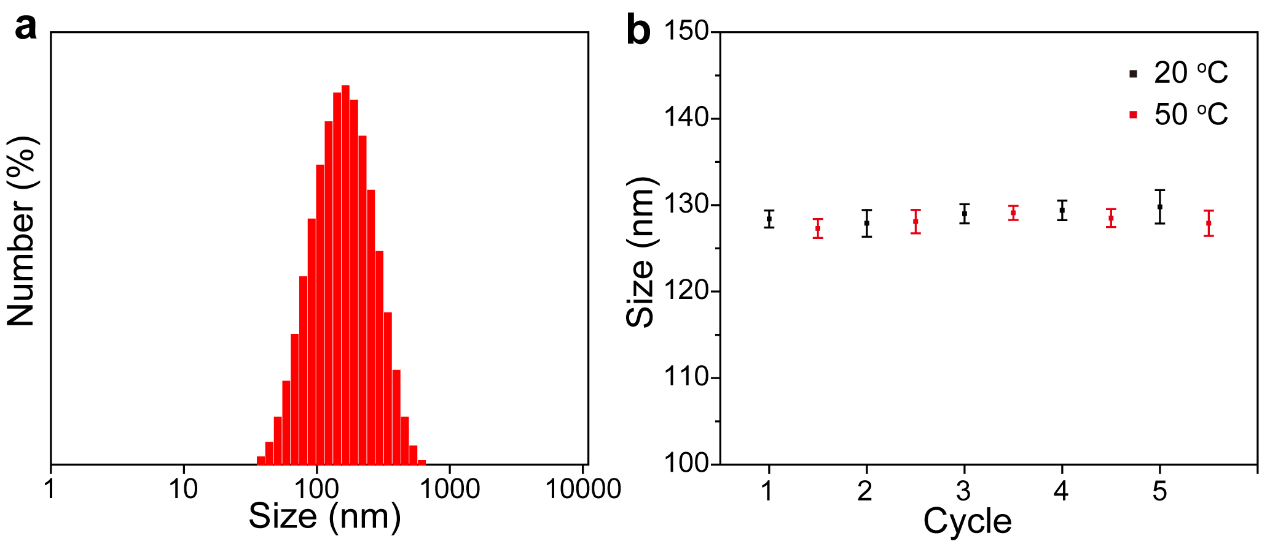


**Supplementary Figure 8.** Heat stability of UCL-NCs evaluation. (a) The hydrodynamic sizes distribution of UCL-NCs measured by DLS analysis. (b) The hydrodynamic size of UCL-NCs measured at 20 or 50 ^o^C in continuous warming-cooling cycles. Error bars are defined as s.d. Source data are provided as a Source Data file.


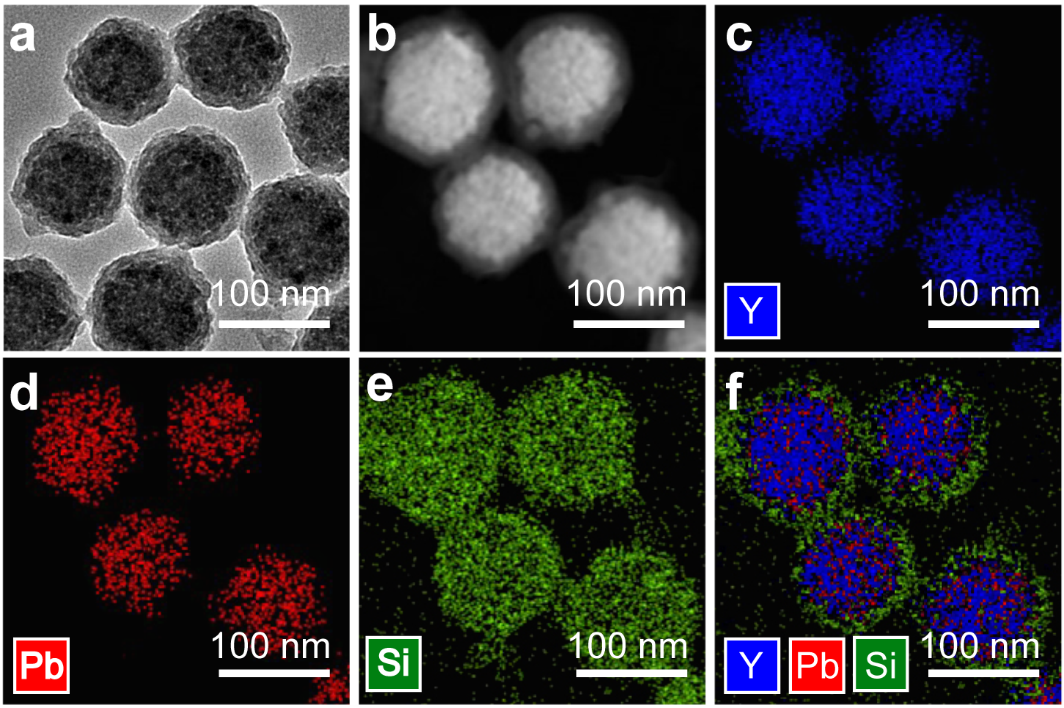


**Supplementary Figure 9.** TEM image, dark-field scanning TEM image and corresponding EDS elemental mapping of (c, d, e and f) UCL-NCs@SiO_2_ after warming-cooling cycles.


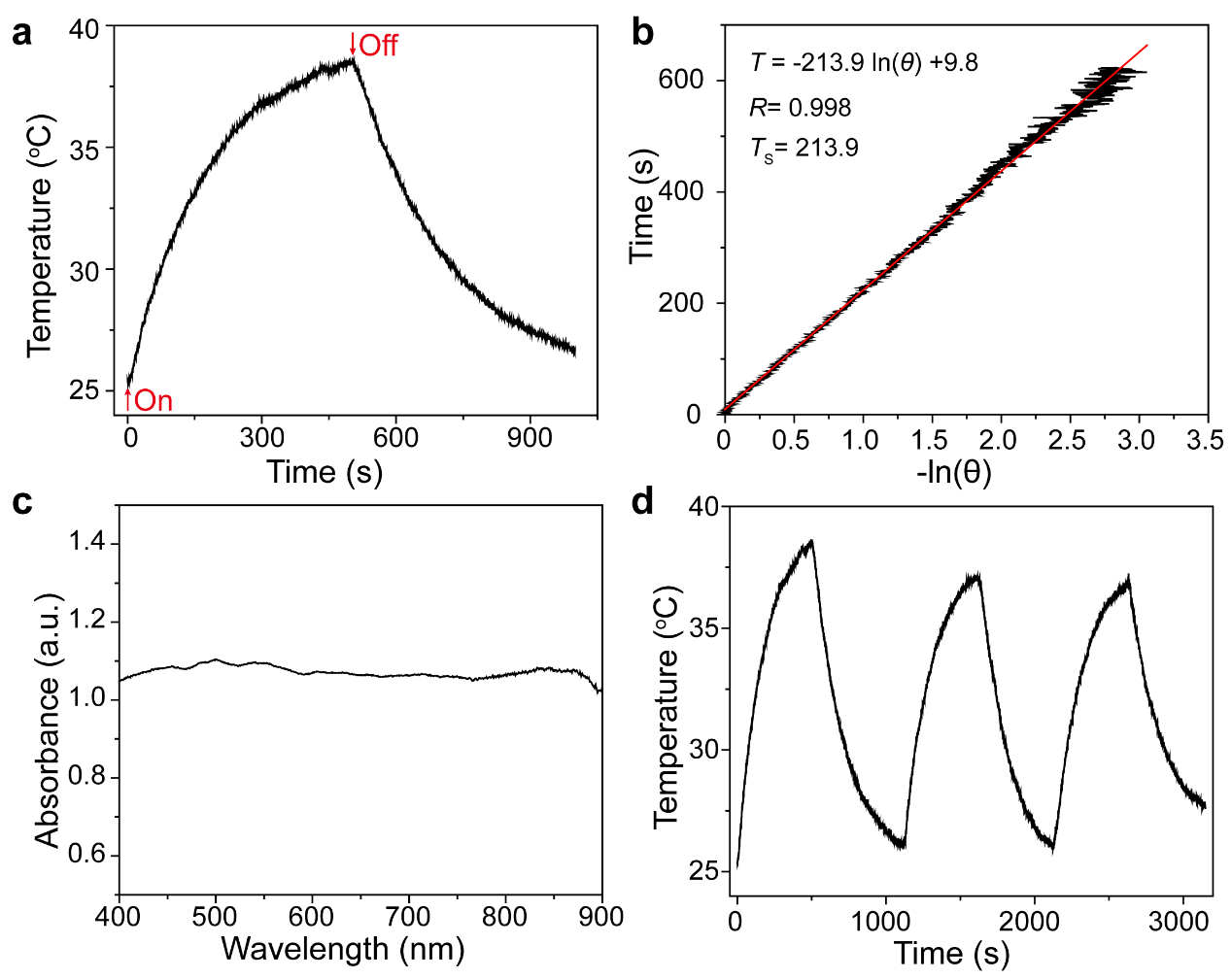


**Supplementary Figure 10.** Characterization of photo thermal properties of UCL-NCs. (a) Temperature rising and cooling curve of UCL-NCs aqueous dispersion under irradiation of an 865 nm laser (0.8 W cm^-2^). During the process, the irradiation lasted for 500 s, and then the laser was shut down. (b) Determination of the time constant for heat transfer from the cooling profile in Figure (a). (c) UV-NIR absorption spectrum of UCL-NCs aqueous dispersion. (d) Heat effect of UCL-NCs aqueous solution over 3 cycles of continuous re-irradiation (0.8 W cm^-2^).

**Supplementary Note 2:** **Calculation of the photothermal conversion efficiency.**

According to the method described in the literature, the total energy conservation for the system can be expressed by Supplementary Equation 1.

$\sum_{\text{i}} \text{m}_{\text{i}}\text{C}_{\text{p}\text{,i}}\frac{\text{d}\text{T}}{\text{d}\text{t}}\text{=}{\text{ }\text{Q}}_{\text{NC}}\text{+}{\text{ }\text{Q}}_{\text{B}}-\text{ }\text{Q}\text{sur}$ (1)

where *m* and *C*_p_ are the mass and heat capacity of water respectively, *T* is the solution temperature, ${\text{ }\text{Q}}_{\text{NC}}$ is the energy induced by UCL-NCs, ${\text{ }\text{Q}}_{\text{B}}$ is the baseline energy induced by the sample cell, and $\text{Q}\text{sur}$ is heat conduction away from the surface by air. ${\text{ }\text{Q}}_{\text{NC}}$ is caused by non-radiative transition of PbS QDs under irradiation of 865 nm laser:

$\text{ }{\text{ }\text{Q}}_{\text{NC}}\text{= }\text{I }\text{(1}-\text{10}^{{-A}_{\text{865}}}\text{) }\text{η}$ (2)

where *I* is the laser power, 𝜂 is the conversion efficiency from incident laser energy to thermal energy, and A_865_ is the absorbance of UCL-NCs at wavelength of 865 nm. On the other hand, ${\text{ }\text{Q}}_{\text{B}}$, expressing heat dissipated from light absorbed by the sample cell, was measured independently to be 8.5 mW using a quartz cuvette containing pure water without UCL-NCs. Moreover, $\text{Q}\text{sur}$ is in proportion to temperature for the outgoing thermal energy, as given by Supplementary Equation 3.

$\text{Q}\text{sur}\text{ = }\text{hS }\text{(}\text{T}-\text{T}\text{amb}\text{)}$ (3)

where *h* is heat transfer coefficient, *S* is the surface area of the container, and *T*_amb_ is ambient temperature of the surroundings.

According to Supplementary Equation 3, when the system temperature will reach a maximum, the heat input is equal to heat output:

$\text{Q}\text{NC}\text{+}\text{Q}\text{B}\text{= }\text{hS }\text{(}\text{T}\text{max}-\text{T}\text{amb}\text{)}$ (4)

where *T*_max_ is the equilibrium temperature. The 865 nm laser heat conversion efficiency ($\text{η}$) can be determined by substituting Supplementary Equation 2 for $\text{Q}\text{sur}$ into Supplementary Equation 4 and rearranging to get

$\text{η}\text{ }\text{= }\frac{\text{hS }\text{(}\text{T}\text{max }-\text{T}\text{amb}\text{)}\text{ }- \text{Q}\text{B}}{\text{I }\text{(1 }-\text{ }\text{10}^{{-A}_{\text{865}}}\text{) }}$ (5)

where ${\text{ }\text{Q}}_{\text{B}}$ was measured independently to be 8.5 mW, the (*T*_max_-*T*_amb_) was 13.2 °C according to **Supplementary Figure 10a**, *I* is 800 mW cm^-2^, *A*_865_ is the absorbance (1.074) of UCL-NCs at 865 nm (**Supplementary Figure 10c**). Here, *hS* is calculated by introducing *θ*, is defined as the expression below:

$\text{θ}\text{ }\text{=}\frac{\text{T }-\text{T}\text{amb}}{\text{T}\text{max }-\text{ }\text{T}\text{amb}}$ (6)

and a sample system time constant *τ_s_*

$\text{τ}_{\text{s}}\text{=}\frac{\text{∑}_{\text{i}}\text{m}_{\text{i}}\text{C}_{\text{p}\text{,i}}}{\text{hS}}$ (7)

which is substituted into Supplementary Equation 4 and rearranged to yield

$\frac{\text{d}\text{θ}}{\text{d}\text{t}}\text{=}\frac{\text{1}}{\text{τ}_{\text{s}}}\left[ \frac{\text{Q}_{\text{NC}}\text{+}\text{Q}_{\text{B}}}{\text{hS}\left( \text{T}_{\text{max}}-\text{T}_{\text{amb}} \right)}-\text{θ} \right]$ (8)

At the cooling stage of the aqueous dispersion of the UCL-NCs, the light source was shut off, the$\text{Q}_{\text{NC}}\text{+}\text{Q}_{\text{B}}\text{=}$0, reducing the Supplementary Equation 9

$\text{d}\text{t}\text{ }\text{=}-\text{τ}_{\text{s}}\frac{\text{d}\text{θ}}{\text{θ}}$ (9)

and integrating, giving the expression

$\text{t}\text{ }\text{=}-\text{τ}_{\text{s}}\text{ln}\text{θ}$ (10)

Therefore, time constant for heat transfer from the system is determined to be *τ_s_* = 213.9 s by applying the linear time data from the cooling period vs negative natural logarithm of *θ* **(Supplementary Figure 10b)**. In addition, the m is 1 g and the C is 4.2 J g^-1^. Thus, according to Supplementary Equation 7, the *hS* is deduced to be 19.7 mW °C^-1^. Substituting 19.7 mW °C^-1^ into the *hS* into Supplementary Equation 5, the 865 nm laser heat conversion efficiency (*η*) of UCL-NCs can be calculated to be 34.4 %.


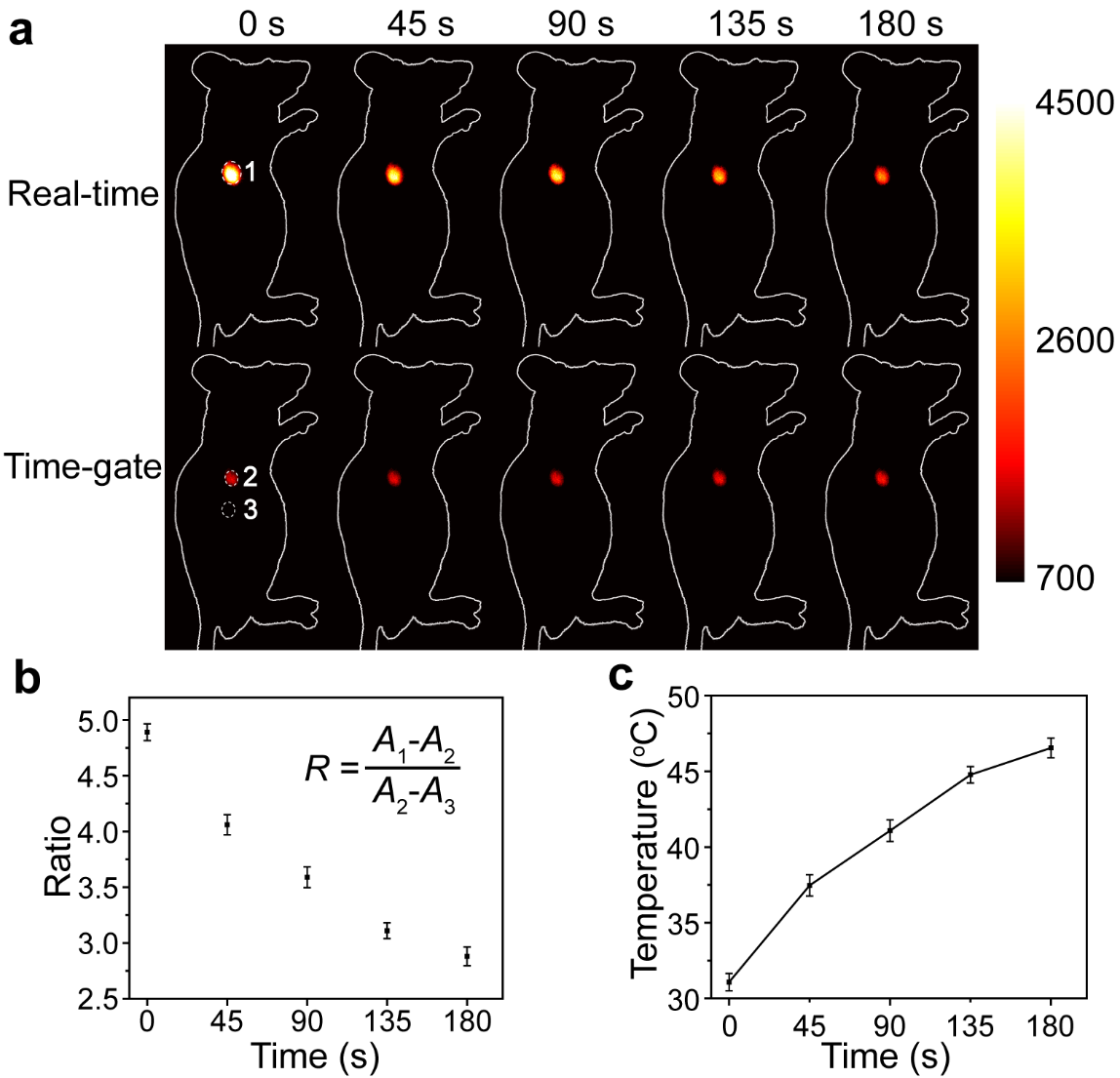


**Supplementary Figure 11.** Intratumoral temperature monitoring in vivo during the PTT. (a) Real-time and time-gate UCL images of nude mice with the injection of UCL-NCs at different time under the irradiation of an 865 nm laser (0.5 W cm^-2^). (b) Calculated ratio of *I*_PbS_ to *I*_Tm-UCNPs_ obtained from figure (a). (c) The intratumoral temperature at different time during the PTT. Error bars are defined as s.d. Source data are provided as a Source Data file.


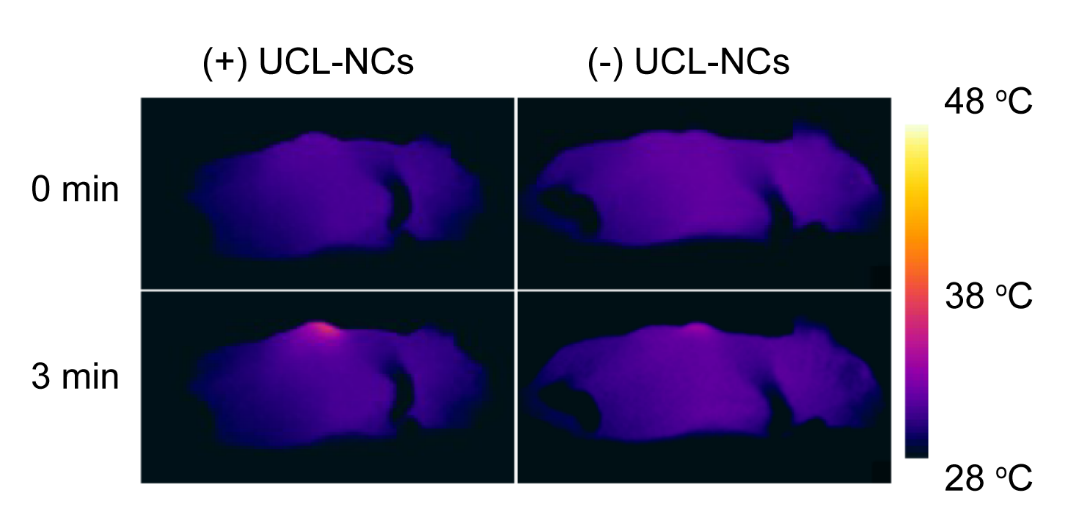


**Supplementary Figure 12.** Thermal images of nude mice with (left panel) and without (right panel) injection of UCL-NCs under the 865-nm irradiation (0.5 W cm^-2^).


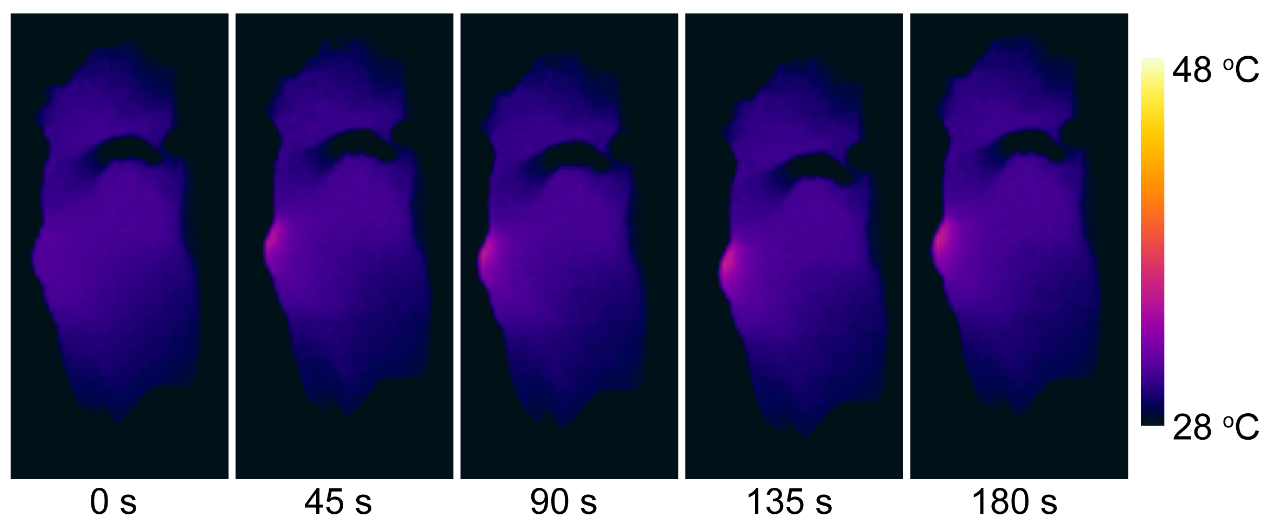


**Supplementary Figure 13**. Thermal images of nude mice with the injection of UCL-NCs at different time under the irradiation of an 865 nm laser (0.5 W cm^-2^).


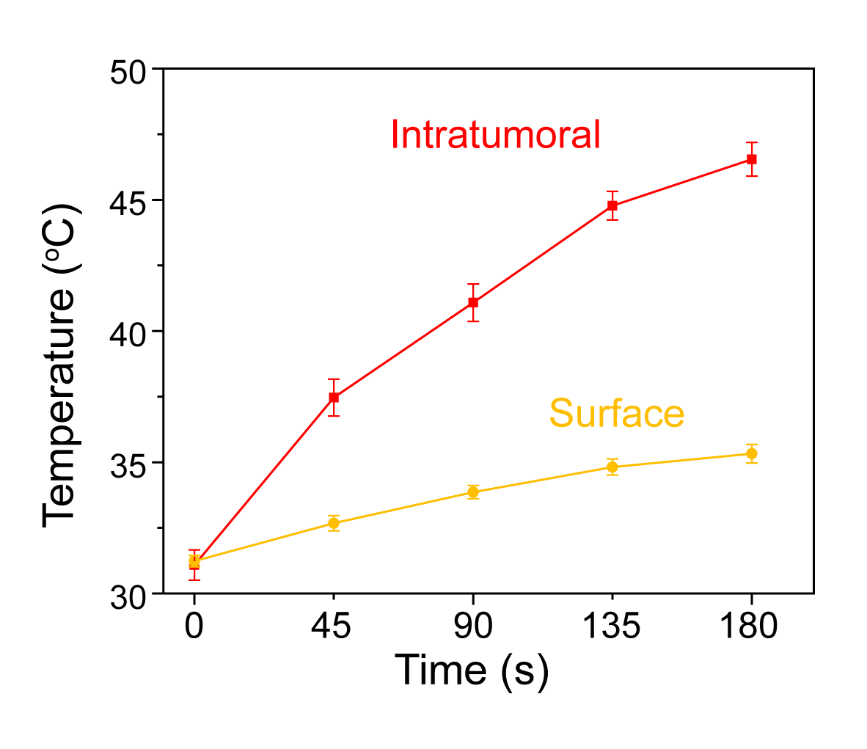


**Supplementary Figure 14.** Intratumoral and skin surficial temperature measured during the PPT of the tumor area. The data were collected using the home built time-gate imaging system and a thermographic camera, respectively. Average values of intratumoral and skin surficial temperature at different time points were given based on three times measurement. Error bars were defined as s.d. Source data are provided as a Source Data file.


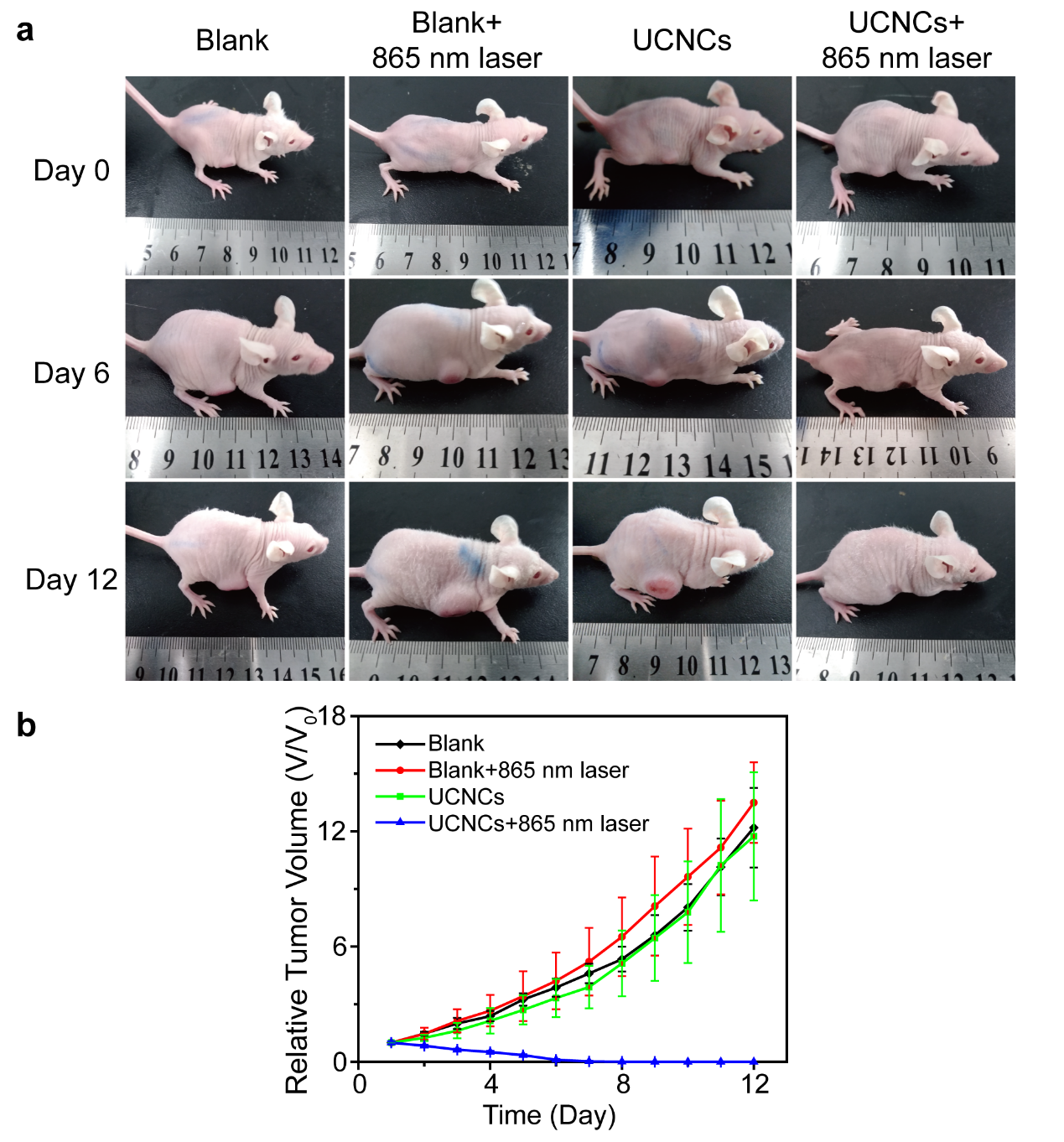


**Supplementary Figure 15.** Tumor size changes in different groups of S180 tumour-bearing nude mice. (a) Representative photos of tumor-bearing nude mice before and after treatment. (b) Relative tumor volume changes with time in different groups. The relative tumor volumes were normalized to their initial volumes before treatments. Average values of tumor volumes were calculated from five mice. Error bars were defined as standard deviation. Error bars are defined as s.d. Source data are provided as a Source Data file.


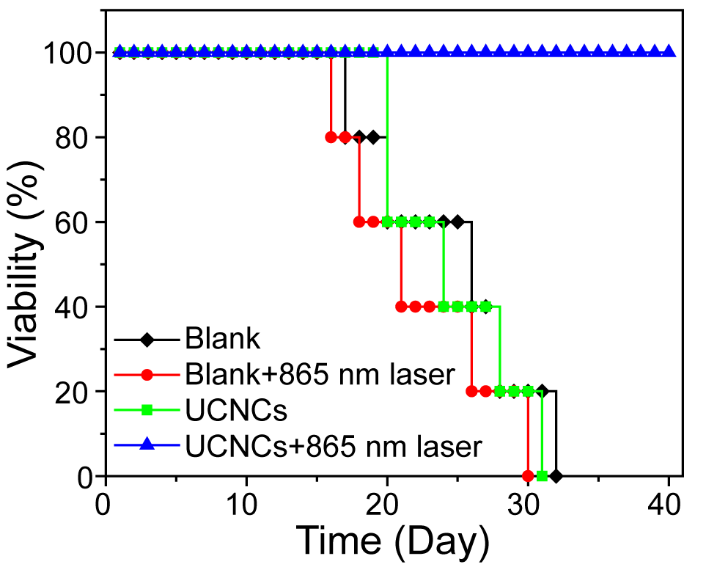


**Supplementary Figure 16.** Survival curves of mice of different groups with various treatments.

**Supplementary Note 3: Temperature feedback photothermal therapy in vivo.**

PbS can partially convert the absorbed optical energy into heat, which confers their tumor killing capabilities. To evaluate photothermal properties of UCL-NCs, the photothermal conversion efficiency, which is defined as the percentage of the absorbed energy transformed into heat, were measured under 865 nm laser irradiation (0.8 W cm^-2^) and calculated according to the method reported in literature. Detailed data are shown in **Supplementary Figure 10**. The photothermal conversion efficiency (*η*) of this UCL-NCs is 34.4% here, which is comparatively high to those widely applied PTT agents such as Cu_9_S_5_ (25.7%) and gold nanorods (21%).[^6^](#_ENREF_1) The photothermal conversion efficiency indicates that the UCL-NCs can be used as a PTT agent to obtain the therapeutic effect. Then the ability of UCL-NCs for UCL imaging guided PTT was tested on xenograft S180 tumor mouse via intratumoral injection. Two hours after the injection of UCL-NCs (2 mg mL^-1^, 50 μL), UCL of UCL-NCs in tumor region was collected during laser irradiation in order to calculated intratumoral temperature (**Supplementary Figure 11**). Meanwhile, surficial temperature was monitored via thermal camera during the treatment (**Supplementary Figures 12 and 13**). After being irradiated with an 865 nm laser (500 mW cm^-2^) for 3 min, intratumoral temperature shows a sharp increment and increases to as high as 47 ^o^C, which is high enough to produce tumor regression. In contrast to this, surficial temperature has only about 3 ^o^C increment **(Supplementary Figure 14**). This observation is consistent with previous reported works in literature. To investigate the in vivo therapeutic efficacy of UCL-NCs, tumor-bearing mice were randomly divided into four groups (n = 5) as follows: (a) Control (no treatment), (b) 865 nm laser only, (c) UCL-NCs only and (d) UCL-NCs + 865 nm laser (treatment). The tumour in each mouse of the group (b) and group (d) was irradiated with an 865 nm laser for 3 min every 24 h and lasted for five days. At the third day, the tumours in the treatment group appear shrinkage and are finally eliminated at the ninth day without any regrowth during forty days' watching. In contrast, neither laser irradiation (group (b)) nor UCL-NCs treatment (group (c)) affected tumour growth. The tumour sizes of each mouse in the reference groups (group (a, b and c)) show a rapid increment (**Supplementary Figure 15)**. The treatment group survived for over 40 days, while the average lifespan for tumour-bearing mice in reference groups was only about 24 days (**Supplementary Figure 16**).

**Supplementary Reference.**

Reference

1. del Rosal, B. *et al.* Infrared-Emitting QDs for Thermal Therapy with Real-Time Subcutaneous Temperature Feedback. *Adv. Funct. Mater.* **26**, 6060-6068 (2016).
2. Ceron, E.N. *et al.* Hybrid nanostructures for high-sensitivity luminescence nanothermometry in the second biological window. *Adv. Mater.* **27**, 4781-4787 (2015).
3. Xu, M. *et al.* Ratiometric nanothermometer in vivo based on triplet sensitized upconversion. *Nat. Commun.* **9**, 2698 (2018).
4. Zhu, X. *et al.* Temperature-feedback upconversion nanocomposite for accurate photothermal therapy at facile temperature. *Nat. Commun.* **7**, 10437 (2016).
5. Ximendes, E.C. *et al.* In Vivo Subcutaneous Thermal Video Recording by Supersensitive Infrared Nanothermometers. *Adv. Funct. Mater.* 1702249 (2017).
6. Huang, X.; Zhang, W.; Guan, G.; Song, G.; Zou, R.; Hu, J., Design and Functionalization of the NIR-Responsive Photothermal Semiconductor Nanomaterials for Cancer Theranostics. *Acc. Chem. Res.* **50**, 2529-2538 (2017).
