# Supplementary material for: Ratiometric upconversion nanothermometry with dual emission at the same wavelength decoded via a time-resolved technique
Source: Nat Commun. 2020 Jan 7;11:4. doi: 10.1038/s41467-019-13796-w (PMC6946702; doi:10.1038/s41467-019-13796-w)
Supplement: Supplementary file 2 — Peer Review File [file 41467_2019_13796_MOESM2_ESM.pdf]

Reviewers' comments:

Reviewer #1 (Remarks to the Author):

The work of Xiaochen Qiu et al. describes the fabrication of a ratiometric upconversion luminescence nanothermometry based on a hybrid structure formed by spherical clusters of upconversion PbS QDs and NaYbF4:0.5%Tm@NaYF4:10%Yb@NaYF4:50%Nd NPs embedded in a polyethylene glycol modified silica shell and its use to measure the temperature in vivo (on xenograft S180 tumor mouse) during photothermal experiments.

Temperature in-situ measurements during photothermal processes in vivo were already reported (see, for instance, refs. 24-26 of the paper) with relative thermal sensitivity values analogous to that reported here (for instance, 10.1002/adfm.201702249). The pros and cons of hybrid nanothermometers (refs. 31 and 34 of the manuscript, for instance) and heater-thermometer nanoplatforms (e.g., 10.1021/acsnano.5b00059) were already discussed and in vivo photothermal heating, tumor localization through luminescence, intratumoral thermal sensing and photothermal therapy of tumors were also demonstrated (references mentioned above and 10.1002/adom.201500726, as examples). The degree of novelty of the work is, thus, low, being the findings reported essentially incremental (even accordingly to the work done before by the authors). Although not entirely clear the principal novelty seems to be the way as the ratiometric signal is measured (the two upconversion emissions working at the same wavelength are decoded via a time-resolved technique). With that said, I have serious doubts about the significance of this work for Nature Communications, but the judgement about the mismatches between the degree of novelty of a certain paper and the journal standards is essentially an editor’s issue. In any case, I would like to encourage authors to consider the following points:

1) The advantages of the way of measuring the ratiometric signal relatively to what is reported before must be clearly stated.

2) The hybrid heater-thermometer platform is a complex structure. What is the ratio of PbS QDs and NaYbF4:0.5%Tm@NaYF4:10%Yb@NaYF4:50%Nd upconversion NPs (UCNPs) per cluster? Why the excitation is adjusted adding the Nd shell? Is that tuning dependent of the shell thickness? What is the role of the Yb-based buffer? What is the homogeneity of the distribution of QDs and UCNPs and how this determines the light-to-heat conversion efficiency?

3) The spectra presented in Figure 3 are measured in the isolated particles or in the clusters? Is there any difference between the thermometric response of the isolated particles relatively to what happens in the heater-thermometer clusters?

4) The calculus of IPbS is not explained (page 9).

5)

How the temperature uncertainty is calculated (a reference is missing)? The temperature dependence of the relative sensitivity and temperature uncertainty should be represented.

6) The reported photothermal conversation efficiency values are common (much smaller than those reported for other lanthanide-based NPs, 10.1039/c7nr02630g and 10.1002/adom.201500726).

Reviewer #2 (Remarks to the Author):

In the manuscript, a new strategy to fabricate a ratiometric upconversion luminescence nanothermometry is provided based on a hybrid structure composed of upconversion emissive PbS quantum dots and NaYbF4:0.5%Tm@NaYF4:10%Yb@NaYF4:50%Nd nanoparticles. The ratiometric signal composed of two upconversion emissions working at the same wavelength but different luminescent lifetimes are decoded via a time-resolved technique. Although it is illustrated to avoid the absorption and scattering of complicated tissue limitations in vivo, there have been existed many different types and various delicate-designs about the upconversion luminescence nanothermometry until now. This work lacks of originality and details are insufficient, and this manuscript should be published elsewhere

1. The nanothermometry here demonstrates a more precise temperature measurement in tissue by working with a time-resolved technique instead of the difference in wavelength in previous nanothermometry, could its sensor accuracy be further improved with both the two strategies combined?

2. It is revealed that both IPbS and ITm have a strong dependence on temperature from temperature dependent UCL spectra of PbS QDs, core-shell-shell Nd sensitized Tm-UCNPs, and the plots of IUCL versus T of PbS QDs and Tm-UCNPs. While, the nanothermometry works via a time-resolved technique, the corresponding plots calculated from time-resolved spectrometer may be more important.

3. The experiments in vivo without UCL-NCs also showed a significantly surficial temperature-increment, which is not that different from the treatment group only estimating from the thermal images, but induced an entirely different result. Details are insufficient.

4. With an approximate 8 nm-thick polyethylene glycol (PEG) modified silica shell, it demonstrated that the final UCL-NCs@SiO2 showed almost invisible increase and even shrink in size in TEM images Fig.2C and D.

5. The data provided in Fig. S10 indicates the photothermal stability is not perfect, and much more information about the stability (photothermal stability and structural stability) is needed.

Reviewer #3 (Remarks to the Author):

In this paper authors have developed a novel nanostructure for high sensitivity ratiometric thermal sensing. It is based on the combination of upconverting rare earth nanoparticles ad infrared emitting PbS QDs. The different quenching mechanisms of these structures makes possible thermal reading from a ratiometric approach. Instead of acquiring the ratiometric parameter from a simple analysis of the emission spectrum of their composites (that is the result of the superposition of the emission of UCNPs and PbS QDs) authors use time decoding to elucidate the relative contributions of these two structures. The thermal sensitivity obtained is quite good (5% per degree) and authors used this structures to perform very preliminary in vivo experiments.

The structure here reported is interesting but I can not see the clear contribution of this work to the state of the field. I think authors should discuss with more detail the advantajes of their approach in respect to what is already published on the field. For instance:

1.- It is not clear why time-decoding is beneficial in respect to a simple spectroscopic analysis. The thermal sensitivity curves should be given as obtained by applying time decoding and simple spectral anlysis if CW emission spectra.

2.- The brightness of the here used nanostructures have to be given. What is the minimum excitation density that can be used to obtaine subtissue thermal sensing. How is related the thermal accuracy with the illumination intensity?

3.- In the experimental data included in Figure 4e the time resolution seems to be of tens of seconds. Why is that? Is there any limitation not discussed that avoids real time thermal sensing based on this sensing system?

4.- What is exactly the heating source in their in vivo experiments? If it is associated to the heating efficiency of their nanostructures then this should be described and evaluated.

5.- What is the actual penetration depth of their structures?

Responses to the Reviewers' Reports **Manuscript (**NCOMMS-19-08975)

Reviewer #1:

Comments:

The work of Xiaochen Qiu et al. describes the fabrication of a ratiometric upconversion luminescence nanothermometry based on a hybrid structure formed by spherical clusters of upconversion PbS QDs and NaYbF4:0.5%Tm@NaYF4:10%Yb@NaYF4:50%Nd NPs embedded in a polyethylene glycol modified silica shell and its use to measure the temperature in vivo (on xenograft S180 tumor mouse) during photothermal experiments.

Temperature in-situ measurements during photothermal processes in vivo were already reported (see, for instance, refs. 24-26 of the paper) with relative thermal sensitivity values analogous to that reported here (for instance, 10.1002/adfm.201702249). The pros and cons of hybrid nanothermometers (refs. 31 and 34 of the manuscript, for instance) and heater-thermometer nanoplatforms (e.g., 10.1021/acsnano.5b00059) were already discussed and in vivo photothermal heating, tumor localization through luminescence, intratumoral thermal sensing and photothermal therapy of tumors were also demonstrated (references mentioned above and 10.1002/adom.201500726, as examples). The degree of novelty of the work is, thus, low, being the findings reported essentially incremental (even accordingly to the work done before by the authors). Although not entirely clear the principal novelty seems to be the way as the ratiometric signal is measured (the two upconversion emissions working at the same wavelength are decoded via a time-resolved technique). With that said, I have serious doubts about the significance of this work for Nature Communications, but the judgement about the mismatches between the degree of novelty of a certain paper and the journal standards is essentially an editor's issue. In any case, I would like to encourage authors to consider the following points:

Response: We appreciate the valuable comments. In fact, the main point shown in this manuscript is the accurate detection of temperature in vivo. This is a challenge because the complex biological tissues will affect the optical signals significantly. Thus the ratiometric detection mode is preferred, and the same working wavelength was used for the two emission signal channels to maintain the same influence of

tissues. In the last manuscript we have not provide enough data to indicate this point. In this re-submitted manuscript, we have compared this temperature sensing method to other detection modes to show the superiorities. We believe this will provide a new method to obtain accurate temperature especially for the in vivo applications.

Comment 1. *The advantages of the way of measuring the ratiometric signal relatively to what is reported before must be clearly stated.*

Response: We appreciate this valuable advice. In order to clearly stated the advantages of the way of measuring the ratiometric signal relatively to what is reported before, in our revised manuscript, control temperature monitoring experiments of conventional and this same wavelength ratiometric nanothermometry are performed in details using pork covering to simulate the situation *in vivo*. (Fig. 3 and 4). As shown in those results, high accuracy of this same wavelength ratiometric nanothermometry *in vivo* are marked exhibited, owing to eliminating errors caused from different wavelength ratio. In detail, for conventional ratiometric nanothermometry, the temperature uncertainty is 5.6 K which makes it unpractical to monitor the precise temperature in vivo. In contrast to this, for the same wavelength ratiometric nanothermometry, a temperature uncertainty of 0.3 K is obtained owing to eliminating the errors from the effect of heterogeneity on different wavelengths, which exhibits an overwhelming advantage in temperature monitoring in vivo. Moreover, thermal sensitivity of this unique ratiometric nanothermometer is up to 5.6% K^{-1}, which is much higher than other conventional upconversion luminescence nanothermometers whose thermal sensitivity is about 1%. Finally, our manuscript exhibits the first example to fabricate an NIR upconversion luminescence ratiometric nanothermometer (See in Table 1) for monitoring temperature in biological system.

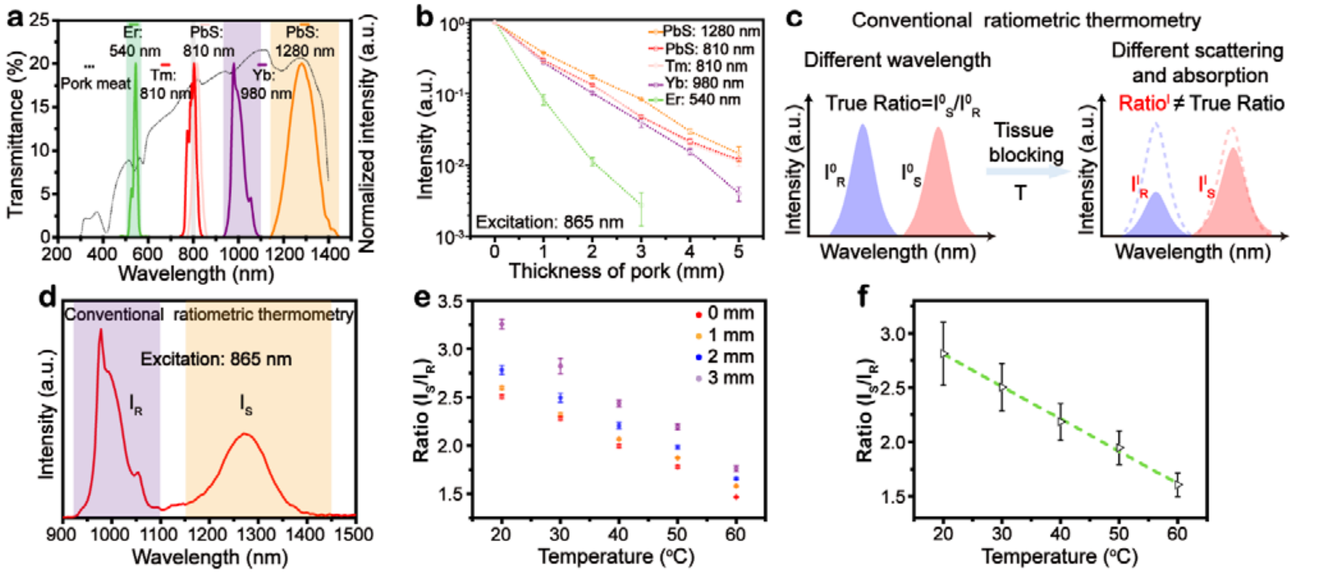

Fig. 3 Errors derived from the tissue blocking in conventional luminescent ratiometric thermometry. (a) Transmittance spectrum (left axis) of 1 mm pork and emission spectra (right axis) of Er-UCNPs, Tm-nanoparticles and PbS QDs. Colour shades correspond to the spectral ranges for collecting respective emissions. (b) Normalized intensities obtained from Er-UCNPs, Tm-nanoparticles and PbS QDs with respect to thickness of pork under the excitation of 865 nm laser. Each data and error bar represents mean ± s.d. based on three times measurements on spectrometer. (c) Schematic illustration of conventional luminescent ratiometric thermometry with different wavelength emission. (d) Stokes emission spectrum of UCL-NCs under the excitation of 865 nm laser. I_R and I_S are the reference emission (Yb^{3+} emission, 980 nm) and temperature sensitive emission (PbS emission, 1280 nm), respectively. (e) Ratios of I_S to I_R at different temperature with various thickness pork blocking. Each data and error bar represents mean ± s.d. based on three times measurements on spectrometer. (f) A plot of I_S/I_R versus T to calibrate the temperature. Average values of I_S/I_R under different temperature were obtained from the ratios with different thickness (0, 1, 2 and 3 mm) pork blocking. Error bars were defined as s.d.

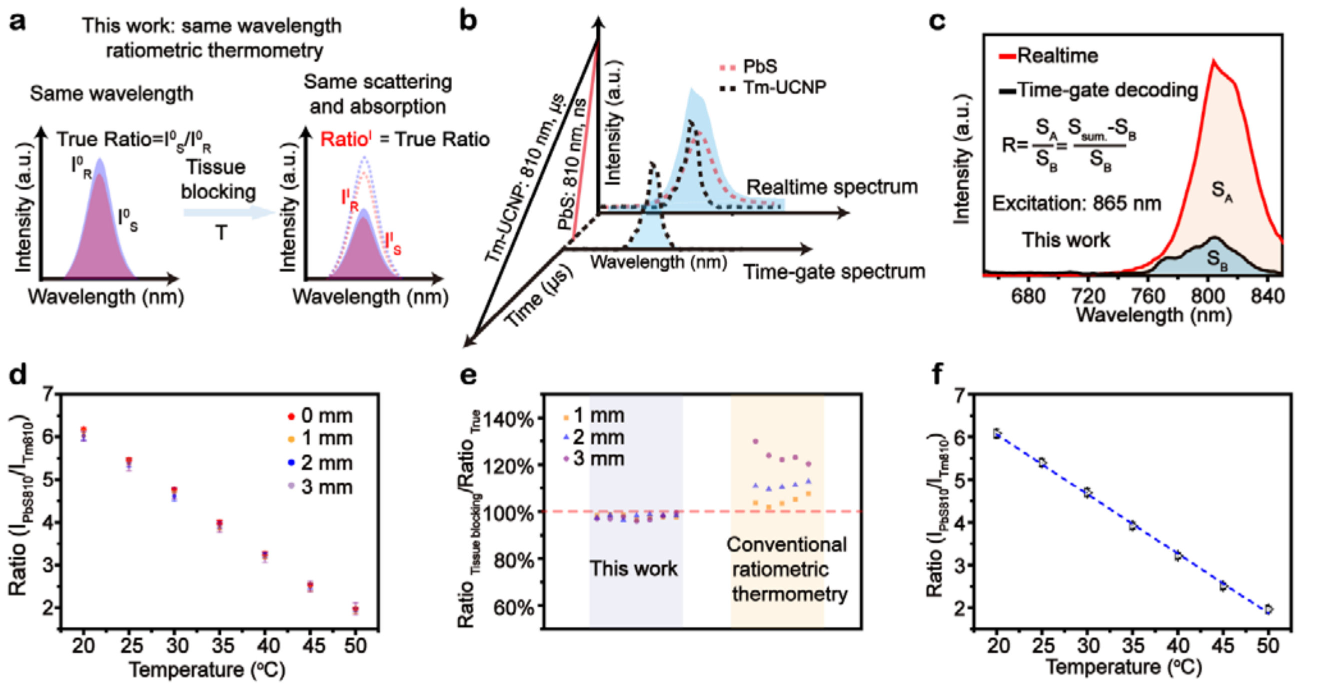

Fig. 4 Temperature monitoring ability evaluation of the same wavelength ratiometric thermometry based on the UCL emission of UCL-NCs. (a) Schematic illustration of conventional ratiometric thermometry with the same wavelength emission. (b) Schematic diagram of UCL signals separation via time-resolved decoding. (c) UCL emssion signals decoding of UCL-NCs using time-resolved spectrometer. (d) The same wavelength ratio of I_{PbS} to I_{Tm} obtained time-resolved spectrometer at different temperature with various thickness pork blocking. Each data and error bar represents mean ± s.d. based on three times measurements with an excitation of 865 nm laser on spectrometer. (e) Ratio values deviation with different thickness pork blocking to ratio signals measured without pork blocking using the same wavelength or conventional ratiometric thermometry. (f) A plot of I_{PbS810}/I_{Tm810} versus T to calibrate the temperature. Average values of I_{PbS810}/I_{Tm810} under different temperature were given to fit the calibration curve based on the spectra with different thickness pork blocking. Error bars were defined as s.d.

Table 1. Experimental parameters of several typical in vivo nanothermometers. $\lambda_{Ex.}$, $\lambda_{Em.}$, √, × and S_R correspond to excitation wavelength, emission wavelength, yes, no and relative thermal sensitivity, respectively.

Material	$\lambda_{Ex.}$ (nm)	$\lambda_{Em.}$ (nm)	NIR Ex. and Em.	Ratio	Same Em.	S_R (10^{-2} K^{-1})
PbS/CdS/ZnS QDs[26]	808	1270	√	×	-	1.0
PbS/CdS/ZnS-Nd NPs[34]	808	1060 and 1250	√	√	×	2.5
TTA-Nd-NPs[31]	635 and 808	540 and 1060	×	√	×	7.1
CsUCNP @C[24]	980	525 and 545	×	√	×	1.1
Er-Yb@Yb-Tm LaF_3[37]	690	1000, 1230 and 1550	×	√	×	5.0
UCL-NCs[This work]	865	810 and 810	√	√	√	5.6

Comment 2. *The hybrid heater-thermometer platform is a complex structure. (a) What is the ratio of PbS QDs and NaYbF4:0.5%Tm@NaYF4:10%Yb@NaYF4:50%Nd upconversion NPs (UCNPs) per cluster? (b) Why the excitation is adjusted adding the Nd shell? (c) Is that tuning dependent of the shell thickness? (d) What is the role of the Yb-based buffer? (e) What is the homogeneity of the distribution of QDs and UCNPs and how this determines the light-to-heat conversion efficiency?*

We appreciate this valuable advice. (a) The mass ratio of PbS QDs to UCNPs is 4:1 according to the materials adding in our synthesis procedure. (b) Typically, UCNPs are simultaneously doped with sensitizers (usually Yb^{3+} or Nd^{3+}) and activators (Er^{3+}, Tm^{3+}, or Ho^{3+} in most cases). The sensitizers absorb near-infrared (NIR) photons and then transfer the energy to the activators. Yb^{3+} has only one excited state ($^2F_{5/2}$), which results in the absorption band located at *ca.* 980 nm (from $^2F_{7/2}$ to $^2F_{5/2}$). However, for upconversion emissive PbS QDs, the thermal sensitivity is poor under the excitation with a 980 nm laser. In contrast to this, when exciting with an 865 nm laser, a high thermal sensitivity and good linearity are obtained between UCL intensity and temperature. So, Nd shell was added to absorb 865 nm photons owing to the matching energy level difference between $^4F_{3/2}$ and $^4I_{9/2}$. (c) The tuning is not dependent on the shell thickness. The excitation wavelength is determined by the intrinsic energy level of Nd^{3+}, which will not change with the thickness of Nd shell. (d) The Yb-based buffer between core and outer shell serves as a shield to prevent quenching interactions between the activator (Tm^{3+}) and sensitizer (Nd^{3+}). (e) QDs and UCNPs are distributed in cluster randomly and the light-to-heat conversion efficiency was calculated in Supporting Information Section 5 as following:

SI-5 Calculation of Photothermal Conversion Efficiency.

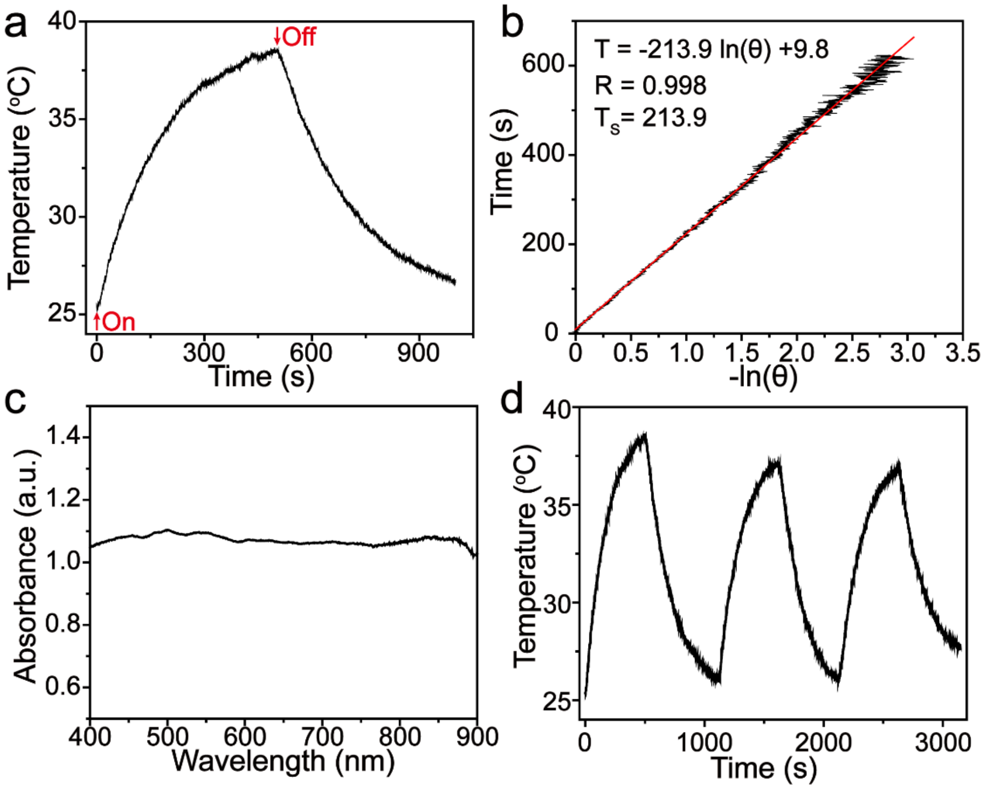

Figure S9. Characterization of photo thermal properties of UCL-NCs. (a) Temperature rising and cooling curve of UCL-NCs aqueous dispersion under irradiation of an 865 nm laser (0.8 W/cm^2). During the process, the irradiation lasted for 500 s, and then the laser was shut down. (b) Determination of the time constant for heat transfer from the cooling profile in Figure (a). (c) UV-NIR absorption spectrum of UCL-NCs aqueous dispersion. (d) Heat effect of UCL-NCs aqueous solution over 3 cycles of continuous re-irradiation (0.8 W/cm^2).

Disscussion. According to the method described in the literature, the total energy conservation for the system can be expressed by Supplementary Equation 1.

$$\sum_i m_i C_{p,i} \frac{dT}{dt} = Q_{NC} + Q_B - Qsur \quad (1)$$

where m and C_p are the mass and heat capacity of water respectively, T is the solution temperature, Q_{NC} is the energy induced by UCL-NCs, Q_B is the baseline energy induced by the sample cell, and $Qsur$ is heat conduction away from the surface by air. Q_{NC} is caused by non-radiative transition of PbS QDs under irradiation of 865 nm laser:

$$Q_{NC} = I(1 - 10^{-A_{865}})\eta \quad (2)$$

where I is the laser power, η is the conversion efficiency from incident laser energy to thermal energy, and A_{865} is the absorbance of UCL-NCs at wavelength of 865 nm. On the other hand, Q_B, expressing heat dissipated from light absorbed by the sample cell, was measured independently to be 8.5 mW using a quartz cuvette containing pure water without UCL-NCs. Moreover, Q_{sur} is in proportion to temperature for the outgoing thermal energy, as given by Supplementary Equation 3.

$$Q_{sur} = hS(T - T_{\mathrm{amb}}) \qquad (3)$$

where h is heat transfer coefficient, S is the surface area of the container, and T_{amb} is ambient temperature of the surroundings.

According to Supplementary Equation 3, when the system temperature will reach a maximum, the heat input is equal to heat output:

$$Q_{NC} + Q_B = hS(T_{max} - T_{amb}) \qquad (4)$$

where T_{max} is the equilibrium temperature. The 865 nm laser heat conversion efficiency (η) can be determined by substituting Supplementary Equation 2 for Q_{NC} into Supplementary Equation 4 and rearranging to get

$$\eta = \frac{hS(T_{max} - T_{amb}) - Q_B}{I(1 - 10^{-A_{865}})} \qquad (5)$$

where Q_B was measured independently to be 8.5 mW, the (T_{max}-T_{amb}) was 13.2 °C according to Figure S9a, I is 800 mW cm^{-2}, A_{865} is the absorbance (1.074) of UCL-NCs at 865 nm (Figure S9c). Here, hS is calculated by introducing θ, is defined as the expression below:

$$\theta = \frac{T - T_{amb}}{T_{max} - T_{amb}} \qquad (6)$$

and a sample system time constant τ_s

$$\tau_s = \frac{\sum_i m_i C_{p,i}}{hs} \qquad (7)$$

which is substituted into Supplementary Equation 4 and rearranged to yield

$$\frac{d\theta}{dt} = \frac{1}{\tau_s}\left[\frac{Q_{cs} + Q_B}{hS(T_{max} - T_{amb})} - \theta\right] \qquad (8)$$

At the cooling stage of the aqueous dispersion of the UCL-NCs, the light source was shut off, the $Q_{NC} + Q_B = 0$, reducing the Supplementary Equation 9

$$dt = -\tau_s \frac{d\theta}{\theta} \qquad (9)$$

and integrating, giving the expression

$$t = -\tau_s \ln\theta \qquad (10)$$

Therefore, time constant for heat transfer from the system is determined to be τs = 213.9 s by applying the linear time data from the cooling period vs negative natural logarithm of θ (Figure S9b). In addition, the m is 1 g and the C is 4.2 J g^{-1}. Thus, according to Supplementary Equation 7, the hS is deduced to be 19.7 mW °C^{-1}. Substituting 19.7 mW °C^{-1} into the hS into Supplementary Equation 5, the 865 nm laser heat conversion efficiency (η) of UCL-NCs can be calculated to be 34.4 %.

Comment 3. *The spectra presented in Figure 3 are measured in the isolated particles or in the clusters? Is there any difference between the thermometric response of the isolated particles relatively to what happens in the heater-thermometer clusters?*

We appreciate this valuable advice. Both in our previous and revised manuscript, temperature calibration curves are measured using the clusters using time-gate technique. From our results, both isolated PbS QDs and UCL-NCs have a good thermal sensitivity.

Comment 4. *The calculus of IPbS is not explained (page 9).*

We appreciate this valuable advice. $I_{real\text{-}time}$ of UCL-NCs contains both UCL emissions of UCNPs (long lifetime) and PbS QDs (short lifetime). Then I_{UCNPs} was obtained using a 20 µs time-gate to eliminate the short lifetime signal of PbS QDs. So I_{PbS} was calculated from $I_{real\text{-}time}$ minus $I_{time\text{-}resolved}$.

Comment 5. *How the temperature uncertainty is calculated (a reference is missing)? The temperature dependence of the relative sensitivity and temperature uncertainty should be represented.*

We appreciate this valuable advice. Temperature uncertainty is calculated as following:

$$\sigma T = \frac{\sigma R}{S_R}$$

$$S_R = \frac{dRatio}{dT}\frac{1}{Ratio}$$

where *dRatio*/*dT* can get from the slope of temperature calibration curves. *σR* is the standard deviation of Ratio obatined from three times measurements. The thermal sensitivity and temperature uncertainty of this thermometer at 45 ^{o}C is about 5.6 % K^{-1} and 0.3 K.

Reference: C.D.S. Brites, A. Millan, L.D. Carlos, J.C.G. Bünzli, V.K. Pecharsky (Eds.), Handbook of the Physics and Chemistry of Rare Earths, Lanthanides in Luminescent Thermometry, vol. 49, Elsevier B.V., The Netherlands, 2016, pp. 339–427 (Chapter281).

Comment 6. *The reported photothermal conversation efficiency values are common (much smaller than those reported for other lanthanide-based NPs, 10.1039/c7nr02630g and 10.1002/adom.201500726).*

We appreciate this valuable advice. The photothermal conversation efficiency values reported in our manuscript are common. However, the main bright spot of our manuscript is the high accuracy of this same wavelength ratiometric nanothermometry in vivo. The photothermal ability was just used to bring in a temperature change *in vivo*.

Reviewer #2:

Comments:

In the manuscript, a new strategy to fabricate a ratiometric upconversion luminescence nanothermometry is provided based on a hybrid structure composed of upconversion emissive PbS quantum dots and NaYbF4:0.5%Tm@NaYF4:10%Yb@NaYF4:50%Nd nanoparticles. The ratiometric signal composed of two upconversion emissions working at the same wavelength but different luminescent lifetimes are decoded via a time-resolved technique. Although it is illustrated to avoid the absorption and scattering of complicated tissue limitations in vivo, there have been existed many different types and various delicate-

designs about the upconversion luminescence nanothermometry until now. This work lacks of originality and details are insufficient, and this manuscript should be published elsewhere.

Response: We appreciate the valuable comments. In fact, the main point shown in this manuscript is the accurate detection of temperature in vivo. This is a challenge because the complex biological tissues will affect the optical signals significantly. Thus the ratiometric detection mode is preferred, and the same working wavelength was used for the two emission signal channels to maintain the same influence of tissues. In the last manuscript we have not provide enough data to indicate this point. In this re-submitted manuscript, we have compared this temperature sensing method to other detection modes to show the superiorities. We believe this will provide a new method to obtain accurate temperature especially for the in vivo applications.

Comment 1. *The nanothermometry here demonstrates a more precise temperature measurement in tissue by working with a time-resolved technique instead of the difference in wavelength in previous nanothermometry, could its sensor accuracy be further improved with both the two strategies combined?*

Response: Thank you for your careful work. The basic effects of accurate microenvironment temperature monitor *in vivo* are shown in the formula as following:

$$I_{in\ vivo} = \mathrm{f}(p) \cdot \mathrm{f}(c) \cdot \mathrm{f}(a) \cdot \mathrm{f}(s) \cdot \mathrm{f}(x)$$

In the formula, f(p), f(c), f(a), f(s) and f(x) correspond to excitation power density, material concentration *in vivo*, light absorption, light scattering and other influence factors. The dual emission ratio luminescent nanothermometers can eliminate the influence of f(p) and f(c), owing to its self-referencing capability. However, f(a) and f(s) which depend strongly on wavelength still cannot be eliminated owing to the great difference in wavelength between the ratiometric emissions. In order to eliminate f(a) and f(s), in our manuscript, the same wavelength NIR emission ratio upconversion luminescent nanothermometry is fabricated and explored *in vivo* via time-resolved decoding.

Comment 2. *It is revealed that both IPbS and ITm have a strong dependence on temperature from temperature dependent UCL spectra of PbS QDs, core-shell-shell Nd sensitized Tm-UCNPs, and the plots of IUCL versus T of PbS QDs and Tm-UCNPs. While, the nanothermometry works via a time-resolved technique, the corresponding plots calculated from time-resolved spectrometer may be more important.*

Response: We appreciate this valuable advice. As shown in Fig. 4b and Fig. 4c, $I_{real\text{-}time}$ of UCL-NCs contains both UCL emissions of UCNPs (long lifetime) and PbS QDs (short lifetime). Then I_{UCNPs} was obtained using a 20 μs time-gate to eliminate the short lifetime signal of PbS QDs. So I_{PbS} was calculated from $I_{real\text{-}time}$ minus $I_{time\text{-}resolved}$. And the corresponding plots calculated from time-resolved spectrometer are shown in Figure 4d.

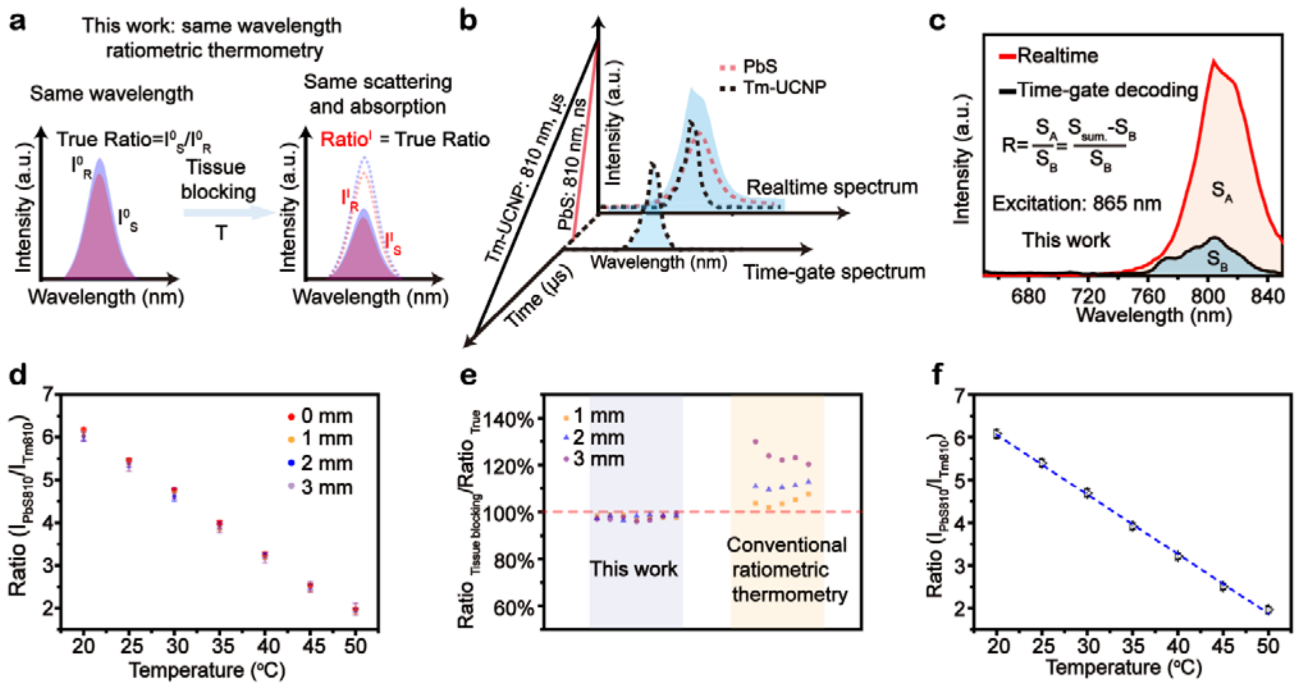

Fig. 4 Temperature monitoring ability evaluation of the same wavelength ratiometric thermometry based on the UCL emission of UCL-NCs. (a) Schematic illustration of conventional ratiometric thermometry with the same wavelength emission. (b) Schematic diagram of UCL signals separation via time-resolved decoding. (c) UCL emssion signals decoding of UCL-NCs using time-resolved spectrometer. (d) The same wavelength ratio of I_{PbS} to I_{Tm} obtained time-resolved spectrometer at different temperature with various thickness pork blocking. Each data and error bar represents mean ± s.d. based on three times measurements with an excitation of 865 nm laser on spectrometer. (e) Ratio values deviation with different

thickness pork blocking to ratio signals measured without pork blocking using the same wavelength or conventional ratiometric thermometry. (f) A plot of I_{PbS810}/I_{Tm810} versus T to calibrate the temperature. Average values of I_{PbS810}/I_{Tm810} under different temperature were given to fit the calibration curve based on the spectra with different thickness pork blocking. Error bars were defined as s.d.

Comment 3. *The experiments in vivo without UCL-NCs also showed a significantly surficial temperature-increment, which is not that different from the treatment group only estimating from the thermal images, but induced an entirely different result. Details are insufficient.*

Response: We appreciate this valuable advice. As shown in Figure S11, both the nude mice with and without injection of UCL-NCs show a little skin surficial temperature increasement. For the experiment group, about 3 °C skin surficial temperature increasement is observed, and for the control group, skin surficial temperature increasement is about 1 °C. In contrast to this, intratumoral temperature shows a sharp increment and increases to as high as 47 °C (Figure S13), which is high enough to produce tumor regression.

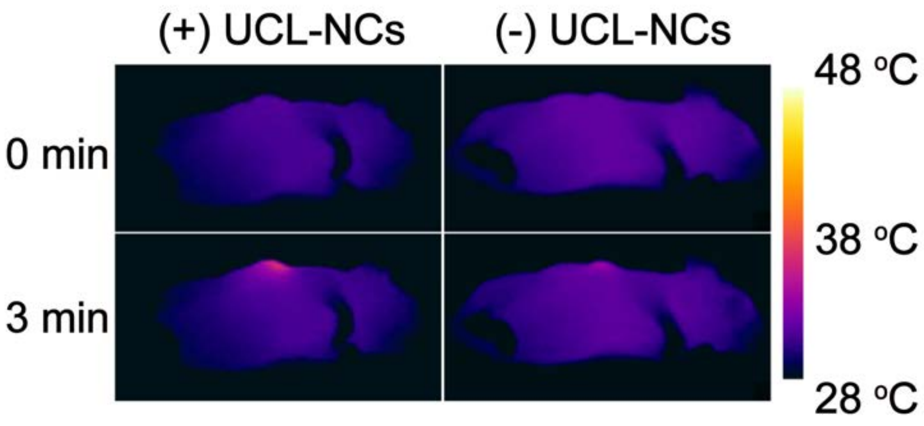

Figure S11. Thermal images of nude mice with (left panel) and without (right panel) injection of UCL-NCs under the 865-nm irradiation (0.5 W/cm^2).

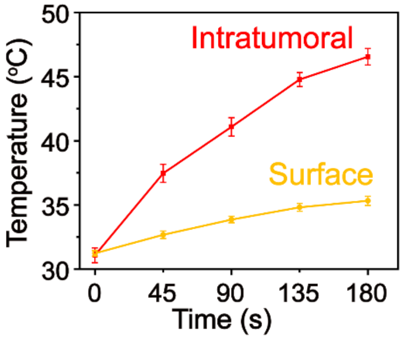

Figure S13. Intratumoral and skin surficial temperature measured during the PPT of the tumor area. The data were collected using the home built time-gate imaging system and a thermographic camera, respectively. Average values of intratumoral and skin surficial temperature at different time points were given based on three times measurement. Error bars were defined as s.d.

Comment 4. *With an approximate 8 nm-thick polyethylene glycol (PEG) modified silica shell, it demonstrated that the final UCL-NCs@SiO2 showed almost invisible increase and even shrink in size in TEM images Fig.2C and D.*

Response: We appreciate this valuable advice. The TEM images only demonstrate the sized of part nanoparticles. It is common that one or more nanoparticles have a smaller size. As shown in Fig. R1, UCL-NCs@SiO_2 shows about 20 nm size increment.

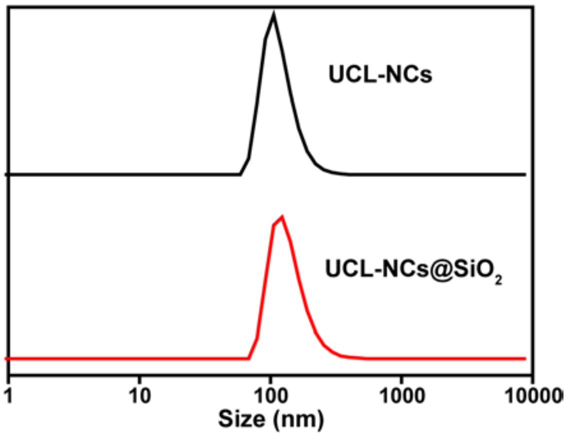

Figure R1. Dynamic light scattering data of UCL-NCs and UCL-NCs@SiO_2.

Comment 5. *The data provided in Fig. S10 indicates the photothermal stability is not perfect, and much more information about the stability (photothermal stability and structural stability) is needed.*

Response: We appreciate this valuable advice. The photothermal stability and structural stability are evaluated in continuous warming-cooling cycles. The stable ratiometric signals and hydrodynamic sizes all indicate the good staility of this structure.

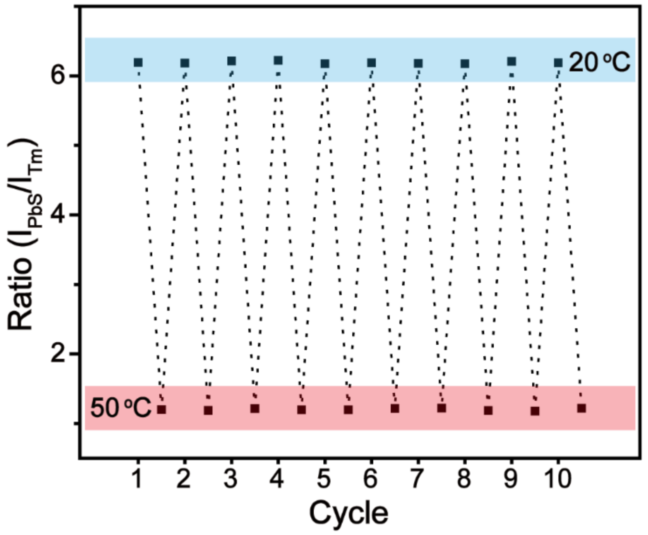

Figure S7. The reversibility of UCL-NCs tested in continuous warming-cooling cycles. Ratiometric signals were measured at 20 °C and 50 °C using a time gate spectrometer, respectively.

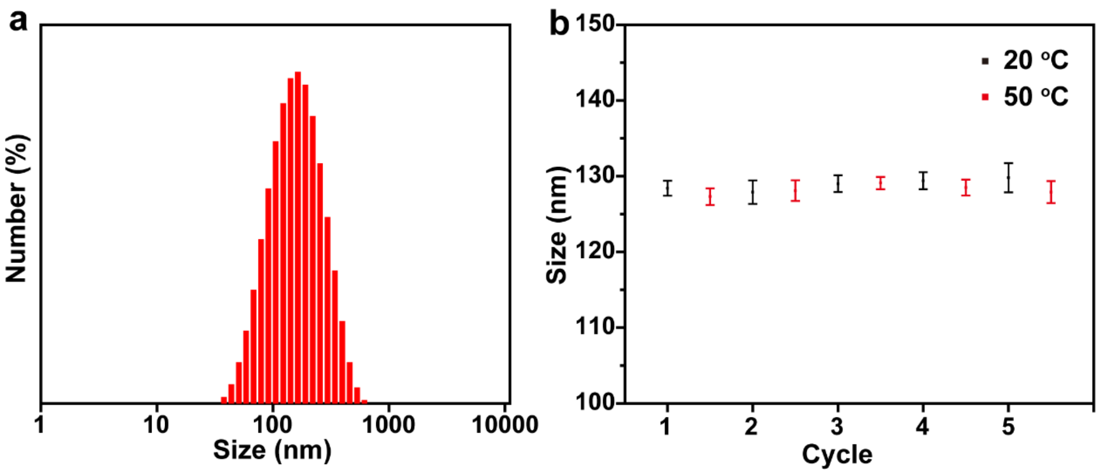

Figure S8. Heat stability of UCL-NCs evaluation. (a) The hydrodynamic sizes distribution of UCL-NCs measured by DLS analysis. (b) The hydrodynamic size of UCL-NCs measured at 20 or 50 ^{o}C in continuous warming-cooling cycles.

Reviewer #3:

Comments:

In this paper authors have developed a novel nanostructure for high sensitivity ratiometric thermal sensing. It is based on the combination of upconverting rare earth nanoparticles ad infrared emitting PbS QDs. The different quenching mechanisms of these structures makes possible thermal reading from a ratiometric approach. Instead of acquiring the ratiometric parameter from a simple analysis of the emission spectrum of their composites (that is the result of the superposition of the emission of UCNPs and PbS QDs) authors use time decoding to elucidate the relative contributions of these two structures. The thermal sensitivity obtained is quite good (5% per degree) and authors used this structures to perform very preliminary in vivo experiments.

The structure here reported is interesting but I can not see the clear contribution of this work to the state of the field. I think authors should discuss with more detail the advantajes of their approach in respect to what is already published on the field.

Response: We appreciate the valuable comments. In fact, the main point shown in this manuscript is the accurate detection of temperature in vivo. This is a challenge because the complex biological tissues will affect the optical signals significantly. Thus the ratiometric detection mode is preferred, and the same working wavelength was used for the two emission signal channels to maintain the same influence of tissues. In the last manuscript we have not provide enough data to indicate this point. In this re-submitted manuscript, we have compared this temperature sensing method to other detection modes to show the superiorities. We believe this will provide a new method to obtain accurate temperature especially for the in vivo applications.

For instance:

Comment 1. *It is not clear why time-decoding is beneficial in respect to a simple spectroscopic analysis. The thermal sensitivity curves should be given as obtained by applying time decoding and simple spectral anlysis if CW emission spectra.*

Response: We appreciate this valuable advice. In order to clearly stated the advantages of the way of measuring the ratiometric signal relatively to what is reported before, in our revised manuscript, control temperature monitoring experiments of conventional and this same wavelength ratiometric nanothermometry are performed in details using pork covering to simulate the situation *in vivo*. (Fig. 3 and 4). As shown in those results, high accuracy of this same wavelength ratiometric nanothermometry *in vivo* are marked exhibited, owing to eliminating errors caused from different wavelength ratio. In detail, for conventional ratiometric nanothermometry, the temperature uncertainty is 5.6 K which makes it unpractical to monitor the precise temperature in vivo. In contrast to this, for the same wavelength ratiometric nanothermometry, a temperature uncertainty of 0.3 K is obtained owing to eliminating the errors from the effect of heterogeneity on different wavelengths, which exhibits an overwhelming advantage in temperature monitoring in vivo.

As shown in Fig. 4b and Fig. 4c, $I_{real\text{-}time}$ of UCL-NCs contains both UCL emissions of UCNPs (long lifetime) and PbS QDs (short lifetime). Then I_{UCNPs} was obtained using a 20 μs time-gate to eliminate the short lifetime signal of PbS QDs. So I_{PbS} was calculated from $I_{real\text{-}time}$ minus $I_{time\text{-}resolved}$. And the corresponding plots calculated from time-resolved spectrometer are shown in Figure 4d.

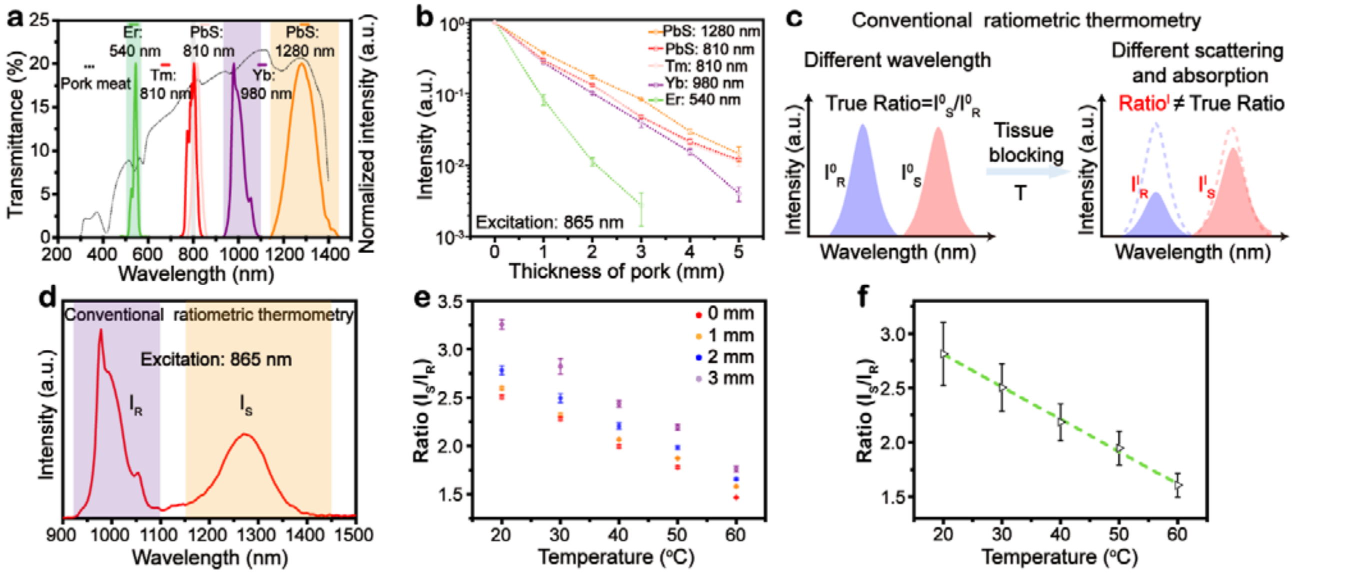

Fig. 3 Errors derived from the tissue blocking in conventional luminescent ratiometric thermometry. (a) Transmittance spectrum (left axis) of 1 mm pork and emission spectra (right axis) of Er-UCNPs, Tm-nanoparticles and PbS QDs. Colour shades correspond to the spectral ranges for collecting respective

emissions. (b) Normalized intensities obtained from Er-UCNPs, Tm-nanoparticles and PbS QDs with respect to thickness of pork under the excitation of 865 nm laser. Each data and error bar represents mean ± s.d. based on three times measurements on spectrometer. (c) Schematic illustration of conventional luminescent ratiometric thermometry with different wavelength emission. (d) Stokes emission spectrum of UCL-NCs under the excitation of 865 nm laser. I_R and I_S are the reference emission (Yb^{3+} emission, 980 nm) and temperature sensitive emission (PbS emission, 1280 nm), respectively. (e) Ratios of I_S to I_R at different temperature with various thickness pork blocking. Each data and error bar represents mean ± s.d. based on three times measurements on spectrometer. (f) A plot of I_S/I_R versus T to calibrate the temperature. Average values of I_S/I_R under different temperature were obtained from the ratios with different thickness (0, 1, 2 and 3 mm) pork blocking. Error bars were defined as s.d.

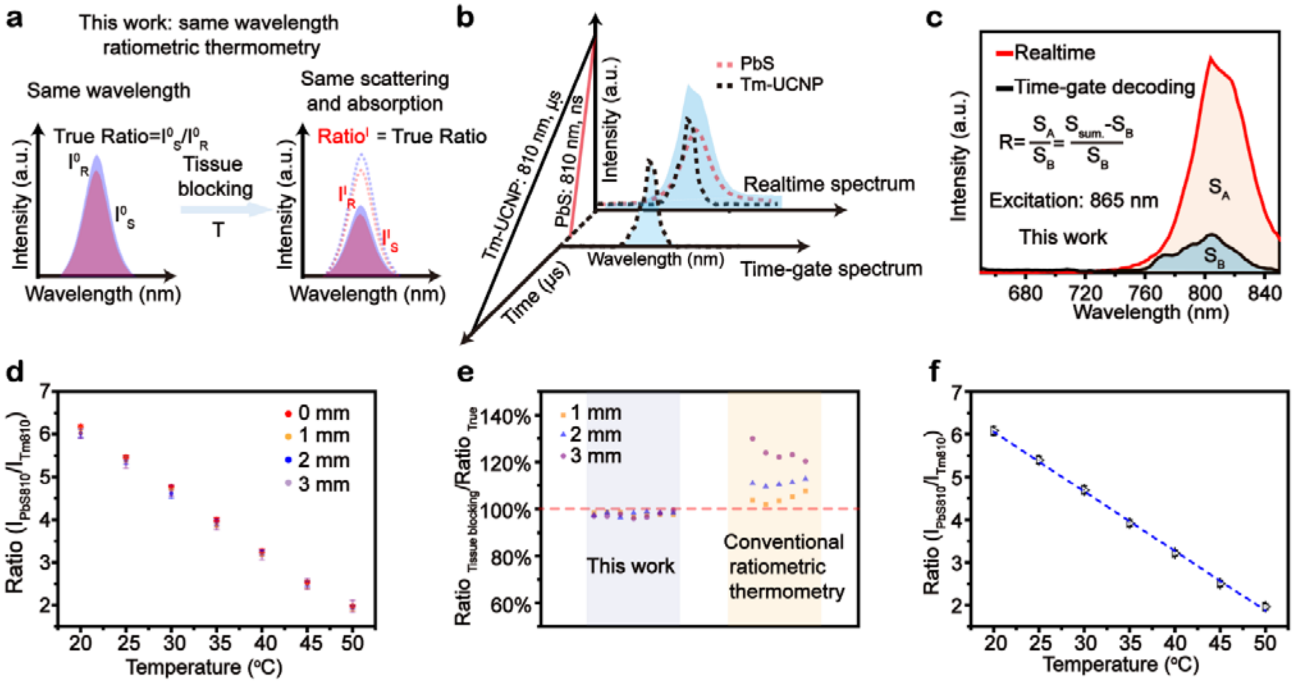

Fig. 4 Temperature monitoring ability evaluation of the same wavelength ratiometric thermometry based on the UCL emission of UCL-NCs. (a) Schematic illustration of conventional ratiometric thermometry with the same wavelength emission. (b) Schematic diagram of UCL signals separation via time-resolved decoding. (c) UCL emssion signals decoding of UCL-NCs using time-resolved spectrometer. (d) The same wavelength ratio of I_{PbS} to I_{Tm} obtained time-resolved spectrometer at different temperature with various thickness pork blocking. Each data and error bar represents mean ± s.d. based on three times

measurements with an excitation of 865 nm laser on spectrometer. (e) Ratio values deviation with different thickness pork blocking to ratio signals measured without pork blocking using the same wavelength or conventional ratiometric thermometry. (f) A plot of I_{PbS810}/I_{Tm810} versus T to calibrate the temperature. Average values of I_{PbS810}/I_{Tm810} under different temperature were given to fit the calibration curve based on the spectra with different thickness pork blocking. Error bars were defined as s.d.

Comment 2. *The brightness of the here used nanostructures have to be given. What is the minimum excitation density that can be used to obtaine subtissue thermal sensing. How is related the thermal accuracy with the illumination intensity?*

Response: We appreciate this valuable advice. It is necessary for a ratiometric luminescent thermometer to investigate power dependent spectra both of sensor and reference signals, owing to the reduced power density of excitation light after penetrating the tissue. In principle, for UCL emission, I_{em} is proportional to the n-th power of the NIR exciting power density. The data demonstrate that the relationship between UCL intensity and excitation power density of PbS QDs is linear (Figure S5a). For Tm-UCNPs, the result indicates that UCL intensity is quadratic depending on the excitation power (Figure S5b). However, under a low power excitation, a linear relationship can be fitted in mathematics (Figure S5b inset). The relationship between excitation power density and I_{Tm} was investigated in detail sub-1 W/cm^2 (100-900 mW/cm^2) at 25 ^{o}C (Figure S5c). The result reveals that I_{Tm} can also be fitted to show linear dependence on excitation power density. It should be noticed that the ratio of I_{PbS} to I_{Tm} is invariant under different excitation power density at 25 ^{o}C (Figure S6). These results show that the illumination intensity sub-1 W/cm^2 (100-900 mW/cm^2) will not affect the thermal accuracy. And the temperature calibration curve is measured under an illumination intensity of 100 mW/cm^2.

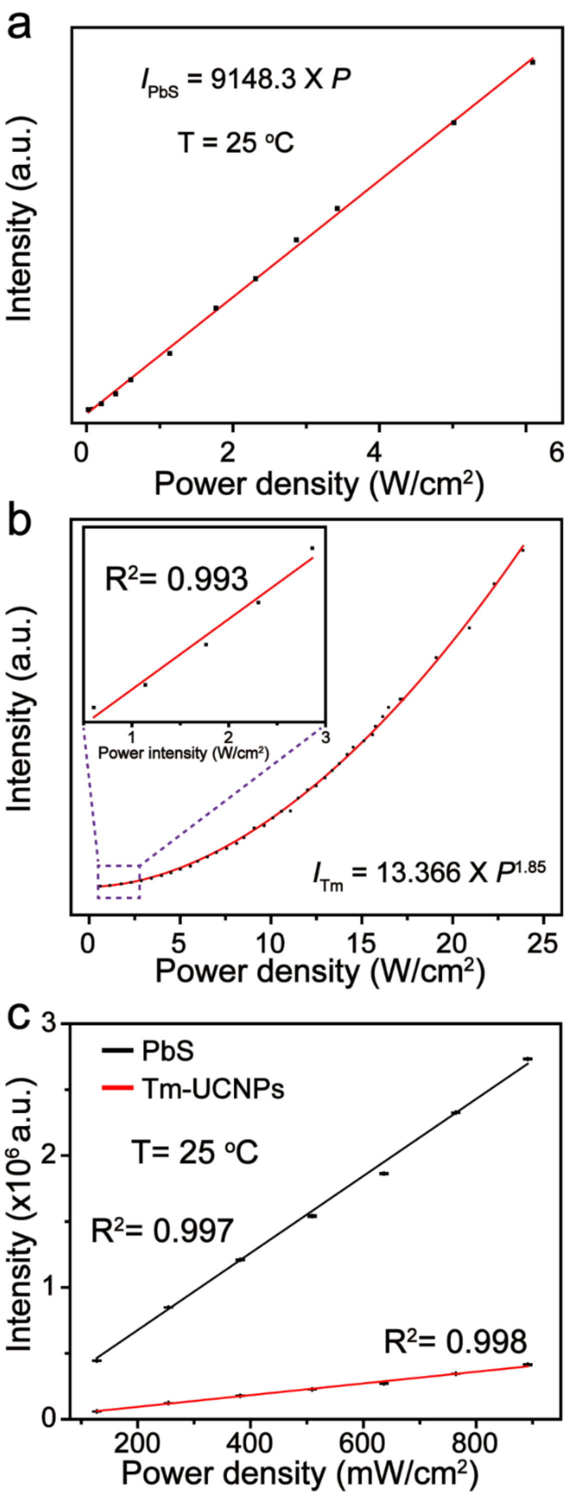

Figure S5. A plot of I_{UCL} versus P to investigate the effect of power density to UCL emission of (a) PbS and (b) Tm-UCNPs. The inset of (b) shows a linear dependent in mathematics under a low power density excitation. (c) Plots of UCL emission of PbS QDs and Tm-UCNPs as a function of excitation power density of the 865 nm laser.

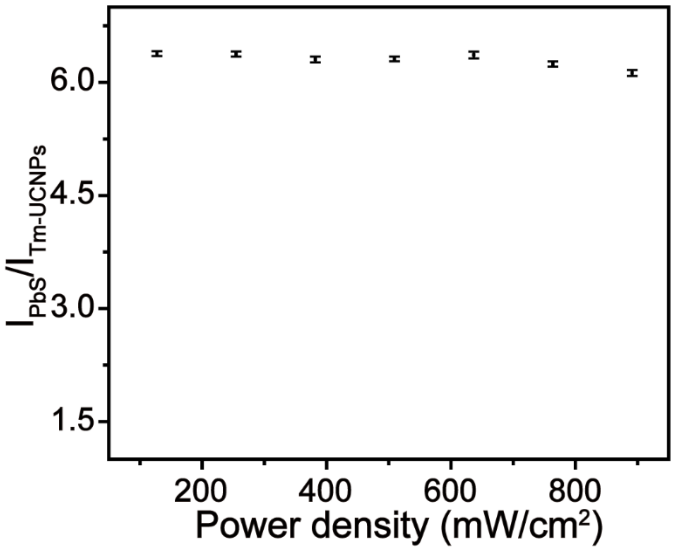

Figure S6. Ratios calculated from $I_{PbS}/I_{Tm\text{-}UCNPs}$ under different power density excitation at 25 °C.

Comment 3. *In the experimental data included in Figure 4e the time resolution seems to be of tens of seconds. Why is that? Is there any limitation not discussed that avoids real time thermal sensing based on this sensing system?*

Response: We appreciate this valuable advice. The time in Figure S13 is the irradiation time of 865 nm laser (0.5 W/cm^2) without modulation. The time-gate using in our manuscript is 20 μs. The limitation that avoids real time thermal sensing based on this sensing system is the penetration depth of tissue. When the thickness of tissue is greater than 3 mm, the errors increase owing to the sharply attenuating of the signals.

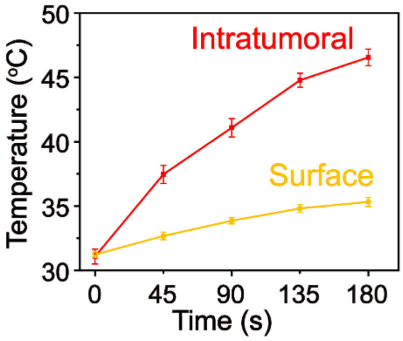

Figure S13. Intratumoral and skin surficial temperature measured during the PPT of the tumor area. The data were collected using the home built time-gate imaging system and a thermographic camera, respectively. Average values of intratumoral and skin surficial temperature at different time points were given based on three times measurement. Error bars were defined as s.d.

Comment 4. *What is exactly the heating source in their in vivo experiments? If it is associated to the heating efficiency of their nanostructures then this should be described and evaluated.*

Response: We appreciate this valuable advice. The exactly heating source in our in vivo experiments is an 865 nm laser (0.5 W/cm^2). The photothermal ability was just used to bring in a temperature change in vivo. As shown in Figure S10, the temperature increment is enough using an 865 nm laser (0.5 W/cm^2) as the heating source.

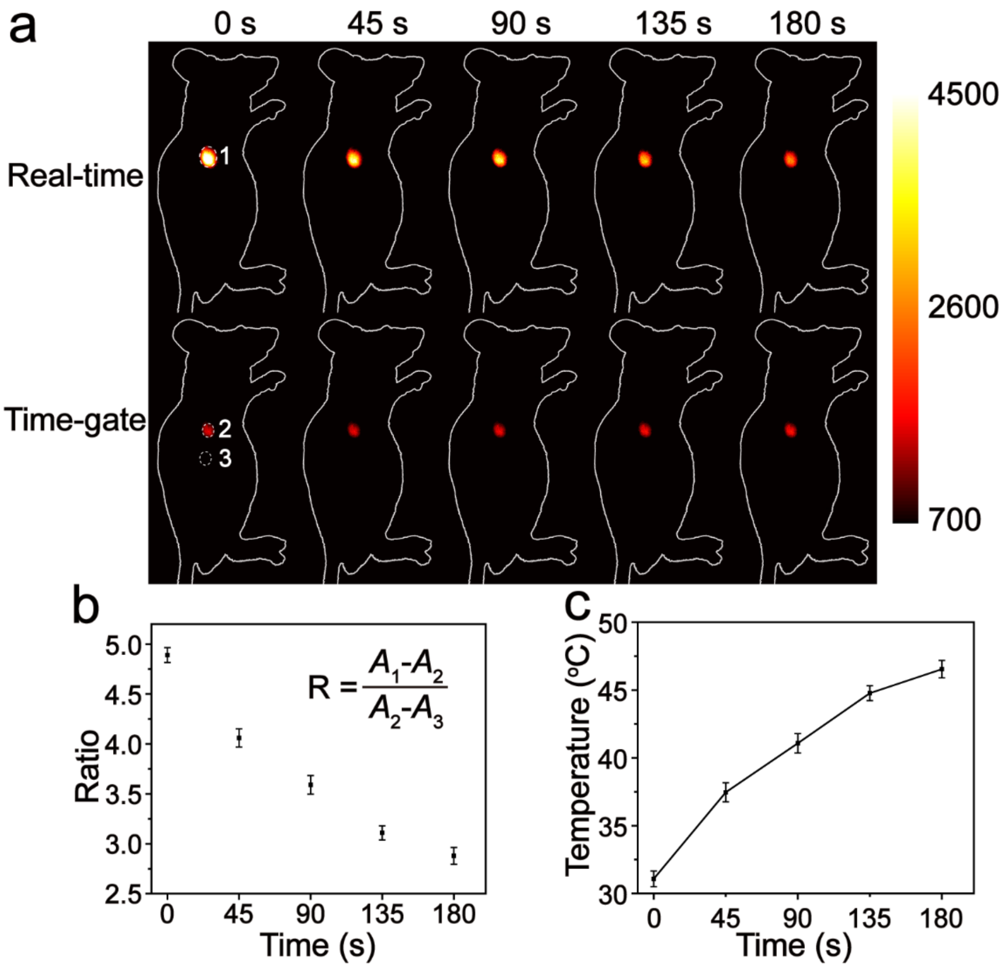

Figure S10. Intratumoral temperature monitoring *in vivo* during the PTT. (a) Real-time and time-gate UCL images of nude mice with the injection of UCL-NCs at different time under the irradiation of an 865 nm laser (0.5 W/cm^2). (b) Calculated ratio of I_{PbS} to $I_{Tm\text{-}UCNPs}$ obtained from figure (a). (c) The intratumoral temperature at different time during the PTT.

Comment 5. *What is the actual penetration depth of their structures?*

Response: We appreciate this valuable advice. The true depth of tissue is difficulty to determine owing to the wide distribution of the nanostructures *in vivo*. In order to eliminate the influence of light absorption and light scattering when penetrating the tissue, we developed this same wavelength

ratiometric thermometry. For subcutaneous tumor in nude mouse, the penetration depth is supposed to sub 3 mm.

Reviewers' comments:

Reviewer #1 (Remarks to the Author):

The revised version of the manuscript was substantially improved and the innovative degree of the paper is now more evident. The data reported demonstrate for a specific example some advantages of the same wavelength ratiometric nanothermometry approach for in vivo experiments, relatively to the conventional technique of different wavelength ratio.

The reported thermal relative sensitivity and temperature uncertainty values are better than those reported up to now using the conventional approach, which is probably related to the tissues heterogeneity at different wavelengths. The conclusions, and in general the tone of the paper's message, are, however, excessive. In the studied hybrid heater-thermometer platform with PbS QDs and NaYbF4:0.5%Tm@NaYF4:10%Yb@NaYF4:50%Nd upconversion NPs the figures of merits of the same same wavelength ratiometric nanothermometry approach are higher but the extrapolation of these results for other systems is highly speculative. I strong recommend extreme caution in this regard. With this modification, I support the publication of the paper.

Reviewer #2 (Remarks to the Author):

In the manuscript, a new strategy to fabricate a ratiometric upconversion luminescence nanothermometry is provided based on a hybrid structure composed of upconversion emissive PbS quantum dots and NaYbF4:0.5%Tm@NaYF4:10%Yb@NaYF4:50%Nd nanoparticles. The ratiometric signal composed of two upconversion emissions working at the same wavelength but different luminescent lifetimes are decoded via a time-resolved technique. In the responses, the main point shown in this manuscript is emphasized to be the accurate detection of temperature in vivo. However both the detection of temperature in vivo and ratiometric nanothermometry have been reported previously. The originality of this manuscript is still not enough, and this manuscript should be published elsewhere.

Some points about the response may be useful.

1. It is illustrated that the same wavelength NIR emission ratio upconversion luminescent nanothermometry in this work eliminate f(a) and f(s), which cannot be eliminated by the dual emission ratio luminescent nanothermometers. While, its influences on f(p) and f(c), which could be eliminated by the dual emission ratio luminescent nanothermometers, are not mentioned. The data in Table 1 also proved that the dual emission ratio luminescent nanothermometers could exhibit relatively high thermal sensitivity. So, the response to comment 1, “could its sensor accuracy be further improved with both the two strategies combined” is not clear enough.

2. The dynamic light scattering data in Figure R1 demonstrated the sizes of UCL-NCs and UCL-NCs@SiO2, confirming the size increment after SiO2-PEG modification. However, it is not reasonable to show a representative TEM images with nanoparticles of smaller sizes.

3. TEM images of the nanoparticles before and after warming-cooling cycles may be more visualized to confirm the stability.

4. More details about the experiments are needed.

Reviewer #3 (Remarks to the Author):

Authors have properly address my previous comments. In my opinion the paper is ready for publication.

Responses to the Reviewers' Reports **Manuscript** (NCOMMS-19-08975A-Z)

Reviewer #1:

The revised version of the manuscript was substantially improved and the innovative degree of the paper is now more evident. The data reported demonstrate for a specific example some advantages of the same wavelength ratiometric nanothermometry approach for in vivo experiments, relatively to the conventional technique of different wavelength ratio.

The reported thermal relative sensitivity and temperature uncertainty values are better than those reported up to now using the conventional approach, which is probably related to the tissues heterogeneity at different wavelengths. The conclusions, and in general the tone of the paper's message, are, however, excessive. In the studied hybrid heater-thermometer platform with PbS QDs and $NaYbF_4$:0.5%Tm@$NaYF_4$:10%Yb@$NaYF_4$:50%Nd upconversion NPs the figures of merits of the same wavelength ratiometric nanothermometry approach are higher but the extrapolation of these results for other systems is highly speculative. I strong recommend extreme caution in this regard. With this modification, I support the publication of the paper.

Response: We are very grateful to the reviewer for the positive evaluation and helpful suggestions. According to the reviewer's suggestion, we have modified some sentences that express excessive in our manuscript, especially in the discussion section. We have also paid attention to the wording of the article and modified the way of expression. Moreover, we deleted the extrapolation of our results for other systems in the discussion section. Details are highlight in our revised manuscript and some typical changes are shown as following.

In the manuscript:

"In order to minimize these errors and monitor temperature *in vivo*" (P.1, Line 10)

"This nanothermometer improves the temperature monitoring ability and a thermal resolution of ~0.3 K and a thermal sensitivity of ~5.6% K^{-1} is obtained *in vivo*" (P.1, Line 15)

"owing to reducing the impact of extrinsic factors like sensor concentration" (P.2, Line 22)

"Owing to minimizing the influence of *f*(*p*) and *f*(*c*)" (P.2, Line 24)

"However, f(a) and f(s) which depend on wavelength still cannot be avoided owing to the great difference in wavelength between the ratiometric emission" (P.2, Line 26)

"To minimize the errors originating from different wavelength in dual emission ratio nanothermometer" (P.3, Line 11)

"This dual emission ratiometric nanothermometry demonstrates a good temperature measurement ability in tissue and temperature mapping in tumor region is performed on mice *in vivo.*" (P.4, Line 4)

"a different attenuation is observed between 980 nm (Yb^{3+} emission) and 1280 nm" (PbS QD emission) (P.6, Line 10)

"these results suggest that it remains difficult for the different wavelength ratiometric thermometry to monitor the temperature accurately *in vivo*." (P.7, Line 22)

"the temperature calibration curve of UCL-NCs was investigated via performing several heating and cooling cycles on a time-resolved spectrometer." (P.9, Line 5)

"This result indicates that the same wavelength dual emissin ratiometric upconversion thermometry shows an excellent temperature monitoring ability and the errors origins from tissue blocking can be reduced." (P.11, Line 23)

"Therefore, the UCL-NC probe is capable of thermometry in vivo for monitoring intratumoral temperature." (P.12, Line 1)

Discussion

In summary, we demonstrated a same wavelength dual emission ratio upconversion luminescent nanothermometry system, which exhibits a more precise temperature detection *in vivo* and applied in the intratumoral temperature monitoring. Under the excitation of 865 nm laser, PbS QDs give a short lifetime temperature response UCL emission around 810 nm via single photon process and the Nd-sensitized Tm-UCNPs exhibit a long lifetime reference UCL signal around 810 nm. These two UCL emission at the same wavelength can be separated via time-resolved decoding owing to the different lifetime. The same wavelengths of excitation and emission ratiometric nanothermometer based on UCL-NCs shows an improved temperature measurement accuracy *in vivo*, and a temperature uncertainty *in vivo* of 0.3 K is obtained. It is worth noting that the thermal sensitivity of this hybrid system is up to 5.6 % K^{-1}. Furthermore, the NIR excitation and emission have advantages in biological applications. After being modifying with PEG, this hybrid nanostructure material was used in intratumoral temperature monitoring *in vivo* during the photothermal therapy. Thus, the present work reports a more accurate upconversion luminescent nanothermometry and provides a new way in the construction the dual emission luminescent nanothermometer.

Reviewer #2:

In the manuscript, a new strategy to fabricate a ratiometric upconversion luminescence nanothermometry is provided based on a hybrid structure composed of upconversion emissive PbS quantum dots and $NaYbF_4$:0.5%Tm@$NaYF_4$:10%Yb@$NaYF_4$:50%Nd nanoparticles. The ratiometric signal composed of two upconversion emissions working at the same wavelength but different luminescent lifetimes are decoded via a time-resolved technique. In the responses, the main point shown in this manuscript is emphasized to be the accurate detection of temperature in vivo. However both the detection of temperature in vivo and ratiometric nanothermometry have been reported previously. The originality of this manuscript is still not enough, and this manuscript should be published elsewhere.

Some points about the response may be useful.

Response: We appreciate the reviewer's helpful and valuable comments. In the revised manuscript, we have fully responded this comment as well as addressed the four comments by making supplementary experiments (*vide infra*). We are indebted to the Reviewer #2 for such remarkable improvement of our manuscript based on his/her comments. We regret however our revised manuscript could not convince Reviewer#2 of the originality in this work. Indeed, the accurate detection of temperature *in vivo* is based on the selection of ratiometric signal from two luminescent emission probes working at the same wavelength, which is not reported in any known literatures. Previous ratio temperature probes use filters to separate the temperature sensitive signal from the reference signal, which requires a large difference in wavelength between the two ratio signals. In these cases, it would cause errors due to different tissue absorption and scattering deriving from wavelength difference. In order to address this issue, our current work uses two same-wavelength signals with different lifetime to construct a dual-emission luminescence nanothermometers, which can furtherly improve the accuracy of temperature measurement. We believe this will certainly help the design of ratiometric luminescent probes working in a complex surroundings such as *in vivo* applications. In order to amphasize this point, we also made further revision below as the response to the reviewer's comments.

Comment 1: It is illustrated that the same wavelength NIR emission ratio upconversion luminescent nanothermometry in this work eliminate f(a) and f(s), which cannot be eliminated by the dual emission ratio luminescent nanothermometers. While, its influences on f(p) and f(c), which could be eliminated by the dual emission ratio luminescent nanothermometers, are not mentioned. The data in Table 1 also proved that the dual emission ratio luminescent nanothermometers could exhibit relatively high thermal sensitivity. So, the response to comment 1, "could its sensor accuracy be further improved with both the two strategies combined" is not clear enough.

Response: We are very sorry for our unclear expression on the same wavelength dual emission ratiometric nanothermometry. At first, our present work is based on the previous dual emission ratiometric nanothermometer, what's the improvement of our nanothermometer is that we use two same wavelength emission as the ratiometric signals. This means all the superiorities of the traditional dual emission ratio luminescent probes will maintained in our system, including the elimination of influences from f(p) and f(c).

Previous dual emission ratiometric nanothermometry use filters to separate the temperature sensitive signal from the reference signal, so it is unpractical to choose two emission at the same wavelength to fabricate a dual emission ratiometric nanothermometry. However, f(a) and f(s) which depend strongly on wavelength still cannot be eliminated owing to the difference in wavelength between the ratiometric emission. To minimize the influence of f(a) and f(s), in our present work, we chose two emission at the same wavelength with different lifetime to fabricate the dual emission ratiometric nanothermometry. The two signals were separated using time-resolved technique owing to their different lifetime. So our nanothermometer still has the merits of previous dual emission ratiometric nanothermometers and the influences of f(c) can be eliminated owing to the constant ratio (I_{PbS}/I_{Tm}) of different concentration. What's more, the influences of f(p) can also be minimized under a low excitation power density sub-1 W/cm^2 as shown in Figure S5 and S6. The key point of our present work is that the influences of f(a) and f(s) originating from wavelength difference of the ratiometric emission can be minimized.

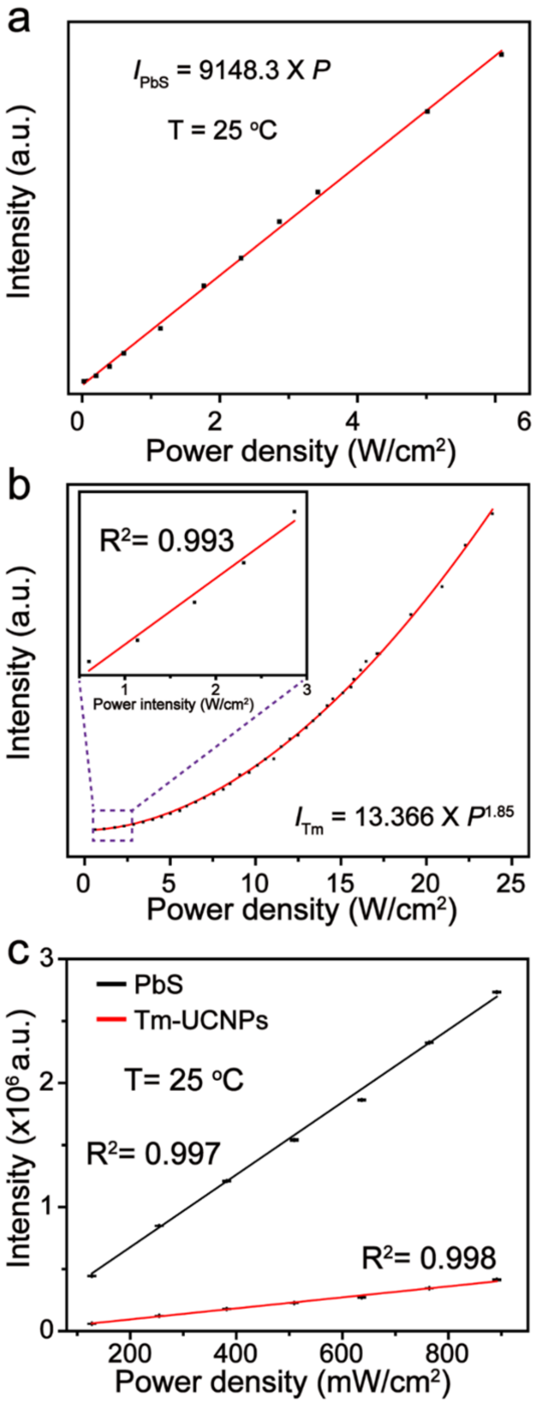

Figure S5. A plot of I_{UCL} versus P to investigate the effect of power density to UCL emission of (a) PbS and (b) Tm-UCNPs. The inset of (b) shows a linear dependent in mathematics under a low power density excitation. (c) Plots of UCL emission of PbS QDs and Tm-UCNPs as a function of excitation power density of the 865 nm laser.

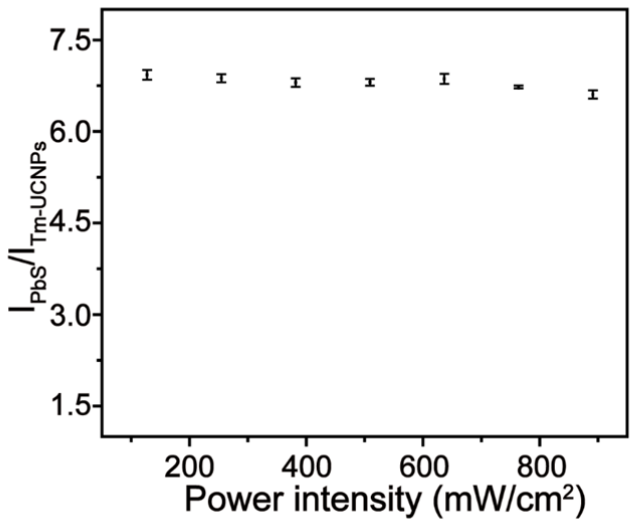

Figure S6. Ratios calculated from UCL emission of I_{PbS} to $I_{Tm\text{-}UCNPs}$ under different power density excitation at 25 °C.

It should also be mentioned that the signals of different-wavelength dual emission ratio nanothermometer also can be separated using time-resolved technique. As shown in Figure R1, I_{sum} composed of long-lifetime I_1^0 and short-lifetime I_2^0 can be obtained from a real-time spectrum or imaging. I_1' (I_1'=a × I_1^0 , a<1) can be separated using a time-resolved technique owing to the different lifetime. Then I_2^0 can be calculated: I_2^0 =I_{sum} - I_1'/a. At last, ratio of I_2^0 to I_1^0 can be calculate as following:

$$\mathrm{R}\left(\frac{I_2^0}{I_1^0}\right) = \frac{I_{sum} - \frac{I_1'}{a}}{\frac{I_1'}{a}} = a\frac{I_{sum}}{I_1'} - 1$$

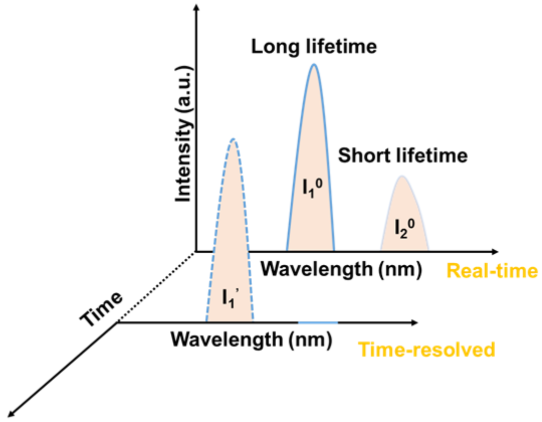

Figure R1. Schematic figure of separating signals of different-wavelength dual emission ratio nanothermometer using time-resolved technique.

The merit of time-resolved technique to separate two signals is that the ratiometric emission is no longer limited by wavelength, and using time-resolved technique to separate two signals with different wavelength shows no obvious merit comparing to using a filter. The advantages of time-resolved technique can be reflected in separating two signals with very close or same

wavelength, because the influences of f(a) and f(s) originating from wavelength difference of the ratiometric emission can be minimized.

So the answer to "could its sensor accuracy be further improved with both the two strategies combined" is that: **the sensor accuracy can be further improved with both the two strategies combined when the ratiometric signals have two emission with very close or same wavelength like our present work.** If using two different wavelength emission as ratiometric signals, time-resolved technique shows no obvious merits comparing to using filter.

In order to express the advantages of our same-wavelength dual emission ratio nanothermometer clearly, we have emphasized the advantages of dual emission ratio luminescent nanothermometers and made some changes in our revised manuscript as following:

"Dual emission ratio luminescent nanothermometers show resolvable emission from two different emitters, which can be used as a self-referencing ratio. The temperature sensitive signal change can be measured using ratio instead of absolute photoluminescence intensities, owing to reducing the impact of extrinsic factors like sensor concentration (*f*(*c*)), fluctuations of excitation power density (*f*(*p*)) or other local inhomogeneities that alter absolute intensities. Owing to minimizing the influence of *f*(*p*) and *f*(*c*), the dual emission ratio luminescent nanothermometers have been widely developed and attracted considerable interest." (P.2, Line 19)

"we provide a new strategy to fabricate the same-wavelength dual emission ratiometric upconversion" (P.1, Line 11)

"the same wavelength dual emission ratio upconversion luminescent nanothermometry" (P.3, Line 11)

"This dual emission ratiometric nanothermometry demonstrates a good temperature measurement ability in tissue and temperature mapping in tumor region is performed on mice *in vivo.*" (P.4, Line 4)

"Same wavelength dual emission ratiometric thermometry in vitro based on UCL-NCs. " (P.8, Line 13)

"the same wavelength dual emission ratiometric" (P.11, Line 1)

"This result indicates that the same wavelength dual emission ratiometric upconversion thermometry shows an excellent temperature monitoring ability and the errors origins from tissue blocking can be reduced." (P.11, Line 23)

"In summary, we demonstrated a same wavelength dual emission ratio upconversion luminescent nanothermometry system, which exhibits a more precise temperature detection *in vivo* and applied in the intratumoral temperature monitoring." (P.13, Line 4)

Comment 2: The dynamic light scattering data in Figure R1 demonstrated the sizes of UCL-NCs and UCL-NCs@SiO_2, confirming the size increment after SiO_2-PEG modification. However, it is not reasonable to show a representative TEM images with nanoparticles of smaller sizes.

Response: We appreciate the valuable and helpful comment. A new TEM image of UCL-NCs@SiO_2 was using in Figure 2. The average size of UCL-NCs is 94.3 ± 6.1 nm, and after coating a SiO_2 shell, the size increases to 114.1 ± 8.9 nm, which is agreement with the DLS result (Figure R2). For further confirming the successful coating of SiO_2, the EDS mapping of ULC-NCs@SiO_2 was performed. The result reveals a clear SiO_2 shell outside the UCL-NC.

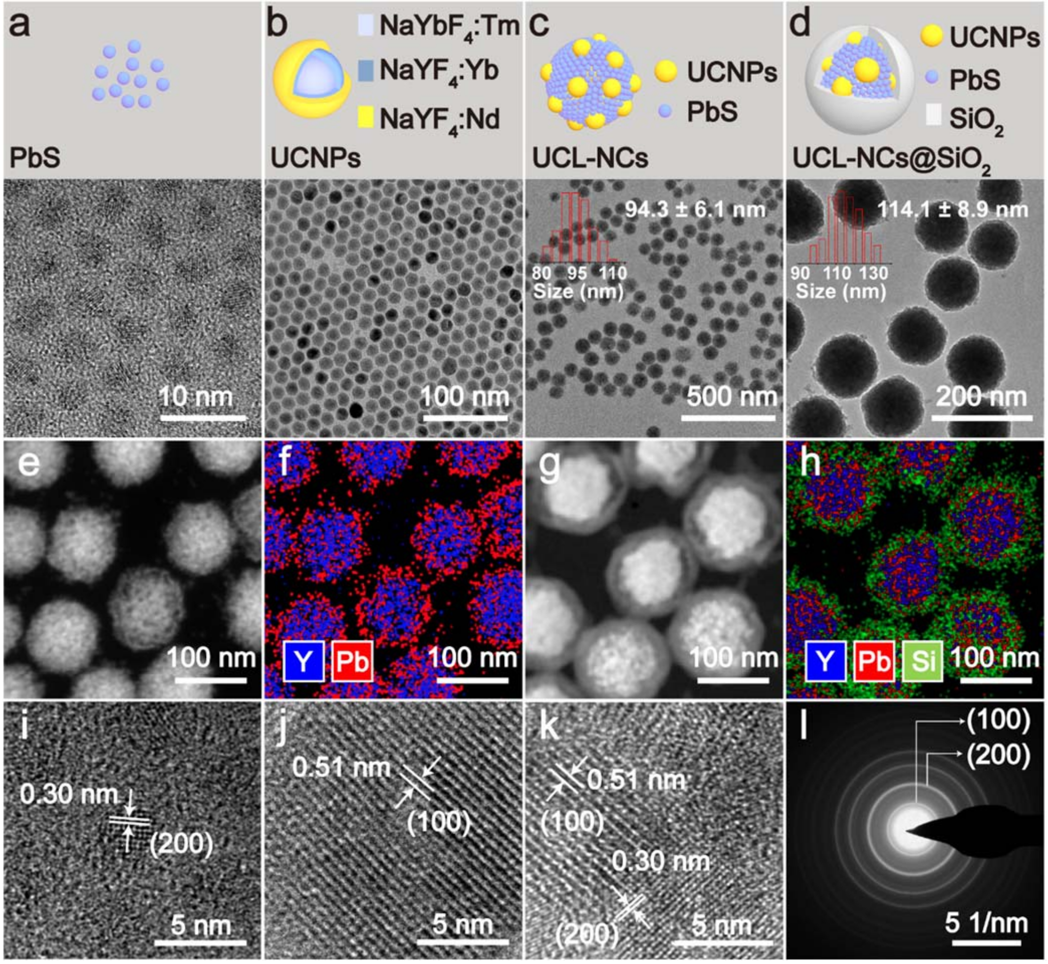

Figure 2. Characterizations of UCL-NCs. TEM images of (a) PbS QDs, (b) core-shell-shell Nd sensitized Tm-UCNPs, (c) UCL-NCs and (d) mPEG-silane functionalized silica-coated UCL-NCs. Images of dark-field scanning TEM and corresponding EDS elemental mapping of (e and f) UCL-NCs and (g and h) UCL-NCs@SiO_2. HR-TEM images of (i) PbS QDs, (j) core-shell-shell Nd sensitized Tm-UCNPs and (k) UCL-NCs. (l) Electron diffraction image of UCL-NCs.

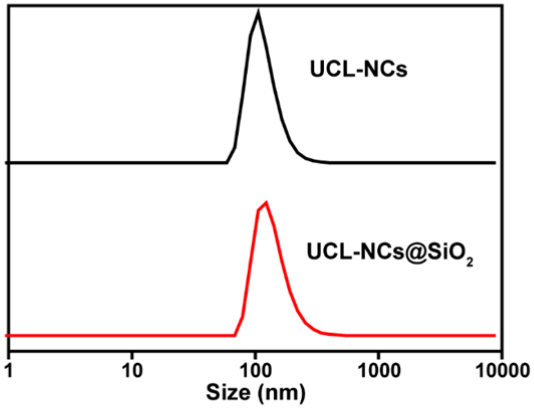

Figure R2. Dynamic light scattering data of UCL-NCs and UCL-NCs@SiO_2.

Comment 3: TEM images of the nanoparticles before and after warming-cooling cycles may be more visualized to confirm the stability.

Response: We appreciate this important and helpful comment. According to the reviewer's recommendation, TEM image (Figure S9a), dark-field scanning TEM image (Figure S9b) and corresponding EDS elemental mapping (Figure S9c-f) of UCL-NCs@SiO_2 after warming-cooling cycles were performed. These results exhibit no obvious difference with the nanoparticles before warming-cooling cycles (Figure 2d, 2g and 2h), which reveals the stability of this structure.

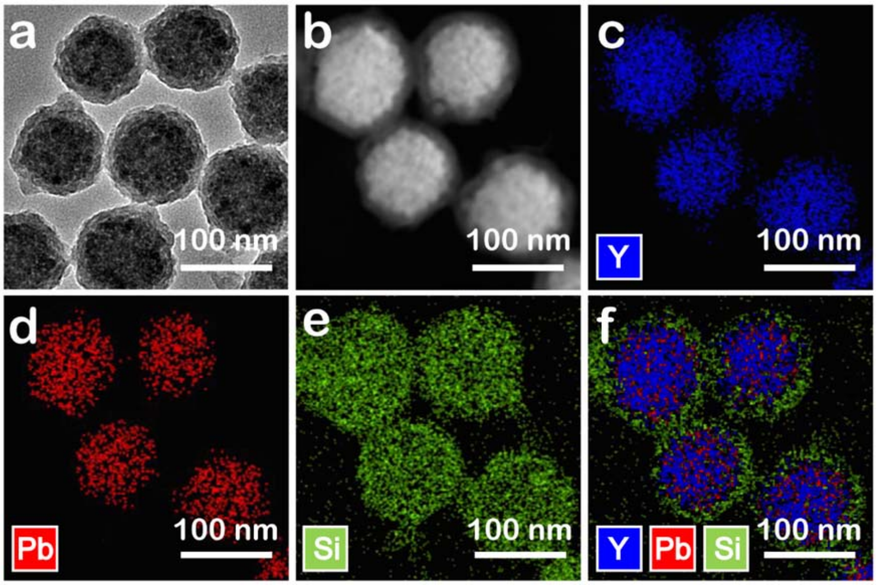

Figure S9. TEM image, dark-field scanning TEM image and corresponding EDS elemental mapping of (c, d, e and f) UCL-NCs@SiO_2 after warming-cooling cycles.

Comment 4: More details about the experiments are needed.

Response: We appreciate this important and helpful comment. According to the reviewer's suggestion, we have added further details about the experiments as below:

Temperature monitoring *in vitro*. To obtain the same wavelength ratiometric temperature calibration curve in aqueous solution, a quartz cuvette containing UCL-NCs@SiO_2 aqueous dispersion (2 ml, 0.5 mg/ml) was placed in home-built time-resolved spectrometer or home-built time-resolved bioimaging system (emICCD camera, PI-MAX4-1024B/EM, Princeton Instruments) with an external temperature controller. Aqueous solution was heated to different temperature from 20 to 50 ^{o}C and the corresponding real-time or time-resolved UCL round 810 nm was collect with excitation of a continuous wave (CW) 865 nm laser (100 mW/cm^2). The ratio of UCL emission peaks centred at 810 nm of PbS QDs and Tm-UCNPs as function of temperature are used as calibration curve for temperature monitoring. Different thickness of pork meat covering was used to simulate the tissue blocking *in vivo*. The conventional ratiometric temperature calibration curves were obtained on Edinburgh FLS-920 fluorescence spectrometer under the irradiation of an 865 nm laser (100 mW/cm^2).

Tumour xenografts. Animal procedures were in agreement with the guidelines of the Institutional Animal Care and Use Committee, School of Pharmacy, Fudan University. S180 cells were harvested by incubation with 0.05% trypsine-EDTA when they reached near confluence, and were collected by centrifugation and re-suspended in sterile PBS. Cells (~10^7 cells per site) in 50 μL in modified Eagle's medium were then subcutaneously implanted into 4-week-old male athymic nude mice. Photothermal therapy was performed when tumors reached 0.6 cm in average diameter.

Eigen temperature monitoring *in vivo*. Eigen temperature monitoring in vivo was conducted in UCL-NCs@SiO2 injected S180 tumour-bearing nude mice. 0–5W adjustable CW 865 nm lasers was chosen as the excitation source of UCL and photothermal excitation (0.5 W/cm^2). Real-time and time-gate UCL imaging were performed on a homebuilt time-resolved bioimaging system (emICCD camera, PI-MAX4-1024B/EM, Princeton Instruments) with an 810 ± 10 nm band-pass filter at different time point. The intratumoral temperature at different time point were calculated by the UCL images with the temperature calibration curve got in vitro simulated with pork meat covering.

Photothermal therapy *in vivo*. An optical fibre-coupled 865 nm diode-laser (Changchun New Industries Optoelectronics Technology Co., Ltd.) was used to irradiate tumours during the experiments. For treatment group, 50 μL of UCL-NCs@SiO_2 (2 mg/mL) aqueous solution was injected via intratumoral injection 2 hours before the irradiation. For photothermal treatment, the 865 nm laser beam with a diameter of ~10 mm was focused on the tumour area at the power density of 0.5 W/cm^2 for 3 min. FLIR E40 thermal imaging camera was used to collect Infrared thermal images. Tumour sizes of treatment group and reference groups (untreated mice, mice irradiated to the 865 nm laser only, and UCL-NCs@SiO_2 treated mice without 865 nm irradiation) were measured every day after treatment. Each group contained five mice for relatively rational evaluation. The tumour sizes were measured using a vernier caliper. Then the volume of tumor was calculated as following: volume = (tumour length) × (tumour width)^2/2. Relative tumour volumes were normalized and were calculated as V/V_0 (V_0 is the initiated tumour volume).

Characterization: TEM images, HRTEM images, HAADF-STEM images, and EDX mapping of the nanocrystals were characterized on a Tecnai G2 F20 S-Twin (FEI, America) operating at 200 kV. Fourier-transform infrared spectroscopy was performed using an IR Prestige-21 spectrometer (Shimadzu) from samples in KBr pellets. DLS was carried out on a Malvern Zetasizer Nano ZS

system. UCL spectra of UCL-NCs were measured with an Edinburgh LFS-920 fluorescence spectrometer. Infrared thermal images were collected FLIR E40 thermal imaging camera.

Reviewer #3 (Remarks to the Author):

Authors have properly address my previous comments. In my opinion the paper is ready for publication

Response: We are very grateful to the reviewer for his/her positive evaluation.

REVIEWERS' COMMENTS:

Reviewer #1 (Remarks to the Author):

Authors have properly address my previous comments. In my opinion the paper is ready for publication.

Reviewers' comments:
Reviewer #1 (Remarks to the Author):

The work of Xiaochen Qiu et al. describes the fabrication of a ratiometric upconversion luminescence nanothermometry based on a hybrid structure formed by spherical clusters of upconversion PbS QDs and NaYbF4:0.5%Tm@NaYF4:10%Yb@NaYF4:50%Nd NPs embedded in a polyethylene glycol modified silica shell and its use to measure the temperature in vivo (on xenograft S180 tumor mouse) during photothermal experiments.
Temperature in-situ measurements during photothermal processes in vivo were already reported (see, for instance, refs. 24-26 of the paper) with relative thermal sensitivity values analogous to that reported here (for instance, 10.1002/adfm.201702249). The pros and cons of hybrid nanothermometers (refs. 31 and 34 of the manuscript, for instance) and heater-thermometer nanoplatforms (e.g., 10.1021/acsnano.5b00059) were already discussed and in vivo photothermal heating, tumor localization through luminescence, intratumoral thermal sensing and photothermal therapy of tumors were also demonstrated (references mentioned above and 10.1002/adom.201500726, as examples). The degree of novelty of the work is, thus, low, being the findings reported essentially incremental (even accordingly to the work done before by the authors). Although not entirely clear the principal novelty seems to be the way as the ratiometric signal is measured (the two upconversion emissions working at the same wavelength are decoded via a time-resolved technique). With that said, I have serious doubts about the significance of this work for Nature Communications, but the judgement about the mismatches between the degree of novelty of a certain paper and the journal standards is essentially an editor's issue. In any case, I would like to encourage authors to consider the following points:

1. The advantages of the way of measuring the ratiometric signal relatively to what is reported before must be clearly stated.
2. The hybrid heater-thermometer platform is a complex structure. What is the ratio of PbS QDs and NaYbF4:0.5%Tm@NaYF4:10%Yb@NaYF4:50%Nd upconversion NPs (UCNPs) per cluster? Why the excitation is adjusted adding the Nd shell? Is that tuning dependent of the shell thickness? What is the role of the Yb-based buffer? What is the homogeneity of the distribution of QDs and UCNPs and how this determines the light-to-heat conversion efficiency?
3. The spectra presented in Figure 3 are measured in the isolated particles or in the clusters? Is there any difference between the thermometric response of the isolated particles relatively to what happens in the heater-thermometer clusters?
4. The calculus of IPbS is not explained (page 9).
5. How the temperature uncertainty is calculated (a reference is missing)? The temperature dependence of the relative sensitivity and temperature uncertainty should be represented.
6. The reported photothermal conversation efficiency values are common (much smaller than those reported for other lanthanide-based NPs, 10.1039/c7nr02630g and 10.1002/adom.201500726).

Reviewer #2 (Remarks to the Author):

In the manuscript, a new strategy to fabricate a ratiometric upconversion luminescence nanothermometry is provided based on a hybrid structure composed of upconversion emissive PbS quantum dots and NaYbF4:0.5%Tm@NaYF4:10%Yb@NaYF4:50%Nd nanoparticles. The ratiometric signal composed of two upconversion emissions working at the same wavelength but different luminescent lifetimes are decoded via a time-resolved technique. Although it is illustrated to avoid the absorption and scattering of complicated tissue limitations in vivo, there have been existed many different types and various delicate-designs about the upconversion luminescence nanothermometry until now. This work lacks of originality and details are insufficient, and this manuscript should be published elsewhere

1. The nanothermometry here demonstrates a more precise temperature measurement in tissue by working with a time-resolved technique instead of the difference in wavelength in previous nanothermometry, could its sensor accuracy be further improved with both the two strategies combined?
2. It is revealed that both IPbS and ITm have a strong dependence on temperature from temperature dependent UCL spectra of PbS QDs, core-shell-shell Nd sensitized Tm-UCNPs, and the plots of IUCL versus T of PbS QDs and Tm-UCNPs. While, the nanothermometry works via a time-resolved technique, the corresponding plots calculated from time-resolved spectrometer may be more important.
3. The experiments in vivo without UCL-NCs also showed a significantly surficial temperature-increment, which is not that different from the treatment group only estimating from the thermal images, but induced an entirely different result. Details are insufficient.

4. With an approximate 8 nm-thick polyethylene glycol (PEG) modified silica shell, it demonstrated that the final UCL-NCs@SiO2 showed almost invisible increase and even shrink in size in TEM images Fig.2C and D.
5. The data provided in Fig. S10 indicates the photothermal stability is not perfect, and much more information about the stability (photothermal stability and structural stability) is needed.

Reviewer #3 (Remarks to the Author):

In this paper authors have developed a novel nanostructure for high sensitivity ratiometric thermal sensing. It is based on the combination of upconverting rare earth nanoparticles ad infrared emitting PbS QDs. The different quenching mechanisms of these structures makes possible thermal reading from a ratiometric approach. Instead of acquiring the ratiometric parameter from a simple analysis of the emission spectrum of their composites (that is the result of the superposition of the emission of UCNPs and PbS QDs) authors use time decoding to elucidate the relative contributions of these two structures. The thermal sensitivity obtained is quite good (5% per degree) and authors used this structures to perform very preliminary in vivo experiments.
The structure here reported is interesting but I can not see the clear contribution of this work to the state of the field. I think authors should discuss with more detail the advantajes of their approach in respect to what is already published on the field. For instance:
1.- It is not clear why time-decoding is beneficial in respect to a simple spectroscopic analysis. The thermal sensitivity curves should be given as obtained by applying time decoding and simple spectral anlysis if CW emission spectra.
2. The brightness of the here used nanostructures have to be given. What is the minimum excitation density that can be used to obtaine subtissue thermal sensing. How is related the thermal accuracy with the illumination intensity?
3. In the experimental data included in Figure 4e the time resolution seems to be of tens of seconds. Why is that? Is there any limitation not discussed that avoids real time thermal sensing based on this sensing system?
4. What is exactly the heating source in their in vivo experiments? If it is associated to the heating efficiency of their nanostructures then this should be described and evaluated.
5. What is the actual penetration depth of their structures?

Responses to the Reviewers' Reports
Reviewer #1:
Comments:
The work of Xiaochen Qiu et al. describes the fabrication of a ratiometric upconversion luminescence nanothermometry based on a hybrid structure formed by spherical clusters of upconversion PbS QDs and NaYbF4:0.5%Tm@NaYF4:10%Yb@NaYF4:50%Nd NPs embedded in a polyethylene glycol modified silica shell and its use to measure the temperature in vivo (on xenograft S180 tumor mouse) during photothermal experiments.
Temperature in-situ measurements during photothermal processes in vivo were already reported (see, for instance, refs. 24-26 of the paper) with relative thermal sensitivity values analogous to that reported here (for instance, 10.1002/adfm.201702249). The pros and cons of hybrid nanothermometers (refs. 31 and 34 of the manuscript, for instance) and heater-thermometer nanoplatforms (e.g., 10.1021/acsnano.5b00059) were already discussed and in vivo photothermal heating, tumor localization through luminescence, intratumoral thermal sensing and photothermal therapy of tumors were also demonstrated (references mentioned above and 10.1002/adom.201500726, as examples). The degree of novelty of the work is, thus, low, being the findings reported essentially incremental (even accordingly to the work done before by the authors). Although not entirely clear the principal novelty seems to be the way as the ratiometric signal is measured (the two upconversion emissions working at the same wavelength are decoded via a time-resolved technique). With that said, I have serious doubts about the significance of this work for Nature Communications, but the judgement about the mismatches between the degree of novelty of a certain paper and the journal standards is essentially an editor's issue. In any case, I would like to encourage authors to consider the following points:
Comment 1. *The advantages of the way of measuring the ratiometric signal relatively to what is reported before must be clearly stated.*
Response: We appreciate this valuable advice. In order to clearly stated the advantages of the way of measuring the ratiometric signal relatively to what is reported before, in our revised manuscript, control temperature monitoring experiments of conventional and this same wavelength ratiometric nanothermometry are performed in details using pork covering to simulate the situation *in vivo*. (Fig. 3 and 4). As shown in those results, high accuracy of this same wavelength ratiometric nanothermometry *in vivo* are marked exhibited, owing to eliminating errors caused from different wavelength ratio. In detail, for conventional ratiometric nanothermometry, the temperature uncertainty is 5.6 K which makes it unpractical to monitor the precise temperature in vivo. In contrast to this, for the same wavelength ratiometric nanothermometry, a temperature uncertainty of 0.3 K is obtained owing to eliminating the errors from the effect of heterogeneity on different wavelengths, which exhibits an overwhelming advantage in temperature monitoring in vivo. Moreover, thermal sensitivity of this unique ratiometric nanothermometer is up to 5.6% K^{-1}, which is much higher than other conventional upconversion luminescence nanothermometers whose thermal sensitivity is about 1%. Finally, our manuscript exhibits the first example to fabricate an NIR upconversion luminescence ratiometric nanothermometer (See in Table 1) for monitoring temperature in biological system.

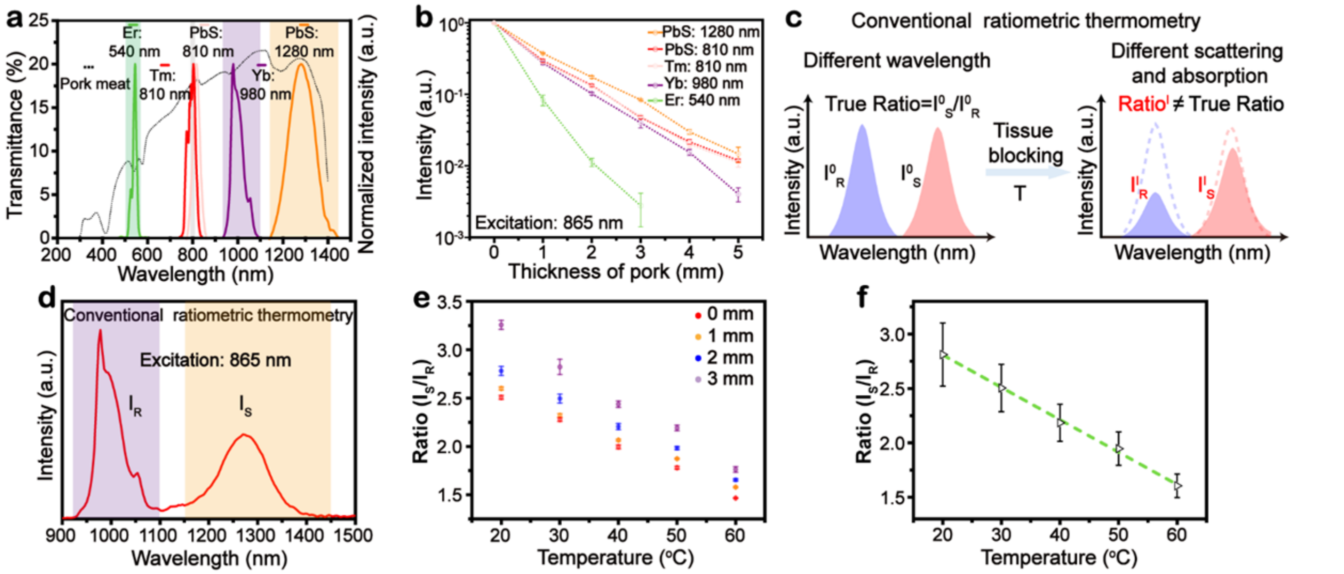

Fig. 3 Errors derived from the tissue blocking in conventional luminescent ratiometric thermometry. (a) Transmittance spectrum (left axis) of 1 mm pork and emission spectra (right axis) of Er-UCNPs, Tm-nanoparticles and PbS QDs. Colour shades correspond to the spectral ranges for collecting respective emissions. (b) Normalized intensities obtained from Er-UCNPs, Tm-nanoparticles and PbS QDs with respect to thickness of pork under the excitation of 865 nm laser. Each data and error bar represents mean ± s.d. based on three times measurements on spectrometer. (c)

Schematic illustration of conventional luminescent ratiometric thermometry with different wavelength emission. (d) Stokes emission spectrum of UCL-NCs under the excitation of 865 nm laser. I_R and I_S are the reference emission (Yb^{3+} emission, 980 nm) and temperature sensitive emission (PbS emission, 1280 nm), respectively. (e) Ratios of I_S to I_R at different temperature with various thickness pork blocking. Each data and error bar represents mean ± s.d. based on three times measurements on spectrometer. (f) A plot of I_S/I_R versus T to calibrate the temperature. Average values of I_S/I_R under different temperature were obtained from the ratios with different thickness (0, 1, 2 and 3 mm) pork blocking. Error bars were defined as s.d.

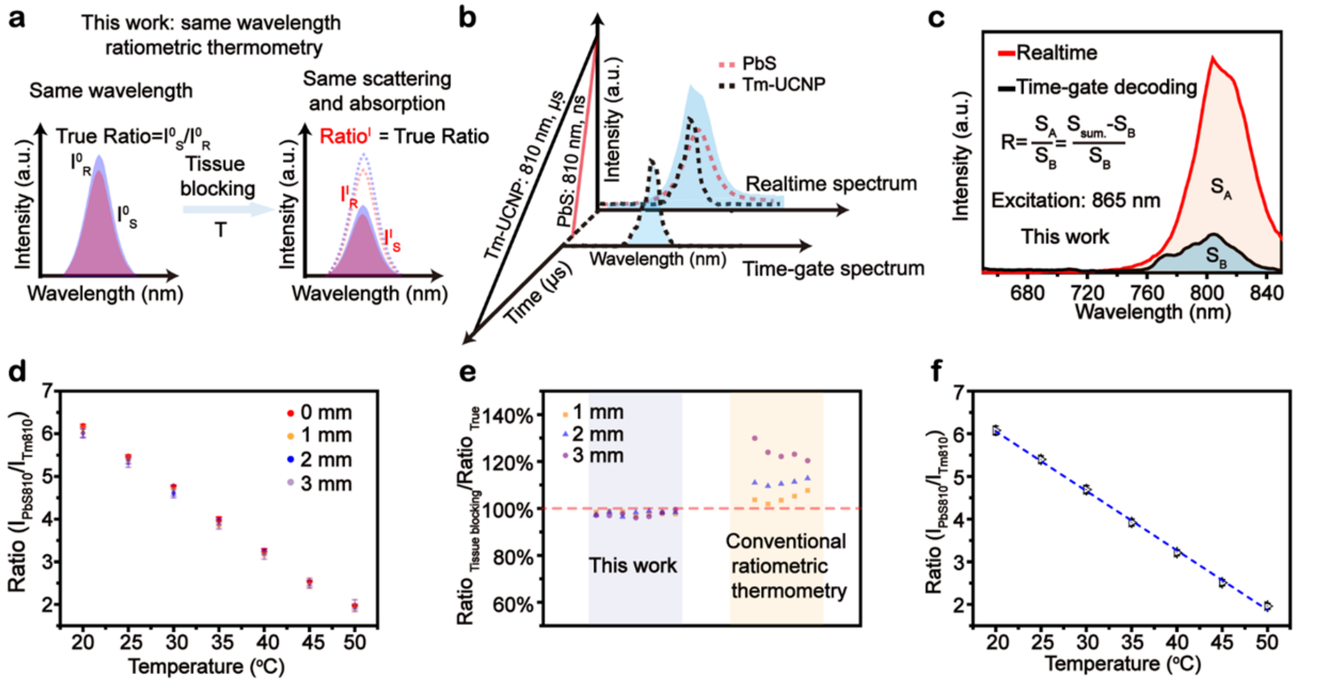

Fig. 4 Temperature monitoring ability evaluation of the same wavelength ratiometric thermometry based on the UCL emission of UCL-NCs. (a) Schematic illustration of conventional ratiometric thermometry with the same wavelength emission. (b) Schematic diagram of UCL signals separation via time-resolved decoding. (c) UCL emssion signals decoding of UCL-NCs using time-resolved spectrometer. (d) The same wavelength ratio of I_{PbS} to I_{Tm} obtained time-resolved spectrometer at different temperature with various thickness pork blocking. Each data and error bar represents mean ± s.d. based on three times measurements with an excitation of 865 nm laser on spectrometer. (e) Ratio values deviation with different thickness pork blocking to ratio signals measured without pork blocking using the same wavelength or conventional ratiometric thermometry. (f) A plot of I_{PbS810}/I_{Tm810} versus T to calibrate the temperature. Average values of I_{PbS810}/I_{Tm810} under different temperature were given to fit the calibration curve based on the spectra with different thickness pork blocking. Error bars were defined as s.d.

Table 1. Experimental parameters of several typical in vivo nanothermometers. $\lambda_{Ex.}$, $\lambda_{Em.}$, √, × and S_R correspond to excitation wavelength, emission wavelength, yes, no and relative thermal sensitivity, respectively.

Material	$\lambda_{Ex.}$ (nm)	$\lambda_{Em.}$ (nm)	NIR Ex. and Em.	Ratio	Same Em.	S_R (10^{-2} K^{-1})
PbS/CdS/ZnS QDs[26]	808	1270	√	×	-	1.0
PbS/CdS/ZnS-Nd NPs[34]	808	1060 and 1250	√	√	×	2.5
TTA-Nd-NPs[31]	635 and 808	540 and 1060	×	√	×	7.1
CsUCNP @C[24]	980	525 and 545	×	√	×	1.1
Er-Yb@Yb-Tm LaF_3[37]	690	1000, 1230 and 1550	×	√	×	5.0
UCL-NCs[This work]	865	810 and 810	√	√	√	5.6

Comment 2. *The hybrid heater-thermometer platform is a complex structure. (a) What is the ratio of PbS QDs and NaYbF4:0.5%Tm@NaYF4:10%Yb@NaYF4:50%Nd upconversion NPs (UCNPs) per cluster? (b) Why the excitation is adjusted adding the Nd shell? (c) Is that tuning dependent of the shell thickness? (d) What is the role of the Yb-based buffer? (e) What is the homogeneity of the distribution of QDs and UCNPs and how this determines the light-to-heat conversion efficiency?*

We appreciate this valuable advice. (a) The mass ratio of PbS QDs to UCNPs is 4:1 according to the materials adding in our synthesis procedure. (b) Typically, UCNPs are simultaneously doped with sensitizers (usually Yb^{3+} or Nd^{3+}) and activators (Er^{3+}, Tm^{3+}, or Ho^{3+} in most cases). The sensitizers absorb near-infrared (NIR) photons and then transfer the energy to the activators. Yb^{3+} has only one excited state ($^2F_{5/2}$), which results in the absorption band located at *ca.* 980 nm (from $^2F_{7/2}$ to $^2F_{5/2}$). However, for upconversion emissive PbS QDs, the thermal sensitivity is poor under the excitation with a 980 nm laser. In contrast to this, when exciting with an 865 nm laser, a high thermal sensitivity and good linearity are obtained between UCL intensity and temperature. So, Nd shell was added to absorb 865 nm photons owing to the matching energy level difference between $^4F_{3/2}$ and $^4I_{9/2}$. (c) The tuning is not dependent on the shell thickness. The excitation wavelength is determined by the intrinsic energy level of Nd^{3+}, which will not change with the thickness of Nd shell. (d) The Yb-based buffer between core and outer shell serves as a shield to prevent quenching interactions between the activator (Tm^{3+}) and sensitizer (Nd^{3+}). (e) QDs and UCNPs are distributed in cluster randomly and the light-to-heat conversion efficiency was calculated in Supporting Information Section 5 as following:

SI-5 Calculation of Photothermal Conversion Efficiency.

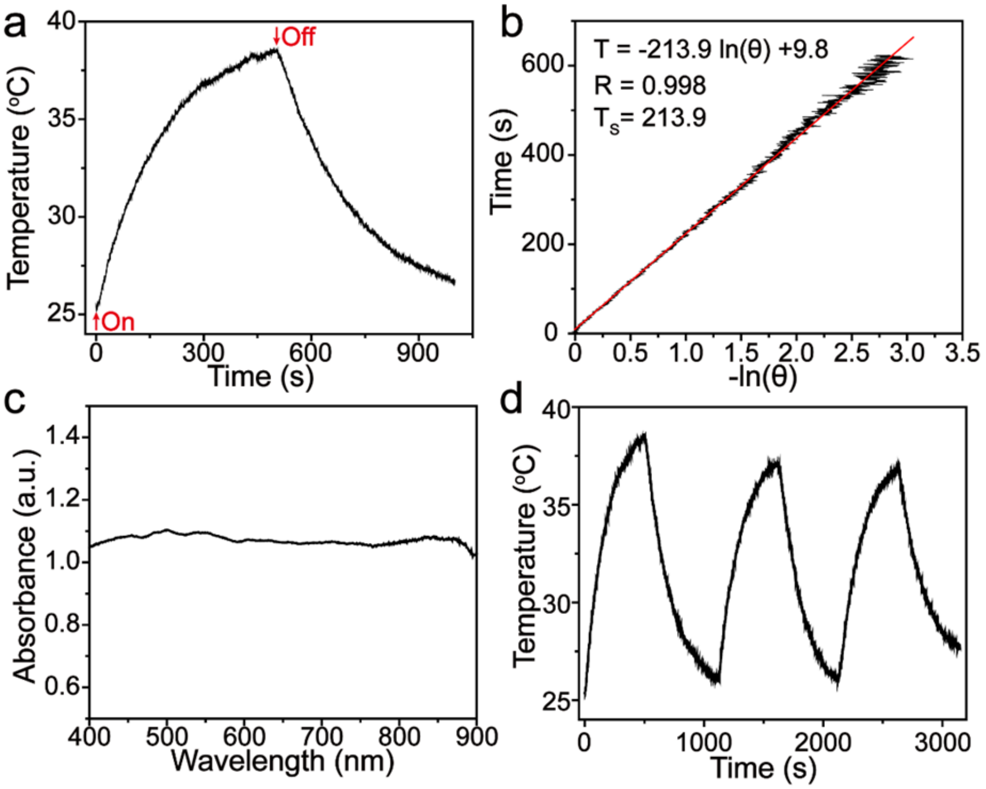

Figure S9. Characterization of photo thermal properties of UCL-NCs. (a) Temperature rising and cooling curve of UCL-NCs aqueous dispersion under irradiation of an 865 nm laser (0.8 W/cm^2). During the process, the irradiation lasted for 500 s, and then the laser was shut down. (b) Determination of the time constant for heat transfer from the cooling profile in Figure (a). (c) UV-NIR absorption spectrum of UCL-NCs aqueous dispersion. (d) Heat effect of UCL-NCs aqueous solution over 3 cycles of continuous re-irradiation (0.8 W/cm^2).

Disscussion. According to the method described in the literature, the total energy conservation for the system can be expressed by Supplementary Equation 1.

$$\sum_i m_i C_{p,i} \frac{dT}{dt} = Q_{NC} + Q_B - Qsur \quad (1)$$

where m and C_p are the mass and heat capacity of water respectively, T is the solution temperature, Q_{NC} is the energy induced by UCL-NCs, Q_B is the baseline energy induced by the sample cell, and $Qsur$ is heat conduction away from the surface by air. Q_{NC} is caused by non-radiative transition of PbS QDs under irradiation of 865 nm laser:

$$Q_{NC} = I(1 - 10^{-A_{865}})\eta \quad (2)$$

where I is the laser power, η is the conversion efficiency from incident laser energy to thermal energy, and A_{865} is the absorbance of UCL-NCs at wavelength of 865 nm. On the other hand, Q_B, expressing heat dissipated from light absorbed by the sample cell, was measured independently to be 8.5 mW using a quartz cuvette containing pure water

without UCL-NCs. Moreover, Q_{sur} is in proportion to temperature for the outgoing thermal energy, as given by Supplementary Equation 3.

$$Q_{sur} = hS(T - T_{\mathrm{amb}}) \qquad (3)$$

where h is heat transfer coefficient, S is the surface area of the container, and T_{amb} is ambient temperature of the surroundings.

According to Supplementary Equation 3, when the system temperature will reach a maximum, the heat input is equal to heat output:

$$Q_{NC} + Q_B = hS(T_{max} - T_{amb}) \qquad (4)$$

where T_{max} is the equilibrium temperature. The 865 nm laser heat conversion efficiency (η) can be determined by substituting Supplementary Equation 2 for Q_{NC} into Supplementary Equation 4 and rearranging to get

$$\eta = \frac{hS(T_{max} - T_{amb}) - Q_B}{I(1 - 10^{-A_{865}})} \qquad (5)$$

where Q_B was measured independently to be 8.5 mW, the (T_{max}-T_{amb}) was 13.2 °C according to Figure S9a, I is 800 mW cm^{-2}, A_{865} is the absorbance (1.074) of UCL-NCs at 865 nm (Figure S9c). Here, hS is calculated by introducing θ, is defined as the expression below:

$$\theta = \frac{T - T_{amb}}{T_{max} - T_{amb}} \qquad (6)$$

and a sample system time constant τ_s

$$\tau_s = \frac{\sum_i m_i C_{p,i}}{hs} \qquad (7)$$

which is substituted into Supplementary Equation 4 and rearranged to yield

$$\frac{d\theta}{dt} = \frac{1}{\tau_s}\left[\frac{Q_{cs} + Q_B}{hS(T_{max} - T_{amb})} - \theta\right] \qquad (8)$$

At the cooling stage of the aqueous dispersion of the UCL-NCs, the light source was shut off, the Q_{NC} +Q_B = 0, reducing the Supplementary Equation 9

$$dt = -\tau_s \frac{d\theta}{\theta} \qquad (9)$$

and integrating, giving the expression

$$t = -\tau_s \ln\theta \qquad (10)$$

Therefore, time constant for heat transfer from the system is determined to be τs = 213.9 s by applying the linear time data from the cooling period vs negative natural logarithm of θ (Figure S9b). In addition, the m is 1 g and the C is 4.2 J g^{-1}. Thus, according to Supplementary Equation 7, the hS is deduced to be 19.7 mW $°C^{-1}$. Substituting 19.7 mW $°C^{-1}$ into the hS into Supplementary Equation 5, the 865 nm laser heat conversion efficiency (η) of UCL-NCs can be calculated to be 34.4 %.

Comment 3. *The spectra presented in Figure 3 are measured in the isolated particles or in the clusters? Is there any difference between the thermometric response of the isolated particles relatively to what happens in the heater-thermometer clusters?*

We appreciate this valuable advice. Both in our previous and revised manuscript, temperature calibration curves are measured using the clusters using time-gate technique. From our results, both isolated PbS QDs and UCL-NCs have a good thermal sensitivity.

Comment 4. *The calculus of IPbS is not explained (page 9).*

We appreciate this valuable advice. $I_{\text{real-time}}$ of UCL-NCs contains both UCL emissions of UCNPs (long lifetime) and PbS QDs (short lifetime). Then I_{UCNPs} was obtained using a 20 µs time-gate to eliminate the short lifetime signal of PbS QDs. So I_{PbS} was calculated from $I_{\text{real-time}}$ minus $I_{\text{time-resolved}}$.

Comment 5. *How the temperature uncertainty is calculated (a reference is missing)? The temperature dependence of the relative sensitivity and temperature uncertainty should be represented.*

We appreciate this valuable advice. Temperature uncertainty is calculated as following:

$$\sigma T = \frac{\sigma R}{S_R}$$

$$S_R = \frac{dRatio}{dT}\frac{1}{Ratio}$$

where $dRatio/dT$ can get from the slope of temperature calibration curves. σR is the standard deviation of Ratio obatined from three times measurements. The thermal sensitivity and temperature uncertainty of this thermometer at 45 °C is about 5.6 % K^{-1} and 0.3 K.

Reference: C.D.S. Brites, A. Millan, L.D. Carlos, J.C.G. Bünzli, V.K. Pecharsky (Eds.), Handbook of the Physics and Chemistry of Rare Earths, Lanthanides in Luminescent Thermometry, vol. 49, Elsevier B.V., The Netherlands, 2016, pp. 339–427 (Chapter281).

Comment 6. *The reported photothermal conversation efficiency values are common (much smaller than those reported for other lanthanide-based NPs, 10.1039/c7nr02630g and 10.1002/adom.201500726).*

We appreciate this valuable advice. The photothermal conversation efficiency values reported in our manuscript are common. However, the main bright spot of our manuscript is the high accuracy of this same wavelength ratiometric nanothermometry in vivo. The photothermal ability was just used to bring in a temperature change *in vivo*.

Reviewer #2:

Comments:

In the manuscript, a new strategy to fabricate a ratiometric upconversion luminescence nanothermometry is provided based on a hybrid structure composed of upconversion emissive PbS quantum dots and NaYbF4:0.5%Tm@NaYF4:10%Yb@NaYF4:50%Nd nanoparticles. The ratiometric signal composed of two upconversion emissions working at the same wavelength but different luminescent lifetimes are decoded via a time-resolved technique. Although it is illustrated to avoid the absorption and scattering of complicated tissue limitations in vivo, there have been existed many different types and various delicate-designs about the upconversion luminescence nanothermometry until now. This work lacks of originality and details are insufficient, and this manuscript should be published elsewhere

Comment 1. *The nanothermometry here demonstrates a more precise temperature measurement in tissue by working with a time-resolved technique instead of the difference in wavelength in previous nanothermometry, could its sensor accuracy be further improved with both the two strategies combined?*

Response: Thank you for your careful work. The basic effects of accurate microenvironment temperature monitor *in vivo* are shown in the formula as following:

$$I_{in\ vivo} = \mathrm{f}(p)\cdot\mathrm{f}(c)\cdot\mathrm{f}(a)\cdot\mathrm{f}(s)\cdot\mathrm{f}(x)$$

In the formula, f(p), f(c), f(a), f(s) and f(x) correspond to excitation power density, material concentration *in vivo*, light absorption, light scattering and other influence factors. The dual emission ratio luminescent nanothermometers can eliminate the influence of f(p) and f(c), owing to its self-referencing capability. However, f(a) and f(s) which depend strongly on wavelength still cannot be eliminated owing to the great difference in wavelength between the ratiometric emissions. In order to eliminate f(a) and f(s), in our manuscript, the same wavelength NIR emission ratio upconversion luminescent nanothermometry is fabricated and explored *in vivo* via time-resolved decoding

Comment 2. *It is revealed that both IPbS and ITm have a strong dependence on temperature from temperature dependent UCL spectra of PbS QDs, core-shell-shell Nd sensitized Tm-UCNPs, and the plots of IUCL versus T of PbS QDs and Tm-UCNPs. While, the nanothermometry works via a time-resolved technique, the corresponding plots calculated from time-resolved spectrometer may be more important.*

Response: We appreciate this valuable advice. As shown in Fig. 4b and Fig. 4c, $I_{\text{real-time}}$ of UCL-NCs contains both UCL emissions of UCNPs (long lifetime) and PbS QDs (short lifetime). Then I_{UCNPs} was obtained using a 20 μs time-gate to eliminate the short lifetime signal of PbS QDs. So I_{PbS} was calculated from $I_{\text{real-time}}$ minus $I_{\text{time-resolved}}$. And the corresponding plots calculated from time-resolved spectrometer are shown in Figure 4d.

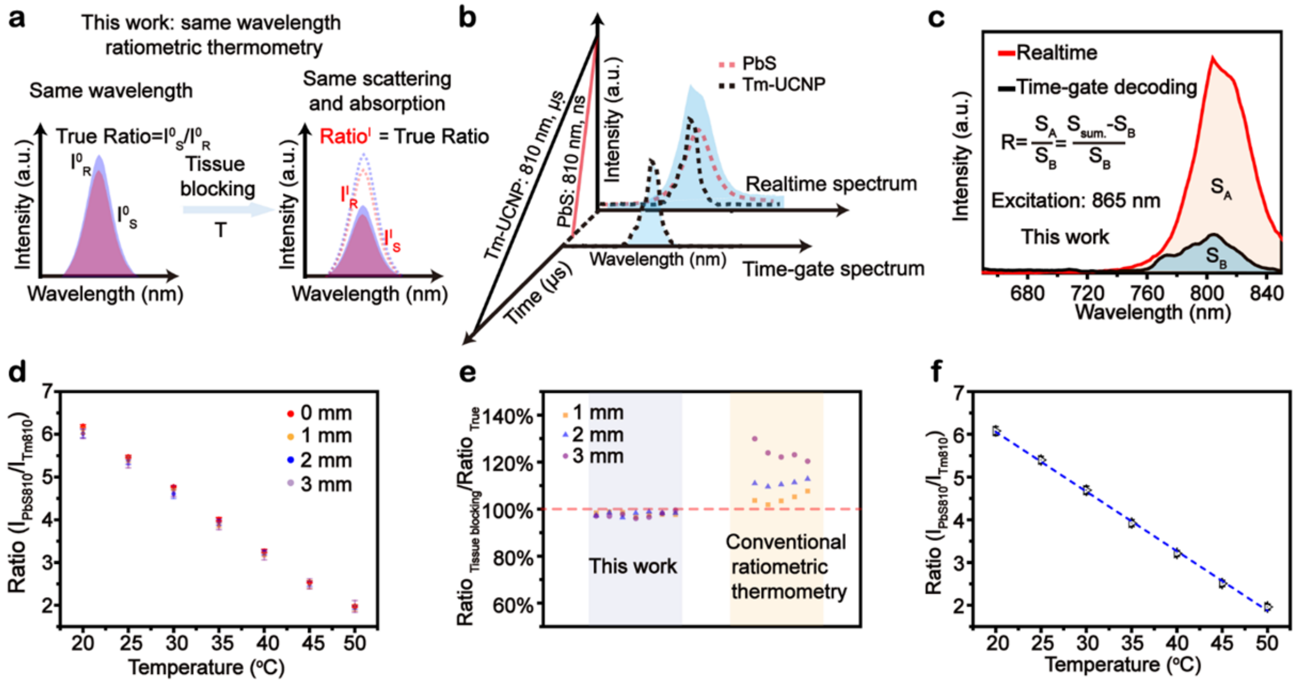

Fig. 4 Temperature monitoring ability evaluation of the same wavelength ratiometric thermometry based on the UCL emission of UCL-NCs. (a) Schematic illustration of conventional ratiometric thermometry with the same wavelength emission. (b) Schematic diagram of UCL signals separation via time-resolved decoding. (c) UCL emssion signals

decoding of UCL-NCs using time-resolved spectrometer. (d) The same wavelength ratio of I_{PbS} to I_{Tm} obtained time-resolved spectrometer at different temperature with various thickness pork blocking. Each data and error bar represents mean ± s.d. based on three times measurements with an excitation of 865 nm laser on spectrometer. (e) Ratio values deviation with different thickness pork blocking to ratio signals measured without pork blocking using the same wavelength or conventional ratiometric thermometry. (f) A plot of I_{PbS810}/I_{Tm810} versus T to calibrate the temperature. Average values of I_{PbS810}/I_{Tm810} under different temperature were given to fit the calibration curve based on the spectra with different thickness pork blocking. Error bars were defined as s.d.

Comment 3. *The experiments in vivo without UCL-NCs also showed a significantly surficial temperature-increment, which is not that different from the treatment group only estimating from the thermal images, but induced an entirely different result. Details are insufficient.*

Response: We appreciate this valuable advice. As shown in Figure S11, both the nude mice with and without injection of UCL-NCs show a little skin surficial temperature increasement. For the experiment group, about 3 ^oC skin surficial temperature increasement is observed, and for the control group, skin surficial temperature increasement is about 1 ^oC. In contrast to this, intratumoral temperature shows a sharp increment and increases to as high as 47 ^oC (Figure S13), which is high enough to produce tumor regression.

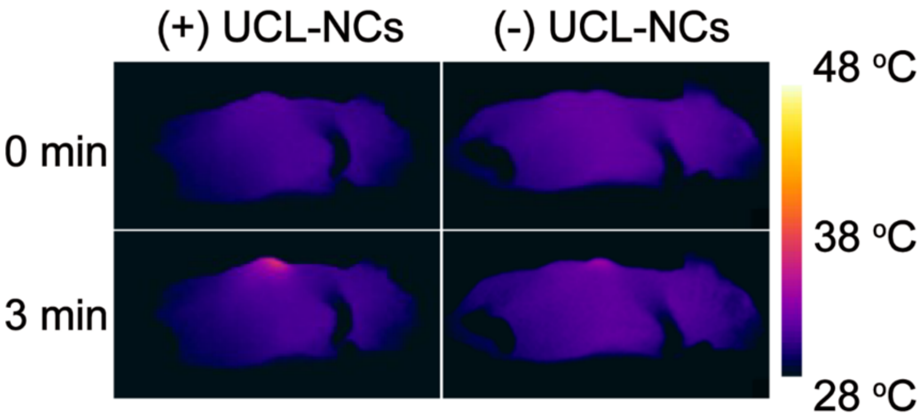

Figure S11. Thermal images of nude mice with (left panel) and without (right panel) injection of UCL-NCs under the 865-nm irradiation (0.5 W/cm^2).

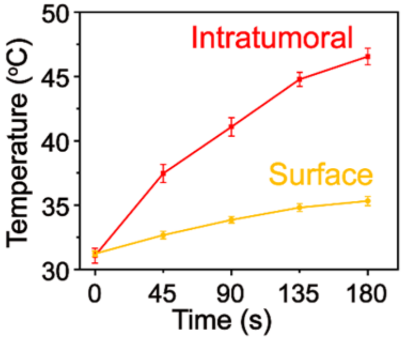

Figure S13. Intratumoral and skin surficial temperature measured during the PPT of the tumor area. The data were collected using the home built time-gate imaging system and a thermographic camera, respectively. Average values of intratumoral and skin surficial temperature at different time points were given based on three times measurement. Error bars were defined as s.d.

Comment 4. *With an approximate 8 nm-thick polyethylene glycol (PEG) modified silica shell, it demonstrated that the final UCL-NCs@SiO2 showed almost invisible increase and even shrink in size in TEM images Fig.2C and D.*

Response: We appreciate this valuable advice. The TEM images only demonstrate the sized of part nanoparticles. It is common that one or more nanoparticles have a smaller size. As shown in Fig. R1, UCL-NCs@SiO_2 shows about 20 nm size increment.

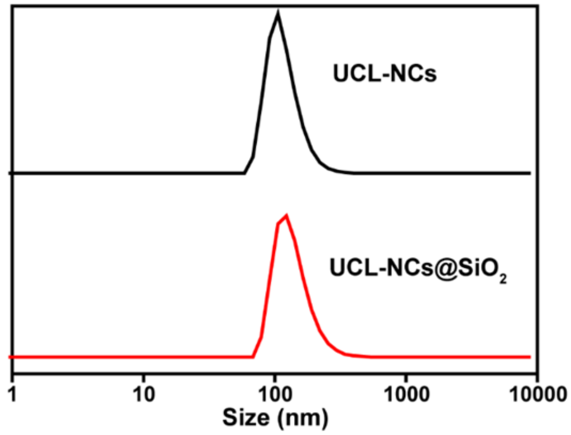

Figure R1. Dynamic light scattering data of UCL-NCs and UCL-NCs@SiO2.

Comment 5. *The data provided in Fig. S10 indicates the photothermal stability is not perfect, and much more information about the stability (photothermal stability and structural stability) is needed.*

Response: We appreciate this valuable advice. The photothermal stability and structural stability are evaluated in continuous warming-cooling cycles. The stable ratiometric signals and hydrodynamic sizes all indicate the good staility of this structure.

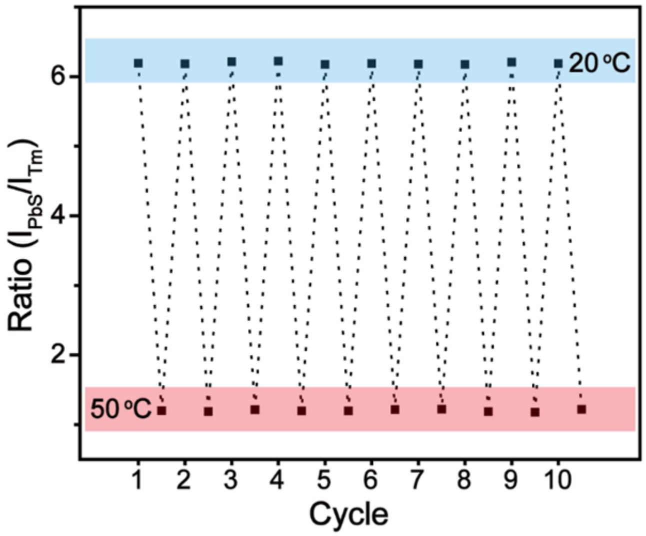

Figure S7. The reversibility of UCL-NCs tested in continuous warming-cooling cycles. Ratiometric signals were measured at 20 °C and 50 °C using a time gate spectrometer, respectively.

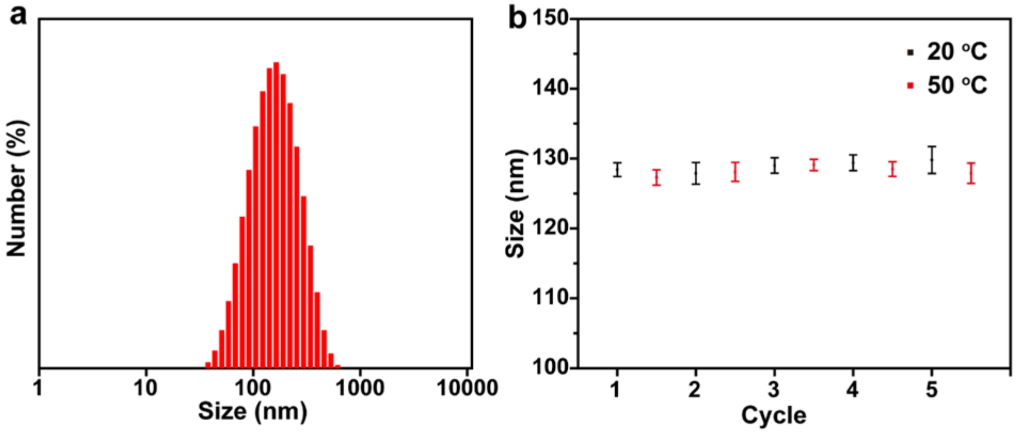

Figure S8. Heat stability of UCL-NCs evaluation. (a) The hydrodynamic sizes distribution of UCL-NCs measured by DLS analysis. (b) The hydrodynamic size of UCL-NCs measured at 20 or 50 ^{o}C in continuous warming-cooling cycles.

Reviewer #3:

Comments:

In this paper authors have developed a novel nanostructure for high sensitivity ratiometric thermal sensing. It is based on the combination of upconverting rare earth nanoparticles ad infrared emitting PbS QDs. The different quenching mechanisms of these structures makes possible thermal reading from a ratiometric approach. Instead of acquiring the ratiometric parameter from a simple analysis of the emission spectrum of their composites (that is the result of the superposition of the emission of UCNPs and PbS QDs) authors use time decoding to elucidate the relative contributions of these two structures. The thermal sensitivity obtained is quite good (5% per degree) and authors used this structures to perform very preliminary in vivo experiments.

The structure here reported is interesting but I can not see the clear contribution of this work to the state of the field. I think authors should discuss with more detail the advantajes of their approach in respect to what is already published on the field. For instance:

Comment 1. *It is not clear why time-decoding is beneficial in respect to a simple spectroscopic analysis. The thermal sensitivity curves should be given as obtained by applying time decoding and simple spectral anlysis if CW emission spectra.*

Response: We appreciate this valuable advice. In order to clearly stated the advantages of the way of measuring the ratiometric signal relatively to what is reported before, in our revised manuscript, control temperature monitoring experiments of conventional and this same wavelength ratiometric nanothermometry are performed in details using pork covering to simulate the situation *in vivo*. (Fig. 3 and 4). As shown in those results, high accuracy of this same wavelength ratiometric nanothermometry *in vivo* are marked exhibited, owing to eliminating errors caused from different wavelength ratio. In detail, for conventional ratiometric nanothermometry, the temperature uncertainty is 5.6 K which makes it unpractical to monitor the precise temperature in vivo. In contrast to this, for the same wavelength ratiometric nanothermometry, a temperature uncertainty of 0.3 K is obtained owing to eliminating the errors from the effect of heterogeneity on different wavelengths, which exhibits an overwhelming advantage in temperature monitoring in vivo.

As shown in Fig. 4b and Fig. 4c, $I_{real\text{-}time}$ of UCL-NCs contains both UCL emissions of UCNPs (long lifetime) and PbS QDs (short lifetime). Then I_{UCNPs} was obtained using a 20 μs time-gate to eliminate the short lifetime signal of PbS QDs. So I_{PbS} was calculated from $I_{real\text{-}time}$ minus $I_{time\text{-}resolved}$. And the corresponding plots calculated from time-resolved spectrometer are shown in Figure 4d.

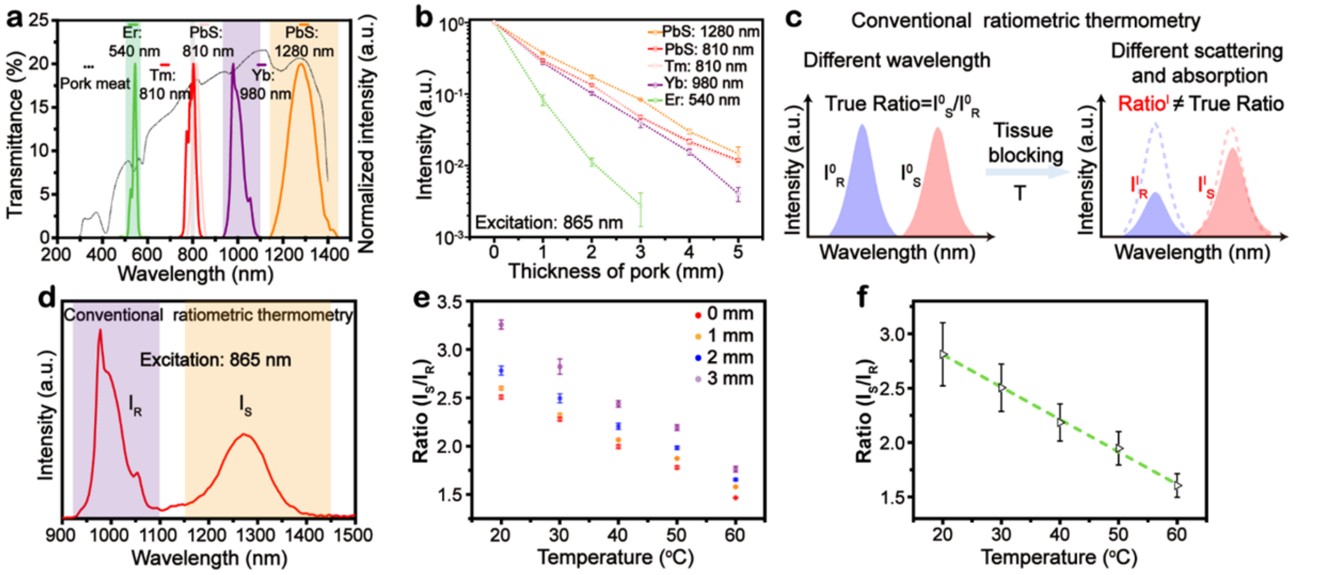

Fig. 3 Errors derived from the tissue blocking in conventional luminescent ratiometric thermometry. (a) Transmittance spectrum (left axis) of 1 mm pork and emission spectra (right axis) of Er-UCNPs, Tm-nanoparticles and PbS QDs. Colour shades correspond to the spectral ranges for collecting respective emissions. (b) Normalized intensities obtained from Er-UCNPs, Tm-nanoparticles and PbS QDs with respect to thickness of pork under the excitation of 865 nm laser. Each data and error bar represents mean ± s.d. based on three times measurements on spectrometer. (c) Schematic illustration of conventional luminescent ratiometric thermometry with different wavelength emission. (d) Stokes emission spectrum of UCL-NCs under the excitation of 865 nm laser. I_R and I_S are the reference emission (Yb^{3+} emission, 980 nm) and temperature sensitive emission (PbS emission, 1280 nm), respectively. (e) Ratios of I_S to I_R at different temperature with various thickness pork blocking. Each data and error bar represents mean ± s.d. based on three times measurements on spectrometer. (f) A plot of I_S/I_R versus T to calibrate the temperature. Average values of I_S/I_R under different temperature were obtained from the ratios with different thickness (0, 1, 2 and 3 mm) pork blocking. Error bars were defined as s.d.

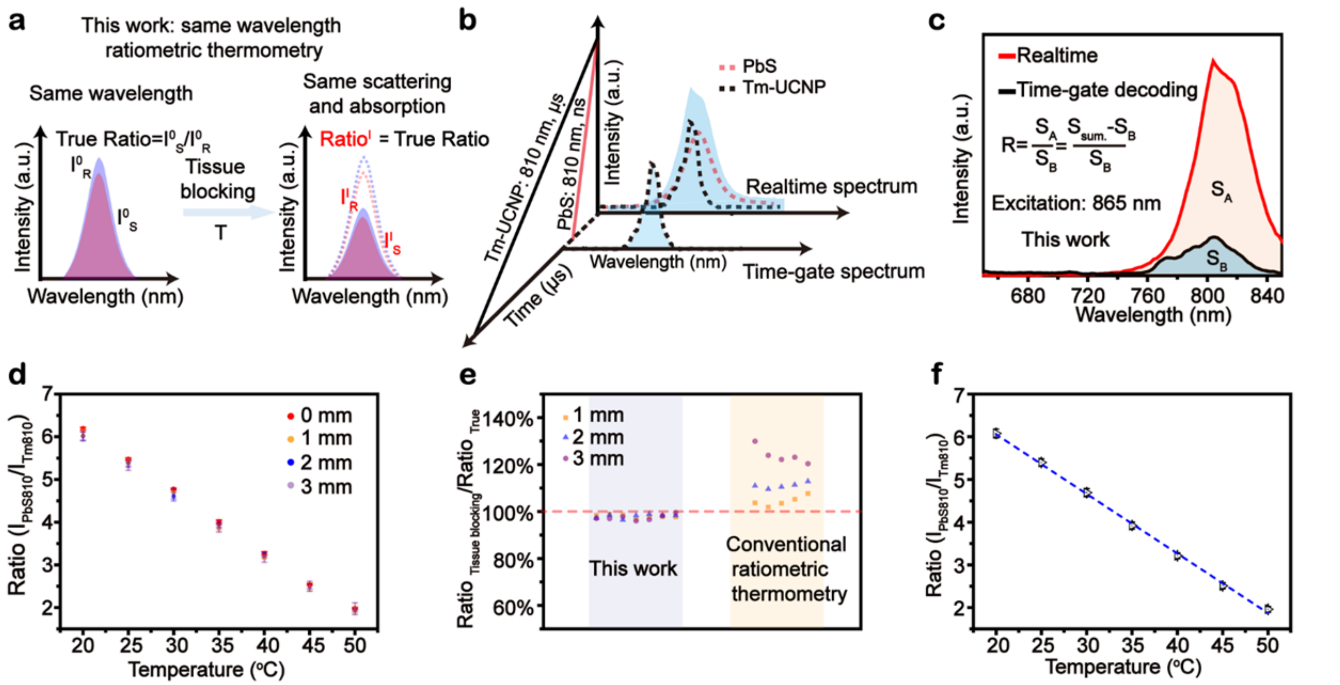

Fig. 4 Temperature monitoring ability evaluation of the same wavelength ratiometric thermometry based on the UCL emission of UCL-NCs. (a) Schematic illustration of conventional ratiometric thermometry with the same wavelength emission. (b) Schematic diagram of UCL signals separation via time-resolved decoding. (c) UCL emssion signals decoding of UCL-NCs using time-resolved spectrometer. (d) The same wavelength ratio of I_{PbS} to I_{Tm} obtained time-resolved spectrometer at different temperature with various thickness pork blocking. Each data and error bar represents mean ± s.d. based on three times measurements with an excitation of 865 nm laser on spectrometer. (e) Ratio values deviation with different thickness pork blocking to ratio signals measured without pork blocking using the same wavelength or conventional ratiometric thermometry. (f) A plot of I_{PbS810}/I_{Tm810} versus T to calibrate the temperature. Average values of I_{PbS810}/I_{Tm810} under different temperature were given to fit the calibration curve based on the spectra with different thickness pork blocking. Error bars were defined as s.d.

Comment 2. *The brightness of the here used nanostructures have to be given. What is the minimum excitation density that can be used to obtaine subtissue thermal sensing. How is related the thermal accuracy with the illumination intensity?*

Response: We appreciate this valuable advice. It is necessary for a ratiometric luminescent thermometer to investigate power dependent spectra both of sensor and reference signals, owing to the reduced power density of excitation light after penetrating the tissue. In principle, for UCL emission, I_{em} is proportional to the n-th power of the NIR exciting power density. The data demonstrate that the relationship between UCL intensity and excitation power density of PbS QDs is linear (Figure S5a). For Tm-UCNPs, the result indicates that UCL intensity is quadratic depending on the excitation power (Figure S5b). However, under a low power excitation, a linear relationship can be fitted in mathematics (Figure S5b inset). The relationship between excitation power density and I_{Tm} was investigated in detail sub-1 W/cm^2 (100-900 mW/cm^2) at 25 ^oC (Figure S5c). The result reveals that I_{Tm} can also be fitted to show linear dependence on excitation power density. It should be noticed that the ratio of I_{PbS} to I_{Tm} is invariant under different excitation power density at 25 ^oC (Figure S6). These results show that the illumination intensity sub-1 W/cm^2 (100-900 mW/cm^2) will not affect the thermal accuracy. And the temperature calibration curve is measured under an illumination intensity of 100 mW/cm^2.

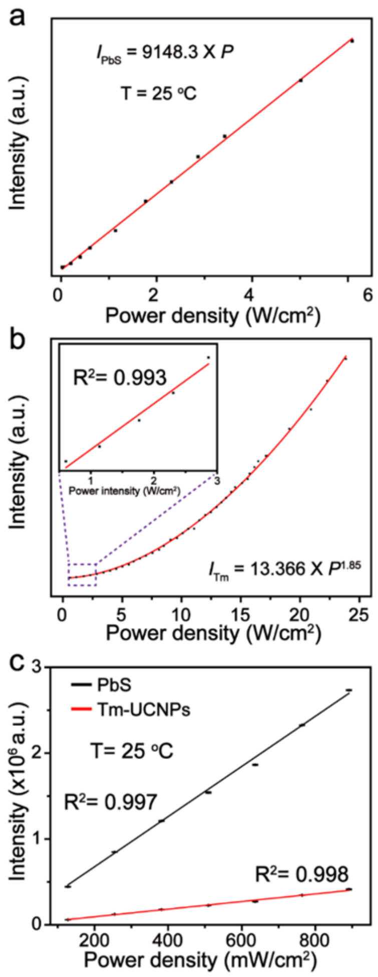

Figure S5. A plot of I_{UCL} versus P to investigate the effect of power density to UCL emission of (a) PbS and (b) Tm-UCNPs. The inset of (b) shows a linear dependent in mathematics under a low

power density excitation. (c) Plots of UCL emission of PbS QDs and Tm-UCNPs as a function of excitation power density of the 865 nm laser.

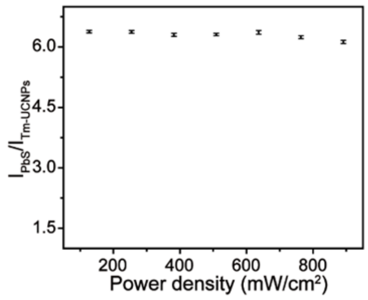

Figure S6. Ratios calculated from $I_{PbS}/I_{Tm\text{-}UCNPs}$ under different power density excitation at 25 ^oC.

Comment 3. *In the experimental data included in Figure 4e the time resolution seems to be of tens of seconds. Why is that? Is there any limitation not discussed that avoids real time thermal sensing based on this sensing system?*

Response: We appreciate this valuable advice. The time in Figure S13 is the irradiation time of 865 nm laser (0.5 W/cm^2) without modulation. The time-gate using in our manuscript is 20 μs. The limitation that avoids real time thermal sensing based on this sensing system is the penetration depth of tissue. When the thickness of tissue is greater than 3 mm, the errors increase owing to the sharply attenuating of the signals.

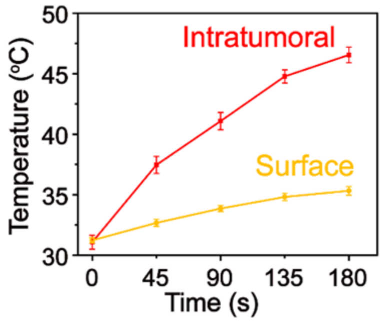

Figure S13. Intratumoral and skin surficial temperature measured during the PPT of the tumor area. The data were collected using the home built time-gate imaging system and a thermographic camera, respectively. Average values of intratumoral and skin surficial temperature at different time points were given based on three times measurement. Error bars were defined as s.d.

Comment 4. *What is exactly the heating source in their in vivo experiments? If it is associated to the heating efficiency of their nanostructures then this should be described and evaluated.*

Response: We appreciate this valuable advice. The exactly heating source in our in vivo experiments is an 865 nm laser (0.5 W/cm^2). The photothermal ability was just used to bring in a temperature change in vivo. As shown in Figure S10, the temperature increment is enough using an 865 nm laser (0.5 W/cm^2) as the heating source.

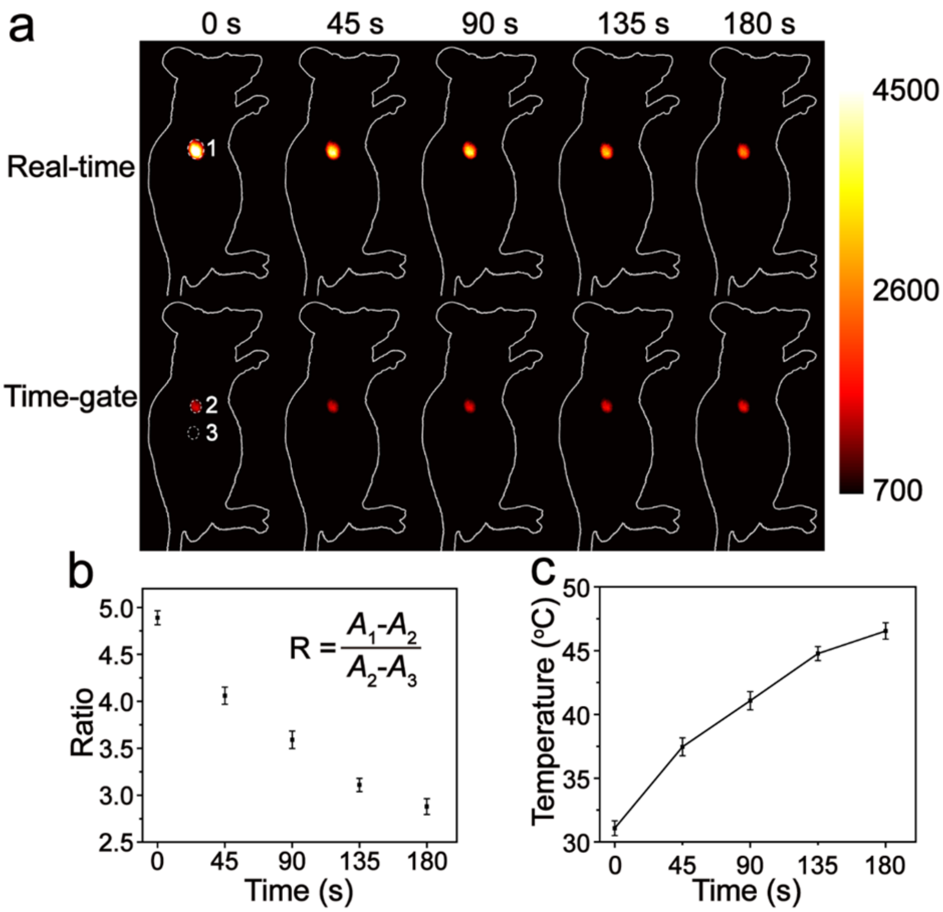

Figure S10. Intratumoral temperature monitoring *in vivo* during the PTT. (a) Real-time and time-gate UCL images of nude mice with the injection of UCL-NCs at different time under the irradiation of an 865 nm laser (0.5 W/cm^2). (b) Calculated ratio of I_{PbS} to $I_{Tm\text{-}UCNPs}$ obtained from figure (a). (c) The intratumoral temperature at different time during the PTT.

Comment 5. *What is the actual penetration depth of their structures?*

Response: We appreciate this valuable advice. The true depth of tissue is difficulty to determine owing to the wide distribution of the nanostructures *in vivo*. In order to eliminate the influence of light absorption and light scattering when penetrating the tissue, we developed this same wavelength ratiometric thermometry. For subcutaneous tumor in nude mouse, the penetration depth is supposed to sub 3 mm.

Reviewers' comments:

Reviewer #1 (Remarks to the Author):

The revised version of the manuscript was substantially improved and the innovative degree of the paper is now more evident. The data reported demonstrate for a specific example some advantages of the same wavelength ratiometric nanothermometry approach for in vivo experiments, relatively to the conventional technique of different wavelength ratio.

The reported thermal relative sensitivity and temperature uncertainty values are better than those reported up to now using the conventional approach, which is probably related to the tissues heterogeneity at different wavelengths. The conclusions, and in general the tone of the paper's message, are, however, excessive. In the studied hybrid heater-thermometer platform with PbS QDs and NaYbF4:0.5%Tm@NaYF4:10%Yb@NaYF4:50%Nd upconversion NPs the figures of merits of the same same wavelength ratiometric nanothermometry approach are higher but the extrapolation of these results for other systems is highly speculative. I strong recommend extreme caution in this regard. With this modification, I support the publication of the paper.

Reviewer #2 (Remarks to the Author):

In the manuscript, a new strategy to fabricate a ratiometric upconversion luminescence nanothermometry is provided based on a hybrid structure composed of upconversion emissive PbS quantum dots and NaYbF4:0.5%Tm@NaYF4:10%Yb@NaYF4:50%Nd nanoparticles. The ratiometric signal composed of two upconversion emissions working at the same wavelength but different luminescent lifetimes are decoded via a time-resolved technique. In the responses, the main point shown in this manuscript is emphasized to be the accurate detection of temperature in vivo. However both the detection of temperature in vivo and ratiometric nanothermometry have been reported previously. The originality of this manuscript is still not enough, and this manuscript should be published elsewhere.

Some points about the response may be useful.

1. It is illustrated that the same wavelength NIR emission ratio upconversion luminescent nanothermometry in this work eliminate f(a) and f(s), which cannot be eliminated by the dual emission ratio luminescent nanothermometers. While, its influences on f(p) and f(c), which could be eliminated by the dual emission ratio luminescent nanothermometers, are not mentioned. The data in Table 1 also proved that the dual emission ratio luminescent nanothermometers could exhibit relatively high thermal sensitivity. So, the response to comment 1, "could its sensor accuracy be further improved with both the two strategies combined" is not clear enough.
2. The dynamic light scattering data in Figure R1 demonstrated the sizes of UCL-NCs and UCL-NCs@SiO2, confirming the size increment after SiO2-PEG modification. However, it is not reasonable to show a representative TEM images with nanoparticles of smaller sizes.
3. TEM images of the nanoparticles before and after warming-cooling cycles may be more visualized to confirm the stability.
4. More details about the experiments are needed.

Reviewer #3 (Remarks to the Author):

Authors have properly address my previous comments. In my opinion the paper is ready for publication.

Responses to the Reviewers' Reports

Reviewer #1:

The revised version of the manuscript was substantially improved and the innovative degree of the paper is now more evident. The data reported demonstrate for a specific example some advantages of the same wavelength ratiometric nanothermometry approach for in vivo experiments, relatively to the conventional technique of different wavelength ratio.

The reported thermal relative sensitivity and temperature uncertainty values are better than those reported up to now using the conventional approach, which is probably related to the tissues heterogeneity at different wavelengths. The conclusions, and in general the tone of the paper's message, are, however, excessive. In the studied hybrid heater-thermometer platform with PbS QDs and NaYbF4:0.5%Tm@NaYF4:10%Yb@NaYF4:50%Nd upconversion NPs the figures of merits of the same wavelength ratiometric nanothermometry approach are higher but the extrapolation of these results for other systems is highly speculative. I strong recommend extreme caution in this regard. With this modification, I support the publication of the paper.

Response: We are very grateful to the reviewer for the positive evaluation and helpful suggestions. According to the reviewer's suggestion, we have modified some sentences that express excessive in our manuscript, especially in the discussion section. We have also paid attention to the wording of the article and modified the way of expression. Moreover, we deleted the extrapolation of our results for other systems in the discussion section. Details are highlight in our revised manuscript and shown as following.

In the manuscript:

"In order to minimize these errors and monitor temperature *in vivo*" (P.1, Line 10)

"This nanothermometer improves the temperature monitoring ability and a thermal resolution of ~0.3 K and a thermal sensitivity of ~5.6% K^{-1} is obtained *in vivo*" (P.1, Line 15)

"owing to reducing the impact of extrinsic factors like sensor concentration" (P.2, Line 22)

"Owing to minimizing the influence of $f(p)$ and $f(c)$" (P.2, Line 24)

"However, f(a) and f(s) which depend on wavelength still cannot be avoided owing to the great difference in wavelength between the ratiometric emission" (P.2, Line 26)

"To minimize the errors originating from different wavelength in dual emission ratio nanothermometer" (P.3, Line 11)

"This dual emission ratiometric nanothermometry demonstrates a good temperature measurement ability in tissue and temperature mapping in tumor region is performed on mice *in vivo.*" (P.4, Line 4)

"a different attenuation is observed between 980 nm (Yb^{3+} emission) and 1280 nm" (PbS QD emission) (P.6, Line 10)

"these results suggest that it remains difficult for the different wavelength ratiometric thermometry to monitor the temperature accurately *in vivo*." (P.7, Line 22)

"the temperature calibration curve of UCL-NCs was investigated via performing several heating and cooling cycles on a time-resolved spectrometer." (P.9, Line 5)

"This result indicates that the same wavelength dual emissin ratiometric upconversion thermometry shows an excellent temperature monitoring ability and the errors origins from tissue blocking can be reduced." (P.11, Line 23)

"Therefore, the UCL-NC probe is capable of thermometry in vivo for monitoring intratumoral temperature." (P.12, Line 1)

Discussion

In summary, we demonstrated a same wavelength dual emission ratio upconversion luminescent nanothermometry system, which exhibits a more precise temperature detection *in vivo* and applied in the intratumoral temperature monitoring. Under the excitation of 865 nm laser, PbS QDs give a short lifetime temperature response UCL emission around 810 nm via single photon process and the Nd-sensitized Tm-UCNPs exhibit a long lifetime reference UCL signal around 810 nm. These two UCL emission at the same wavelength can be separated via time-resolved decoding owing to the different lifetime. The same wavelengths of excitation and emission ratiometric nanothermometer based on UCL-NCs shows an improved temperature measurement accuracy *in vivo*, and a temperature uncertainty *in vivo* of 0.3 K is obtained. It is worth noting that the thermal sensitivity of this hybrid system is up to 5.6 % K^{-1}. Furthermore, the NIR excitation and emission have advantages in biological applications. After being modifying with PEG, this hybrid nanostructure material was used in intratumoral temperature monitoring *in vivo* during the photothermal therapy. Thus, the present work reports a more accurate upconversion luminescent nanothermometry and provides a new way in the construction the dual emission luminescent nanothermometer.

Reviewer #2:

In the manuscript, a new strategy to fabricate a ratiometric upconversion luminescence nanothermometry is provided based on a hybrid structure composed of upconversion emissive PbS quantum dots and $NaYbF_4$:0.5%Tm@$NaYF_4$:10%Yb@$NaYF_4$:50%Nd nanoparticles. The ratiometric signal composed of two upconversion emissions working at the same wavelength but different luminescent lifetimes are decoded via a time-resolved technique. In the responses, the main point shown in this manuscript is emphasized to be the accurate detection of temperature in vivo. However both the detection of temperature in vivo and ratiometric nanothermometry have been reported previously. The originality of this manuscript is still not enough, and this manuscript should be published elsewhere.

Some points about the response may be useful.

Response: We appreciate many helpful and valuable comments. In the revised manuscript, we have fully responded this comment as well as addressed the four comments by making supplementary experiments (*vide infra*). We are indebted to the Reviewer #2 for such remarkable improvement of our manuscript based on his/her comments. We regret however our revised manuscript could not convince Reviewer#2 of the originality in this work. Unfortunately, Reviewer #2 considered that the main point of our present work is simply emphasized to be the accurate detection of temperature *in vivo* using ratiometric nanothermometry, which has been reported previously. Previous ratio temperature probes use filters to separate the temperature sensitive signal from the reference signal, which requires a large difference in wavelength between the two ratio signals. In these cases, it would cause errors due to different tissue absorption and scattering deriving from wavelength difference. In order to address this issue, our current work uses two same-wavelength signals with different lifetime to construct a dual-emission luminescence nanothermometers, which can furtherly improve the accuracy of temperature measurement.

Comment 1: It is illustrated that the same wavelength NIR emission ratio upconversion luminescent nanothermometry in this work eliminate f(a) and f(s), which cannot be eliminated by

the dual emission ratio luminescent nanothermometers. While, its influences on f(p) and f(c), which could be eliminated by the dual emission ratio luminescent nanothermometers, are not mentioned. The data in Table 1 also proved that the dual emission ratio luminescent nanothermometers could exhibit relatively high thermal sensitivity. So, the response to comment 1, "could its sensor accuracy be further improved with both the two strategies combined" is not clear enough.

Response: We are very sorry for our unclear expression on the same wavelength dual emission ratiometric nanothermometry. Our present work is based on the previous dual emission ratiometric nanothermometer, what's the improvement of our nanothermometer is that we use two same wavelength emission as the ratiometric signals. Previous dual emission ratiometric nanothermometry use filters to separate the temperature sensitive signal from the reference signal, so it is unpractical to choose two emission at the same wavelength to fabricate a dual emission ratiometric nanothermometry. However, f(a) and f(s) which depend strongly on wavelength still cannot be eliminated owing to the difference in wavelength between the ratiometric emission. To minimize the influence of f(a) and f(s), in our present work, we chose two emission at the same wavelength with different lifetime to fabricate the dual emission ratiometric nanothermometry. The two signals were separated using time-resolved technique owing to their different lifetime. So our nanothermometer still has the merits of previous dual emission ratiometric nanothermometers and the influences of f(c) can be eliminated owing to the constant ratio (I_{PbS}/I_{Tm}) of different concentration. What's more, the influences of f(p) can also be minimized under a low excitation power density sub-1 W/cm^2 as shown in Figure S5 and S6. The key point of our present work is that the influences of f(a) and f(s) originating from wavelength difference of the ratiometric emission can be minimized.

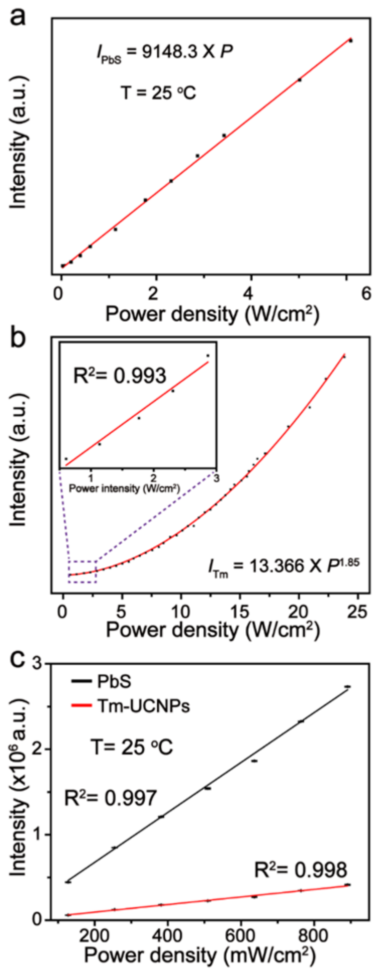

Figure S5. A plot of I_{UCL} versus P to investigate the effect of power density to UCL emission of (a) PbS and (b) Tm-UCNPs. The inset of (b) shows a linear dependent in mathematics under a low power density excitation. (c) Plots of UCL emission of PbS QDs and Tm-UCNPs as a function of excitation power density of the 865 nm laser.

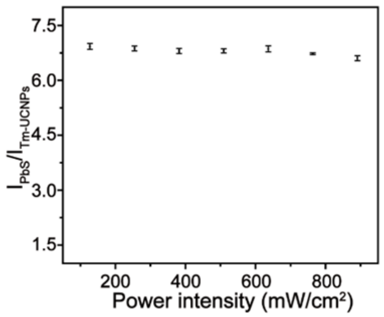

Figure S6. Ratios calculated from UCL emission of I_{PbS} to $I_{Tm-UCNPs}$ under different power density excitation at 25 ^oC.

It should also be mentioned that the signals of different-wavelength dual emission ratio nanothermometer also can be separated using time-resolved technique. As shown in Figure R1, I_{sum} composed of long-lifetime I_1^0 and short-lifetime I_2^0 can be obtained from a real-time spectrum

or imaging. I_1' (I_1'=a × I_1^0, a<1) can be separated using a time-resolved technique owing to the different lifetime. Then I_2^0 can be calculated: I_2^0 =I_{sum} - I_1'/a. At last, ratio of I_2^0 to I_1^0 can be calculate as following:

$$R\left(\frac{I_2^0}{I_1^0}\right) = \frac{I_{sum} - \frac{I_1'}{a}}{\frac{I_1'}{a}} = a\frac{I_{sum}}{I_1'} - 1$$

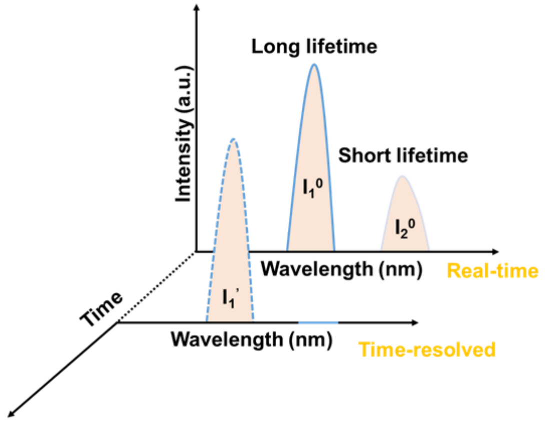

Figure R1. Schematic figure of separating signals of different-wavelength dual emission ratio nanothermometer using time-resolved technique.

The merit of time-resolved technique to separate two signals is that the ratiometric emission is no longer limited by wavelength, and using time-resolved technique to separate two signals with different wavelength shows no obvious merit comparing to using a filter. The advantages of time-resolved technique can be reflected in separating two signals with very close or same wavelength, because the influences of f(a) and f(s) originating from wavelength difference of the ratiometric emission can be minimized.

So the answer to "could its sensor accuracy be further improved with both the two strategies combined" is that: **the sensor accuracy can be further improved with both the two strategies combined when the ratiometric signals have two emission with very close or same wavelength like our present work.** If using two different wavelength emission as ratiometric signals, time-resolved technique shows no obvious merits comparing to using filter.

In order to express the advantages of our same-wavelength dual emission ratio nanothermometer clearly, we have emphasized the advantages of dual emission ratio luminescent nanothermometers and made some changes in our revised manuscript as following:

"Dual emission ratio luminescent nanothermometers show resolvable emission from two different emitters, which can be used as a self-referencing ratio. The temperature sensitive signal change can be measured using ratio instead of absolute photoluminescence intensities, owing to reducing the impact of extrinsic factors like sensor concentration ($f(c)$), fluctuations of excitation power density ($f(p)$) or other local inhomogeneities that alter absolute intensities. Owing to minimizing the influence of $f(p)$ and $f(c)$, the dual emission ratio luminescent nanothermometers have been widely developed and attracted considerable interest." (P.2, Line 19)

"we provide a new strategy to fabricate the same-wavelength dual emission ratiometric upconversion" (P.1, Line 11)

“the same wavelength dual emission ratio upconversion luminescent nanothermometry” (P.3, Line 11)

“This dual emission ratiometric nanothermometry demonstrates a good temperature measurement ability in tissue and temperature mapping in tumor region is performed on mice *in vivo.*” (P.4, Line 4)

“Same wavelength dual emission ratiometric thermometry in vitro based on UCL-NCs. ” (P.8, Line 13)

“the same wavelength dual emission ratiometric” (P.11, Line 1)

“This result indicates that the same wavelength dual emission ratiometric upconversion thermometry shows an excellent temperature monitoring ability and the errors origins from tissue blocking can be reduced.” (P.11, Line 23)

“In summary, we demonstrated a same wavelength dual emission ratio upconversion luminescent nanothermometry system, which exhibits a more precise temperature detection *in vivo* and applied in the intratumoral temperature monitoring.” (P.13, Line 4)

Comment 2: The dynamic light scattering data in Figure R1 demonstrated the sizes of UCL-NCs and UCL-NCs@SiO_2, confirming the size increment after SiO_2-PEG modification. However, it is not reasonable to show a representative TEM images with nanoparticles of smaller sizes.

Response: We appreciate the valuable and helpful comment. A new TEM image of UCL-NCs@SiO_2 was using in Figure 2. The average size of UCL-NCs is 94.3 ± 6.1 nm, and after coating a SiO_2 shell, the size increases to 114.1 ± 8.9 nm, which is agreement with the DLS result (Figure R2). For further confirming the successful coating of SiO_2, the EDS mapping of ULC-NCs@SiO_2 was performed. The result reveals a clear SiO_2 shell outside the UCL-NC.

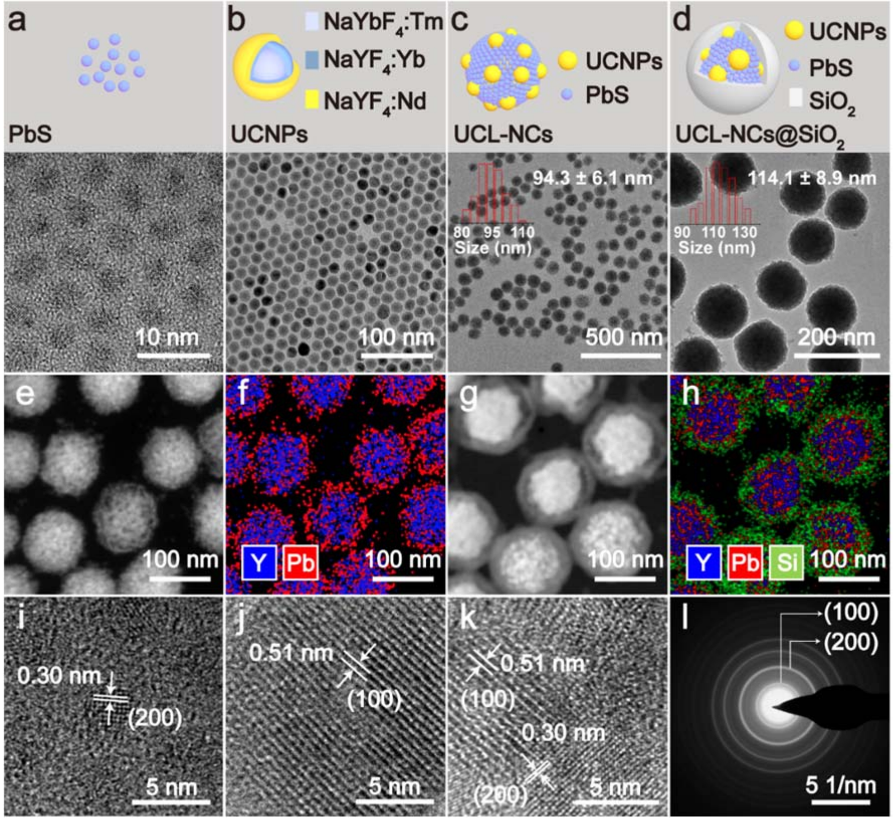

Figure 2. Characterizations of UCL-NCs. TEM images of (a) PbS QDs, (b) core-shell-shell Nd sensitized Tm-UCNPs, (c) UCL-NCs and (d) mPEG-silane functionalized silica-coated UCL-NCs. Images of dark-field scanning TEM and corresponding EDS elemental mapping of (e and f) UCL-NCs and (g and h) UCL-NCs@SiO_2. HR-TEM images of (i) PbS QDs, (j) core-shell-shell Nd sensitized Tm-UCNPs and (k) UCL-NCs. (l) Electron diffraction image of UCL-NCs.

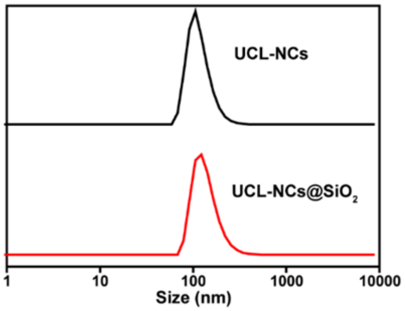

Figure R2. Dynamic light scattering data of UCL-NCs and UCL-NCs@SiO_2.

Comment 3: TEM images of the nanoparticles before and after warming-cooling cycles may be more visualized to confirm the stability.

Response: We appreciate this important and helpful comment. According to the reviewer's recommendation, TEM image (Figure S9a), dark-field scanning TEM image (Figure S9b) and corresponding EDS elemental mapping (Figure S9c-f) of UCL-NCs@SiO_2 after warming-cooling cycles were performed. These results exhibit no obvious difference with the nanoparticles before warming-cooling cycles (Figure 2d, 2g and 2h), which reveals the stability of this structure.

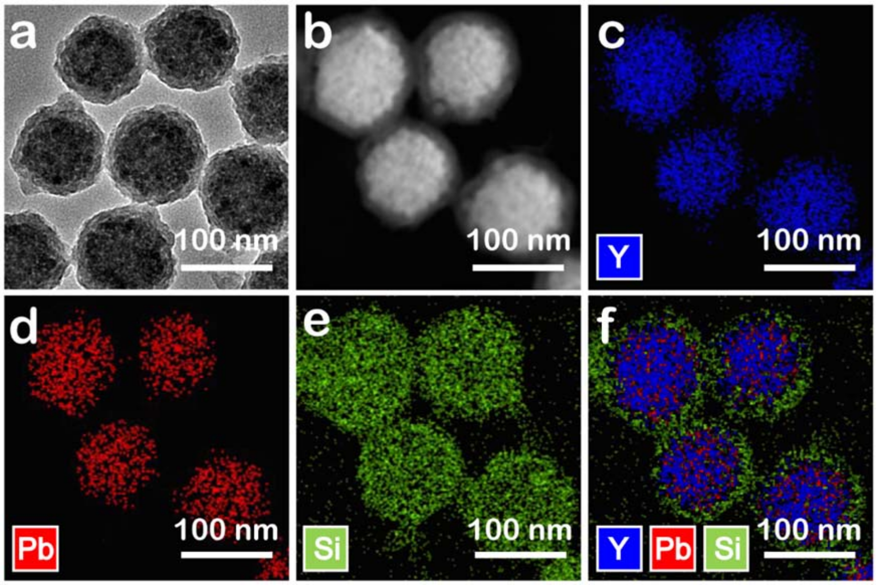

Figure S9. TEM image, dark-field scanning TEM image and corresponding EDS elemental mapping of (c, d, e and f) UCL-NCs@SiO_2 after warming-cooling cycles.

Comment 4: More details about the experiments are needed.

Response: We appreciate this important and helpful comment. According to the reviewer's suggestion, we have added further details about the experiments as below:

Temperature monitoring *in vitro*. To obtain the same wavelength ratiometric temperature calibration curve in aqueous solution, a quartz cuvette containing UCL-NCs@SiO_2 aqueous dispersion (2 ml, 0.5 mg/ml) was placed in home-built time-resolved spectrometer or home-built time-resolved bioimaging system (emICCD camera, PI-MAX4-1024B/EM, Princeton Instruments) with an external temperature controller. Aqueous solution was heated to different temperature from 20 to 50 ^{o}C and the corresponding real-time or time-resolved UCL round 810 nm was collect with excitation of a continuous wave (CW) 865 nm laser (100 mW/cm^2). The ratio of UCL emission peaks centred at 810 nm of PbS QDs and Tm-UCNPs as function of temperature are used as calibration curve for temperature monitoring. Different thickness of pork meat covering was used to simulate the tissue blocking *in vivo*. The conventional ratiometric temperature calibration curves were obtained on Edinburgh FLS-920 fluorescence spectrometer under the irradiation of an 865 nm laser (100 mW/cm^2).

Tumour xenografts. Animal procedures were in agreement with the guidelines of the Institutional Animal Care and Use Committee, School of Pharmacy, Fudan University. S180 cells were harvested by incubation with 0.05% trypsine-EDTA when they reached near confluence, and were collected by centrifugation and re-suspended in sterile PBS. Cells (~10^7 cells per site) in 50 μL in modified Eagle's medium were then subcutaneously implanted into 4-week-old male

athymic nude mice. Photothermal therapy was performed when tumors reached 0.6 cm in average diameter.

Eigen temperature monitoring *in vivo*. Eigen temperature monitoring in vivo was conducted in UCL-NCs@SiO2 injected S180 tumour-bearing nude mice. 0–5W adjustable CW 865 nm lasers was chosen as the excitation source of UCL and photothermal excitation (0.5 W/cm^2). Real-time and time-gate UCL imaging were performed on a homebuilt time-resolved bioimaging system (emICCD camera, PI-MAX4-1024B/EM, Princeton Instruments) with an 810 ± 10 nm band-pass filter at different time point. The intratumoral temperature at different time point were calculated by the UCL images with the temperature calibration curve got in vitro simulated with pork meat covering.

Photothermal therapy *in vivo*. An optical fibre-coupled 865 nm diode-laser (Changchun New Industries Optoelectronics Technology Co., Ltd.) was used to irradiate tumours during the experiments. For treatment group, 50 μL of UCL-NCs@SiO_2 (2 mg/mL) aqueous solution was injected via intratumoral injection 2 hours before the irradiation. For photothermal treatment, the 865 nm laser beam with a diameter of ~10 mm was focused on the tumour area at the power density of 0.5 W/cm^2 for 3 min. FLIR E40 thermal imaging camera was used to collect Infrared thermal images. Tumour sizes of treatment group and reference groups (untreated mice, mice irradiated to the 865 nm laser only, and UCL-NCs@SiO_2 treated mice without 865 nm irradiation) were measured every day after treatment. Each group contained five mice for relatively rational evaluation. The tumour sizes were measured using a vernier caliper. Then the volume of tumor was calculated as following: volume = (tumour length) × (tumour width)^2/2. Relative tumour volumes were normalized and were calculated as V/V_0 (V_0 is the initiated tumour volume).

Characterization: TEM images, HRTEM images, HAADF-STEM images, and EDX mapping of the nanocrystals were characterized on a Tecnai G2 F20 S-Twin (FEI, America) operating at 200 kV. Fourier-transform infrared spectroscopy was performed using an IR Prestige-21 spectrometer (Shimadzu) from samples in KBr pellets. DLS was carried out on a Malvern Zetasizer Nano ZS system. UCL spectra of UCL-NCs were measured with an Edinburgh LFS-920 fluorescence spectrometer. Infrared thermal images were collected FLIR E40 thermal imaging camera.

Reviewer #3 (Remarks to the Author):

Authors have properly address my previous comments. In my opinion the paper is ready for publication

Response: We are very grateful to the reviewer for his/her positive evaluation.

REVIEWERS' COMMENTS:

Reviewer #1 (Remarks to the Author):

Authors have properly address my previous comments. In my opinion the paper is ready for publication.
